# Ecological drivers of taxonomic, functional, and phylogenetic diversity of bryophytes in an oceanic island

**DOI:** 10.1002/ece3.70023

**Published:** 2024-07-24

**Authors:** Anabela Martins, Flavien Collart, Manuela Sim‐Sim, Jairo Patiño

**Affiliations:** ^1^ cE3c—Centre for Ecology, Evolution and Environmental Changes & CHANGE—Global Change and Sustainability Institute/MUHNAC—Museu Nacional de História Natural e da Ciência Universidade de Lisboa Lisboa Portugal; ^2^ Department of Ecology and Evolution University of Lausanne Lausanne Switzerland; ^3^ Departamento de Biologia Vegetal, Faculdade de Ciências, cE3c—Centre for Ecology, Evolution and Environmental Changes & CHANGE—Global Change and Sustainability Institute Universidade de Lisboa Lisboa Portugal; ^4^ Island Ecology and Evolution Research Group, Instituto de Productos Naturales y Agrobiología (IPNA‐CSIC) La Laguna Spain; ^5^ Departamento de Botánica, Ecología y Fisiología Vegetal Universidad de La Laguna La Laguna Spain

**Keywords:** alpha diversity, beta diversity, community assembly, diversity facets, generalized dissimilarity model, life‐history traits, linear mixed‐effects model, Madeira Island

## Abstract

Montane oceanic islands possess unique geographic and ecological attributes, rendering them valuable for assessing patterns and drivers of alpha and beta taxonomic, functional, and phylogenetic diversity along elevational gradients. Such comparisons of diversity facets can provide insights into the mechanisms governing community assembly on islands. Herein, we aimed to characterize taxonomic, functional, and phylogenetic bryophyte diversity on Madeira Island within and across areas at varying elevations. We also assessed how these diversity facets for the alpha and beta components relate to ecological and anthropogenic factors. We estimated and compared alpha and beta taxonomic, functional, and phylogenetic diversity using 80 plots of 0.5 m × 0.5 m across the whole elevational gradient of the island. We compiled trait databases and supplemented them with our own observations. Phylogenetic information was sourced from the Moss and Liverwort Tree of Life. To assess the impact of ecological and anthropogenic factors on the three facets, we applied linear mixed‐effects models and generalized dissimilarity models to alpha‐ and beta‐diversity matrices, respectively. All facets of diversity exhibited strong correlations within both mosses and liverworts, indicating a substantial congruence when alpha and beta are analyzed separately. The bryophyte groups categorized by the growth form demonstrated contrasting patterns, aligning with their distinctive ecological requirements. While a mid‐elevation peak emerged as a common pattern across the three facets of alpha diversity, beta diversity often displayed the opposite trend. Although the relative influence of environmental factors varied depending on the diversity facet and bryophyte grouping considered, we found that alpha and beta diversity of bryophytes are more influenced by climatic factors and the predominant type of vegetation than by anthropogenic factors. In the current context of global change, these results should be interpreted with caution, but they point to the resilience of bryophytes to survive in relatively well‐preserved natural microhabitats within anthropogenic landscapes. In this study on Madeira Island, we investigated patterns and drivers of alpha and beta taxonomic, functional, and phylogenetic diversity along elevational gradients. We found that alpha and beta diversity of bryophytes are more strongly influenced by climatic factors and the predominant type of vegetation than by anthropogenic factors.

## Graphical abstract

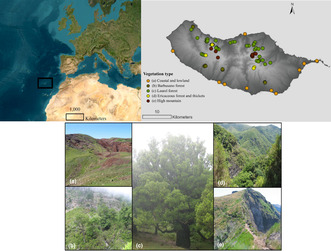

## 1 INTRODUCTION

Biodiversity refers to a complex, multifaceted concept that encompasses different levels of organization, from genes to species and communities to landscapes (Gaston, 1996; Swenson, 2011). Oceanic islands have played a crucial role in the study of how biodiversity is distributed across spatial, temporal, and environmental gradients and the underlying ecological and evolutionary processes (Carlquist, 1965; Darwin, 1859; MacArthur & Wilson, 1967; Whittaker et al., 2008; Whittaker & Fernández‐Palacios, 2007). Traditionally, island biodiversity research has been mostly focused on taxonomic diversity (hereafter TD; i.e., the total number of species). However, such an approach provides an incomplete and biased view of community composition, as well as of the primary mechanisms governing community assembly and evolution on islands (e.g., Schrader et al., 2021; Whittaker et al., 2017).

Over the past decade, there has been a notable increase in island studies that have examined complementary facets of biodiversity (e.g., König et al., 2021; Matthews et al., 2020; Ottaviani et al., 2020; Si et al., 2017; Weigelt et al., 2015; Whittaker et al., 2014). These include functional diversity (FD; i.e., the extent of functional trait variation between species) and phylogenetic diversity (PD; i.e., the extent of evolutionary divergence between species). Both FD and PD contribute not only to understanding habitat preferences and resource utilization but also to delineating the cumulative evolutionary history of communities. These are crucial factors in addressing unresolved questions in island biogeography (Patiño, Hedenäs et al., 2017; Whittaker et al., 2017) and in formulating more effective strategies for biodiversity conservation (Le Bagousse‐Pinguet et al., 2019).

Hypotheses regarding potential interactions between FD and PD have been also formulated. For instance, a strong and positive correlation between FD and PD is expected when functional traits enabling species to thrive in a particular environment are evolutionarily conserved (Ackerly, 2009; Donoghue, 2008; Emerson & Gillespie, 2008). Nevertheless, no relationship or even a negative one between FD and PD may be expected under strong competitive ecological dynamics (Emerson & Gillespie, 2008; Schluter, 2000). Moreover, the anticipated relationships between TD and FD, as well as TD and PD, are expected to be positive. This is because increasing the number of species within a community should not reduce either its variety of functional traits or its representation of the phylogenetic tree. On the contrary, communities with an identical number of species can still have species that differ in both their functional characteristics and their evolutionary relationships to varying degrees (Cadotte et al., 2013; De Pauw et al., 2021; Tucker et al., 2017). Therefore, available evidence has unveiled patterns of either discordance or congruence among diversity facets, which are often linked to underlying processes governing community assembly.

Breaking down the three diversity facets into local (alpha diversity hereafter termed α‐diversity) and regional (beta diversity hereafter termed β‐diversity) components (Whittaker, 1960) can be complementarily valuable for understanding community assembly processes (Baselga, 2010; Jost, 2007) and untangling the relative role of environmental, biological, and anthropogenic factors in shaping biodiversity patterns (Cabral et al., 2014; Patiño, Whittaker et al., 2017). As an example, Mouton et al. (2023) recently explored the variation of β‐TD across various spatial scales within the Macaronesian region (sensu Fernández‐Palacios et al., 2023) by a comparative analysis of land plant groups, including bryophytes, pteridophytes, and spermatophytes. They concluded that climate significantly influences β‐TD in the more dispersal‐prone bryophytes and pteridophytes, while factors such as island age, geographic distance, and archipelago structure—all likely associated with dispersal limitations—were more critical for spermatophytes (Mouton et al., 2023). Similar findings were drawn from the analyses of α‐TD patterns across Macaronesian archipelagos and plant groups (Aranda et al., 2013, 2014).

Bryophytes are categorized into three major evolutionary lineages, including mosses, liverworts, and hornworts. Within these lineages, mosses and liverworts are further divided based on their growth forms. Mosses are categorized as either acrocarpous or pleurocarpous, while liverworts are distinguished as either thalloid or leafy. These growth forms represent specific adaptations to general climatic regimes (Kürschner et al., 1998; Tuba et al., 2011) and responses to disturbances (During, 1992). Generally, leafy liverworts and pleurocarpous mosses are prevalent in shaded and stable environments. Nevertheless, they exhibit higher vulnerability to drought and disturbances induced by human activities when compared to acrocarpous mosses and thalloid liverworts. The latter are more commonly observed in arid conditions and anthropogenically modified habitats (Collart et al., 2021; During, 1992; Kürschner et al., 1999; Patiño et al., 2009; Tuba et al., 2011). In this context, bryophytes represent an ideal group for studying patterns of biodiversity facets across environmental and anthropogenic gradients.

Increasing evidence from studies focusing on functional traits, enhanced by the rise of databases dedicated to life‐history traits (Henriques et al., 2017; van Zuijlen et al., 2023), supports the idea that TD and FD are complementary facets in bryophytes (Ah‐Peng et al., 2014; Berdugo & Dovciak, 2019; Henriques et al., 2017; Spitale, 2016). While sequencing technologies are becoming more affordable, accessible, and increasingly applied to bryophyte taxa (Patiño et al., 2022), the number of bryological studies addressing PD patterns and their relationship with other facets of biodiversity remains comparatively low (Collart et al., 2021; Sanbonmatsu & Spalink, 2022; Shaw et al., 2005; Shen et al., 2022), namely on islands (Patiño et al., 2022; Patiño & Vanderpoorten, 2021). Indeed, due to the restricted availability of functional and phylogenetic data in macro‐ and microecological studies (Patiño et al., 2022), there is a possibility that key environmental and anthropogenic factors influencing the distribution of bryophytes have not been thoroughly captured yet.

We aim to address this gap by investigating how α‐ and β‐components of TD, FD, and PD are interrelated across an elevational gradient and to what extent they exhibit varying responses to geographic, ecological, and anthropogenic factors. As a study system, we focus on the main lineages and growth forms within bryophytes on Madeira, a montane, topographically complex oceanic island. Elevational gradients in irregular terrains create significant ecological variation for plant communities. These variations are evident at both local and regional levels, occurring on islands and across their habitats (Patiño et al., 2023; Steinbauer et al., 2016). Moreover, they have been extensively used as a space‐for‐time substitution approach in multifaceted biodiversity research, facilitating the examination of environmental (Bello et al., 2012; Chun & Lee, 2017; Schmucki et al., 2012) and land‐use effects (Penjor et al., 2021; Wesuls et al., 2011). Despite Madeira being a volcanic island with a relatively small area (742 km^2^), it features (i) a significant range in elevation (from sea level to 1862 m); (ii) a diverse array of climatic conditions; (iii) several distinct vegetation zones; and (iv) a wide variety of human disturbances, including wildfires and biological invasions.

**FIGURE 1:**
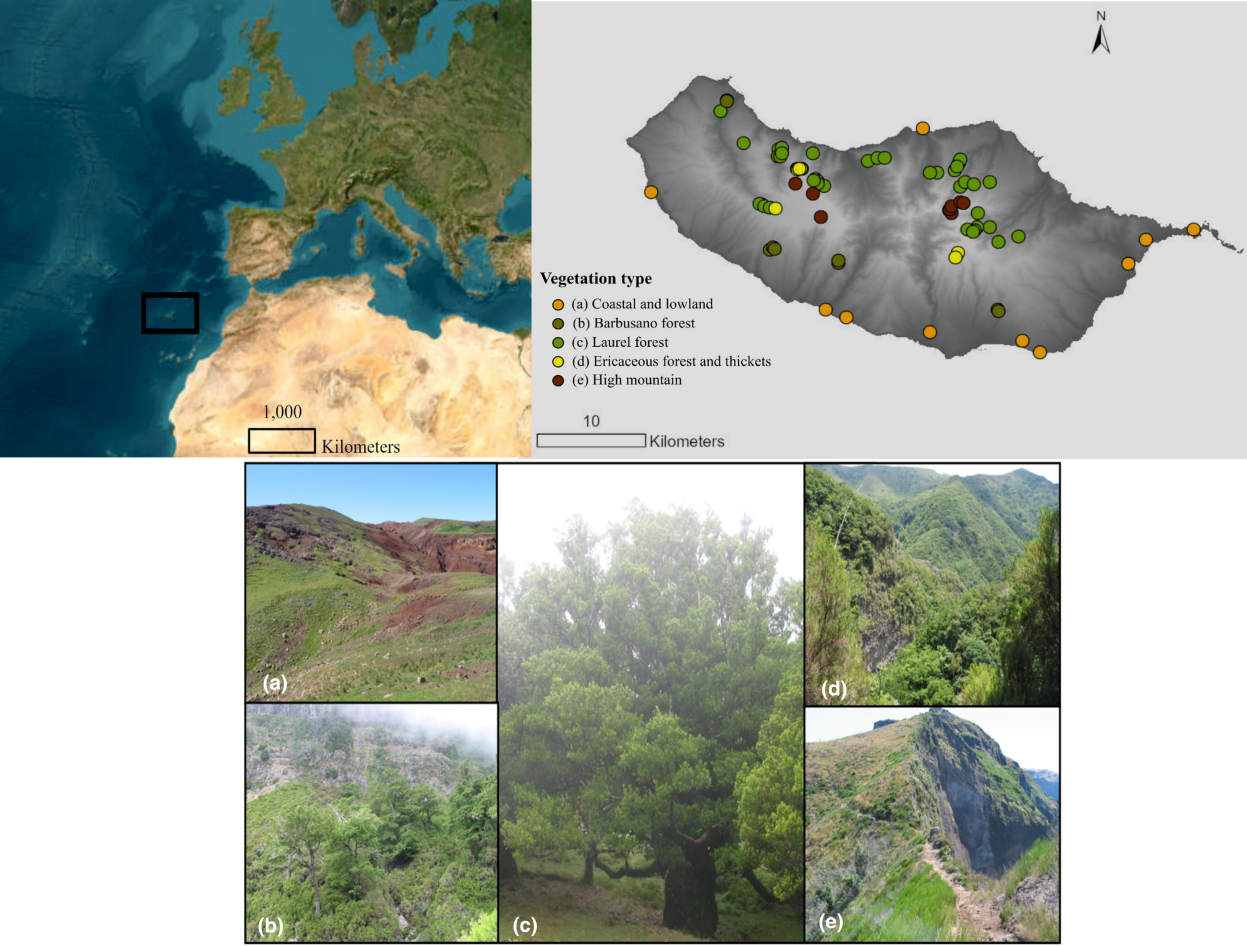
Study Site on Madeira Island. Top left: Location map showing the position of Madeira Island. Top right: Distribution map of 80 plots along the altitudinal gradient across five main vegetation types on Madeira Island. Bottom: Photographs illustrating each vegetation type: (a) Coastal and lowland vegetation; (b) Barbusano forest; (c) Laurel forest; (d) Ericaceous forest and thickets; and (e) High mountain vegetation.

Our study integrates plot‐level data, high‐resolution digital elevation models, trait databases, and genus‐level phylogenies across the entire elevational gradient in Madeira (see Figure 1). Through this comprehensive approach, we seek to address specific questions and test hypotheses as follows:

1. How does relationship among TD, FD, and PD of bryophytes vary along an elevational gradient for both the α‐ and β‐components? Given that competitive exclusion might not exert a significant influence during bryophyte community assembly (Ah‐Peng et al., 2014; Henriques et al., 2017; Patiño & Vanderpoorten, 2021), we anticipate a strong positive relationship among TD, FD, and PD (H1a). However, under more severe conditions, which are likely encountered at the extremes of the elevation gradient or under stronger anthropogenic disturbance regimes, we may either observe no correlations or even a negative relationship among these facets of biodiversity (H1b).
2. How do TD, FD, and PD vary across elevations and among the main bryophyte lineages? Following Hernández‐Hernández et al. (2017), we hypothesize that α‐TD, α‐FD, and α‐PD will exhibit a mid‐elevation peak for both mosses and liverworts (H2a). However, we expect an opposite pattern in growth‐form groupings that thrive in harsh environments (H2b). Given that a significant proportion of bryophyte species on Madeira Island are uniquely found at mid‐elevations (González‐Mancebo et al., 2013; Sim‐Sim et al., 2014), we predict that β‐facets will exhibit an inverse hump‐shaped pattern, reflecting higher homogenization of lineages and growth forms at middle elevations, particularly within ancient forests.
3. What are the drivers of α‐ and β‐TD, FD, and PD of bryophytes? Climate is widely recognized as a primary determinant of bryophyte species distribution, community assembly, and the habitats they occupy (Henriques et al., 2016; Medina et al., 2013; Mouton et al., 2023). However, considering that human‐mediated disturbances can override local climatic factors in controlling spatial patterns of multifaceted plant diversity in montane regions (Liang et al., 2023), we hypothesize that both climatic and anthropogenic factors will play significant roles in shaping both α‐ and β‐patterns of TD, FD, and PD in bryophyte communities (H3).

## 2 MATERIALS AND METHODS

### 2.1 Study area

The present study was conducted in Madeira, the main island of the Madeira archipelago (Figure 1), located in the Northeastern Atlantic Ocean. Northern slopes of Madeira are characterized by steep terrain and receive elevated levels of precipitation in comparison to their southern counterparts. On the northern slopes, the mean annual precipitation nearly reaches 3000 mm. In contrast, areas on the southern slopes, like Funchal and Ponta do Sol, experience significantly lower annual precipitation levels of 513 and 583 mm, respectively (Cruz et al., 2010; Prada et al., 2009). Furthermore, the coastal regions of Madeira display varying annual mean temperatures. The southern slopes generally boast higher temperatures, averaging 19.4°C, in contrast to the northern slopes, which maintain an average temperature of 15.2°C (Cruz et al., 2010; Prada et al., 2009).

In Madeira, five main zonal ecosystems can be identified (Capelo et al., 2005; Sim‐Sim et al., 2014). Coastal and lowland areas [0–300 m a.s.l. in the south; 0–50 (80) m in the north] are characterized by shrubby vegetation primarily dominated by the endemic Madeira wild olive tree, *Olea maderensis* (Lowe) Rivas Mart. & del Arco. This habitat is known for its arid to semi‐arid conditions. Mesic areas (300–800 m in the south; 50–300 m in the north) are associated with the “Barbusano” tree forest, predominantly composed of *Apollonias barbujana* (Cav.) Bornm. This forest exhibits distinctive thermophilic and clearly Mediterranean attributes, marked by transition patches to the laurel forest (Capelo et al., 2005; Sim‐Sim et al., 2014). The laurel forest (800–1450 m in the south; 300–1400 m in the north) consistently experiences high air humidity and provides stable moisture conditions (Sim‐Sim et al., 2014). Despite facing threats such as biological invasions, fires, and climate change (Figueira et al., 2013; Sim‐Sim et al., 2014), the laurel forest remains remarkably well preserved. The ericaceous forest and thickets are part of the high‐altitude tree heath forests (1450–1650 m in the south; 1400–1650 m in the north). These habitats thrive on steep, sunny, and nutrient‐poor exposed slopes (Capelo et al., 2005; Sim‐Sim et al., 2014). In high mountain habitats, above 1650 m, the vegetation is dominated by rupicolous herbaceous and shrub communities. Both ericaceous forest and high mountain habitats face threats such as landslides, fires, and climate warming (Cruz et al., 2010; Sim‐Sim et al., 2014).

### 2.2 Bryophytes from Madeira Island as a study case

Madeira is home to approximately 546 taxa, comprising 6 hornworts, 175 liverworts, and 365 mosses. This accounts for roughly 29% of the total bryophyte species richness in Europe (Hodgetts & Lockhart, 2020). In Madeira, a clear spatial pattern in bryophyte species richness is evident, with the highest level of diversity concentrated in the laurel forest, as opposed to coastal and lowland areas (Boch et al., 2019; Sim‐Sim et al., 2011, 2014). The Madeiran laurel forest hosts 39% of all endemic bryophyte species in Macaronesia (Sim‐Sim et al., 2014). Notably, around 20% of the entire bryophyte diversity in Madeira has been included in Red Lists for the archipelago (Hodgetts et al., 2019; Sim‐Sim et al., 2014).

Besides climate change, various threats must be considered for bryophyte communities within each zonal ecosystem in Madeira. The main threats to bryophytes in coastal/lowland and high mountain habitats include habitat destruction, degradation resulting from increased recreational and tourist activities, and wildfires (Sim‐Sim et al., 2014). Mesic habitats and the laurel forest are primarily threatened by the impact of tourism and biological invasions (Hodgetts et al., 2019; Sim‐Sim et al., 2014). Historically, the destruction of vascular plant vegetation through wildfires and grazing has been one of the primary threats affecting the ericaceous forest and thickets (Sérgio et al., 2008).

Bryophytes were categorized into the two major lineages of mosses and liverworts. Since the number of hornworts was limited to one species, this lineage was combined with liverworts for the subsequent analyses. Furthermore, mosses and liverworts were classified based on two different growth forms: acrocarpous or pleurocarpous mosses, and thalloid or leafy liverworts, respectively. These four groups exhibit distinct tolerances to drought. Acrocarpous mosses, known for their desiccation‐tolerant gametophytes, thrive in sunnier, drier, and xeric habitats (Kürschner et al., 1999). In contrast, pleurocarpous mosses are better adapted to shady environments and can condense water vapors from atmospheric humidity and fog (Kürschner et al., 1999). Reflecting these distinct drought tolerances, acrocarpous mosses typically feature a central conducting strand, and their leaf surfaces often have protuberances that aid in water transport and storage. In contrast, pleurocarpous species tend to remain close to the substrate, making a central axis less necessary (Kürschner et al., 1999; Shaw & Goffinet, 2000). On a global scale, thalloid liverworts encompass genera that infrequently produce specialized asexual diaspores. Instead, they rely on relatively large spores that can more effectively develop under favorable conditions (Bischler‐Causse et al., 2005). In contrast, leafy liverworts are generally more susceptible to drought and possess the ability to produce both specialized asexual diaspores and comparatively smaller spores (Collart et al., 2021).

### 2.3 Bryophyte sampling

Bryophyte sampling took place on Madeira Island between 2014 and 2018, using 0.5 m × 0.5 m plots distributed along the whole elevation gradient covering the five major zonal ecosystems. Plots were randomly located at elevations ranging from 100 to 1750 m a.s.l, with 10 plots established every 200 m. A total of 80 plots (as shown in Figure 1 and Table S1) were examined on slopes with gradients ranging from 2% to 60%. Site accessibility and the maintenance of a minimum distance of 500 m from roads and paths were considered during plot selection. Within each plot of 0.5 m × 0.5 m, representative samples of all bryophytes were collected, and information about elevation, vegetation type, and main human‐induced disturbances was recorded. All bryophyte samples were identified at the level of species. Nomenclature follows Hanusch et al. (2020) and Hodgetts et al. (2020) for *Epipterygium atlanticum*. Samples are deposited at the herbarium LISU.

### 2.4 Species trait data

Trait data were obtained from three primary sources: BET (van Zuijlen et al., 2023), BRYOTRAIT‐AZO (https://islandlab.uac.pt/software/ver.php?id=26), and BRYOATT datasets (Hill et al., 2007). These were supplemented with bibliographic references (see Table S2) and our own observations from Madeiran specimens. Eleven life‐history traits were taken into account, including capsule position in relation to perichaetium (exserted; not exserted; partly exserted); generation length (following Bergamini et al., 2019); laminal/median cells ornamentation (mamillose; papillose; smooth); leaf length; life forms (cushions; dendroids; fans; mats; pendants; turfs; wefts) defined according to Bates (1998); life strategy (annual; colonist; long‐lived shuttle; perennial; short‐lived shuttle) defined according to During (1992); operculum (presence/absence); peristome (presence/absence); sexual condition (dioicous; dioicous and monoicous; monoicous); shoot or thallus length; and spore size (Table S2).

### 2.5 Species phylogeny

Phylogenetic information was derived from the chronograms of the Moss and Liverwort Tree of Life, which are based on three and eight genes from all genomic compartments. In the case of mosses, 539 of the 845 genera were sequenced, while for liverworts, 303 genera representing 84% of the total extant generic diversity were also included in the sequencing (Laenen et al., 2014). The phylogeny was built with one representative species per genus. Consequently, all congeneric species included in the distribution database were integrated into the genus‐level phylogeny (Table S3). Nine species from genera that were not included in the study by Laenen et al. (2014) had to be, therefore, excluded from the dataset used in the present study to estimate PD metrics described below (Table S4). To account for phylogenetic uncertainty, we generated and analyzed 100 trees with randomly resolved relationships among congeneric species, following the approach proposed by Collart et al. (2021).

### 2.6 Explanatory variables

The present study investigated how eight explanatory variables, encompassing biotic, abiotic, and anthropogenic factors, influence TD, FD, and PD of bryophytes along the elevational gradient in Madeira. These variables, evaluated at each sampling plot, include one biotic factor (vegetation type), five abiotic factors (elevation, geological formation, mean annual precipitation, mean annual temperature, and slope), and two anthropogenic factors (distance to a main road and land use). Vegetation types were determined in the field and categorized according to Capelo et al. (2005), considering: coastal and lowland, barbusano forest, laurel forest, ericaceous forest and thickets, and high mountain. Geological formations were identified using a map with approximately 500‐m resolution provided by the Regional Directorate for Territorial Ordering and Environment (DROTA; https://www.madeira.gov.pt/sraac). Three main geological formations were designated: upper volcanic complex (CVS ~1.8–0.007 Ma), intermediate volcanic complex (CVM ~5.57–1.8 Ma), and recent sedimentary deposits. Mean annual precipitation and temperature were recorded at a 30‐arc‐second (~1 km) resolution from CHELSA v. 2.1 (Karger et al., 2017). Distance to regional roads was obtained at approximately 10‐m resolution from the Forest GIS database (www.forest‐gis.com). Land use data at approximately 100‐m resolution and slope data at about 10‐m resolution were sourced from the European Union's Copernicus Land Monitoring Service for the year 2018 (https://land.copernicus.eu/). Seven land use categories were identified, including discontinuous urban fabric, heathland, sparsely vegetated areas, broadleaved forest, land predominantly used for agriculture with significant natural vegetation, transitional woodland–shrub, and mixed forest. All variable values were extracted for the plot location using ArcGIS 10.7 (ESRI, 2021) and subsequently standardized (standard deviation = 1 and mean = 0).

### 2.7 Estimating taxonomic, functional, and phylogenetic diversity

We generated two comprehensive presence–absence taxa data matrices for mosses and liverworts. Subsequently, we categorized them based on their growth forms: acrocarpous and pleurocarpous for mosses, and thalloid and leafy for liverworts. These groupings resulted in six distinct presence–absence datasets, which were used to calculate both α‐ and β‐components of TD, FD, and PD using the “BAT” R package (Cardoso et al., 2014). The β‐component was measured using the Jaccard's index (β‐total). For PD estimation, we loaded the phylogenetic trees (“phylo” object) using the “ape” R package (Paradis et al., 2004) and computed the mean PD across values derived from 100 phylogenetic trees. We first generated distance trait matrices using the Gower distance coefficient with the *gowdis* function from the “FD” R package (Laliberté et al., 2014). This method is designed to compute Gower dissimilarity, accommodating various trait types, including qualitative and quantitative traits. Subsequently, functional dendrograms were generated from these matrices using the *hclust* function in R.

### 2.8 Statistical analyses

To investigate the completeness of sampling in relation to differences in sample size (i.e., number of plots) of each main bryophyte and growth‐form grouping, we compared observed species richness with predicted species richness using the *specpool* function of the “vegan” R package (Oksanen et al., 2022). Namely, species richness values were predicted by computing Chao, Jackknife 1, and Jackknife 2 estimators. These estimators have consistently displayed robust performance when compared to several other methods for measuring species richness (Hortal et al., 2006). The combination of these estimators proved valuable in assessing the degree to which our sampling approach underestimated bryophyte species richness in each grouping.

To explore the relationship among the three facets of α‐ and β‐components, respectively, within each bryophyte grouping (H1a and H1b), Pearson's correlation was assessed using the *rcorr* function of the “Hmisc” R package (Harrell & Dupont, 2023). Additionally, to investigate elevational trends in TD, FD, and PD of α‐ and β‐bryophyte components (H2a and H2b), linear models (LM) were employed (R Core Team, 2022). For each α‐ and β‐TD, FD, and PD metric, three distribution types, including linear, quadratic, and logarithmic distributions, were fitted. The best‐performing LM was selected based on the Akaike information criterion corrected for small sample sizes (AICc, Burnham & Anderson, 2002). A null model with 100 replicates, represented by an LM with an intercept term only, was also implemented to test the potential absence of a relationship.

To determine which explanatory variables best account for the patterns of α‐ and β‐TD, FD, and PD within each of the six bryophyte groupings (H3), we first computed Pearson correlation coefficients among each pair of explanatory variables. To avoid multicollinearity and variable redundancy, we retained only one explanatory variable from any pair with a correlation coefficient > 0.85, as suggested by Dormann et al. (2013). Given the strong correlation between annual mean temperature and the minimum coefficient of elevation, we opted to retain temperature (Table S5).

Following multicollinearity analyses, we used Linear Mixed‐Effects Models (LMMs) to explore the relationship between α‐diversity matrices and the resulting subset of explanatory variables, using the *lmer* function in the “lme4” R package (Bates et al., 2015). Each α‐diversity metric (across all three facets) was treated as a response variable, with eight explanatory variables designated as fixed predictors. The slope (i.e., north vs. south) was considered as a random effect. To identify the best‐fitting model, we tested all possible models using the *glmulti* function in the “glmulti” R package (Calcagno & de Mazancourt, 2010). Model selection was based on AICc. Models with a ΔAICc value of less than 2 units from the best model were deemed plausible (Burnham & Anderson, 2002). A variable that appears in many models with large weights will receive a high importance value. The model that encompassed all variables with substantial weights (above a 40% threshold) was identified as the best‐fitting model. The direction of selected variables was assessed based on the sign of standardized coefficients. Then, we calculated the proportion of variance explained by the variables included in the best‐fitting model and their statistical significance, using the *partR2* function in the “partR2” R package (Stoffel et al., 2021) and the *anova* function in the “car” R package (Fox et al., 2019), respectively.

Generalized dissimilarity modeling (GDM), a non‐linear statistical regression technique, was then used to model the three facets of β‐diversity and explored potential relationships with ecological predictors. Specifically, GDM was used for assessing the relationship between the selected eight explanatory variables and local variation in taxonomic, community composition, using the *formatsitepair* function of the “gdm” R package (Fitzpatrick et al., 2021). Given the unique geographical and environmental characteristics of Madeira, introducing a slope‐based predictor was crucial to account for the inherent spatial structure in the data. The significant variations in topography and climatic conditions between the northern and southern slopes of the island can directly influence the distribution and composition of biological communities. Therefore, including slope as a control variable allows us to more accurately capture and analyze these environmental disparities. Specifically, pairs of plots from the same slope (north vs. north or south vs. south) were assigned a value of 0, while pairs from different faces (south vs. north) were assigned a value of 1. To refine the model and control for significant variables, a stepwise backward variable elimination process was implemented using the *gdm.varImp* function of the “gdm” R package. This procedure continued until all variables had a *p* value <.05. All analyses were performed in R version 4.1.3 (R Core Team, 2022).

## 3 RESULTS

### 3.1 Species richness and sampling completeness

A total of 184 bryophyte taxa from the 80 studied plots were identified, composing of 101 moss, 80 liverwort, and one hornwort species. These taxa were further categorized into 62 acrocarpous and 40 moss pleurocarpous taxa, as well as 24 thalloid and 58 leafy liverwort taxa (Table S2). This sampling effort accounted for approximately 33% of the overall bryophyte flora of Madeira. Regarding sampling completeness, our sampling reached high levels for the two main taxonomic groups, varying from 79.47 ± 2.98 (mosses) to 82.45 ± 1.36 (liverworts) (Table S6). The acrocarpous mosses showed values of 82.12 ± 3.37, and the pleurocarpous mosses exhibited values of 68.74 ± 5.27. The thalloid and leafy liverworts exhibited values of 80.72 ± 10.25 and 84.27 ± 3.05, respectively.

### 3.2 Relationships among diversity facets

**FIGURE 2:**
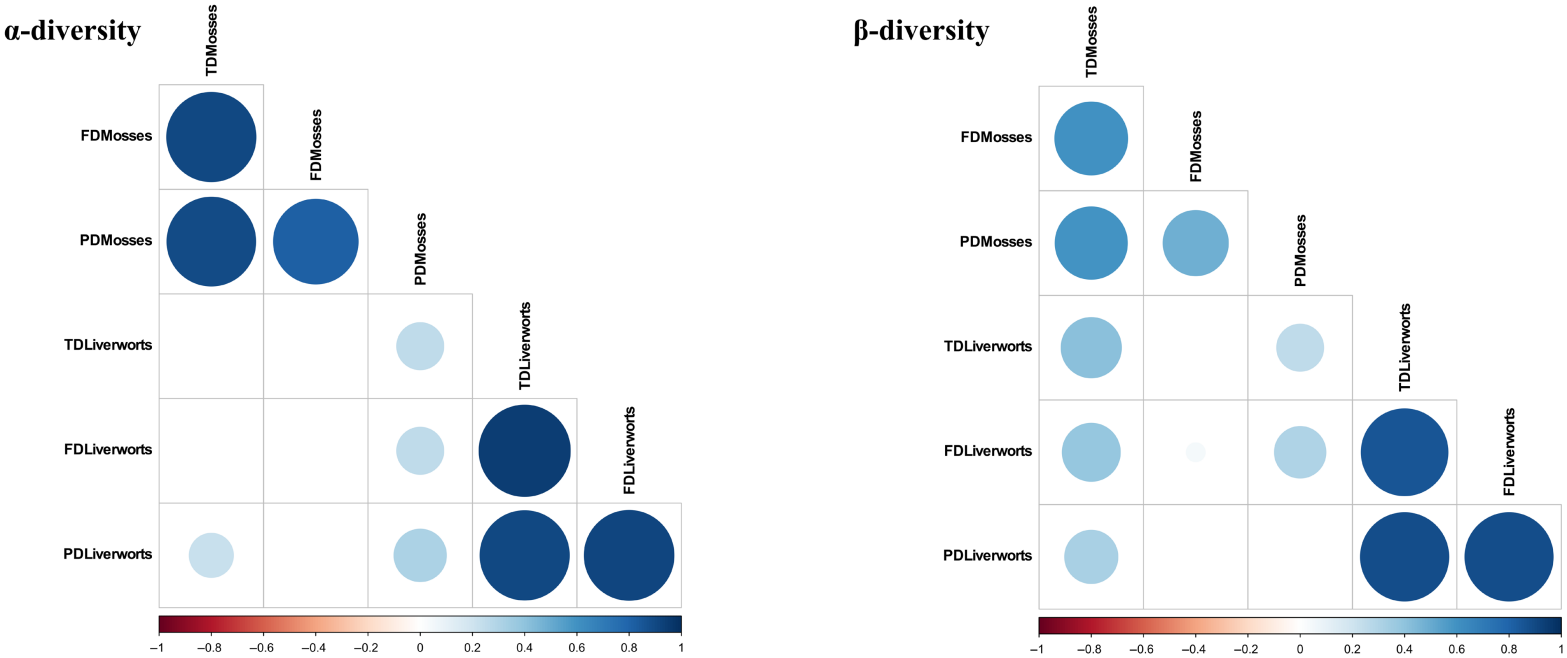
Correlogram of the three facets (FD, functional diversity; PD, phylogenetic diversity; TD, taxonomic diversity) of α‐ and β‐diversities for the two main lineages of bryophytes, mosses, and liverworts, on Madeira Island. Only significant correlations (*p* < .05) are colored. The size of the dots represents the strength of the correlations, with larger circles indicating stronger correlations.

We found a significant positive correlation among the three facets of diversity for both the α‐ and β‐components in mosses and liverworts, respectively (Figure 2). Within growth‐form groupings, we detected fewer significant correlations for β‐diversity than for α‐diversity facets (Figure S1). A significant positive correlation was identified for all facets within acrocarpous mosses, thalloid liverworts, and leafy liverworts (Pearson correlation *r* coefficients ranging between 0.51 and 1.00, Table S7). Furthermore, a positive correlation was noted between β‐PD and β‐FD in pleurocarpous mosses (Figure S1 and Table S8).

### 3.3 Relationship between diversity facets and elevation

**FIGURE 3:**
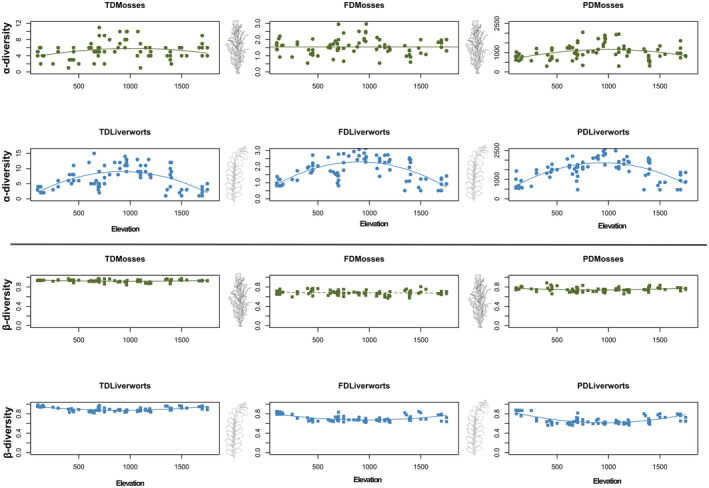
Response plots of the three facets (FD, functional diversity; PD, phylogenetic diversity; TD, taxonomic diversity) of α‐diversities (top panels) and β‐diversities (bottom panels) to the elevational gradient in Madeira Island for mosses and liverworts. Solid lines indicate significant relationship between the three‐facet diversity and elevation while dashed lines indicate non‐significant relationships.

The patterns of α‐TD and α‐PD in mosses and all the facets in liverworts exhibited a significant quadratic relationship with elevation (Figure 3 and Table S9). When examining growth forms, a quadratic distribution was also the best‐fitting model for all three diversity facets of pleurocarpous mosses and leafy liverworts (Figure S2 and Table S9). Many of these lineages and groupings exhibited their peak values of α‐diversity facets at mid‐elevations, spanning from 650 m a.s.l. to approximately 950 m a.s.l. (Figure 3, Figure S2 and Table S9). The three α‐diversity facets in thalloid liverworts, and α‐TD and α‐FD in acrocarpous mosses best fit a shallow, decreasing logarithmic distribution (see Figure S2 and Table S9). These groups exhibited higher diversity values at lower elevations. Notably, there was no discernible relationship between elevation for α‐FD of mosses and α‐PD of acrocarpous mosses (Figure 3 and Figure S2).

The patterns of β‐TD and β‐PD in mosses, as well as all β‐facets of liverworts, exhibited an inverse quadratic relationship with elevation (Figure 3 and Table S9). A similar inverse hump‐shaped relationship with elevation was also observed for β‐TD in both acrocarpous and pleurocarpous mosses, as well as for all facets of leafy liverworts. A logarithmic model was identified as the best‐fitting distribution for β‐PD in acrocarpous mosses, β‐FD and β‐PD of pleurocarpous mosses, along with all three facets of thalloid liverworts (Figure S3 and Table S9). For β‐FD in leafy liverworts, an increasing linear model was the best‐fitting distribution (Figure 3 and Figure S3).

### 3.4 Ecological drivers of alpha and beta diversity

**FIGURE 4:**
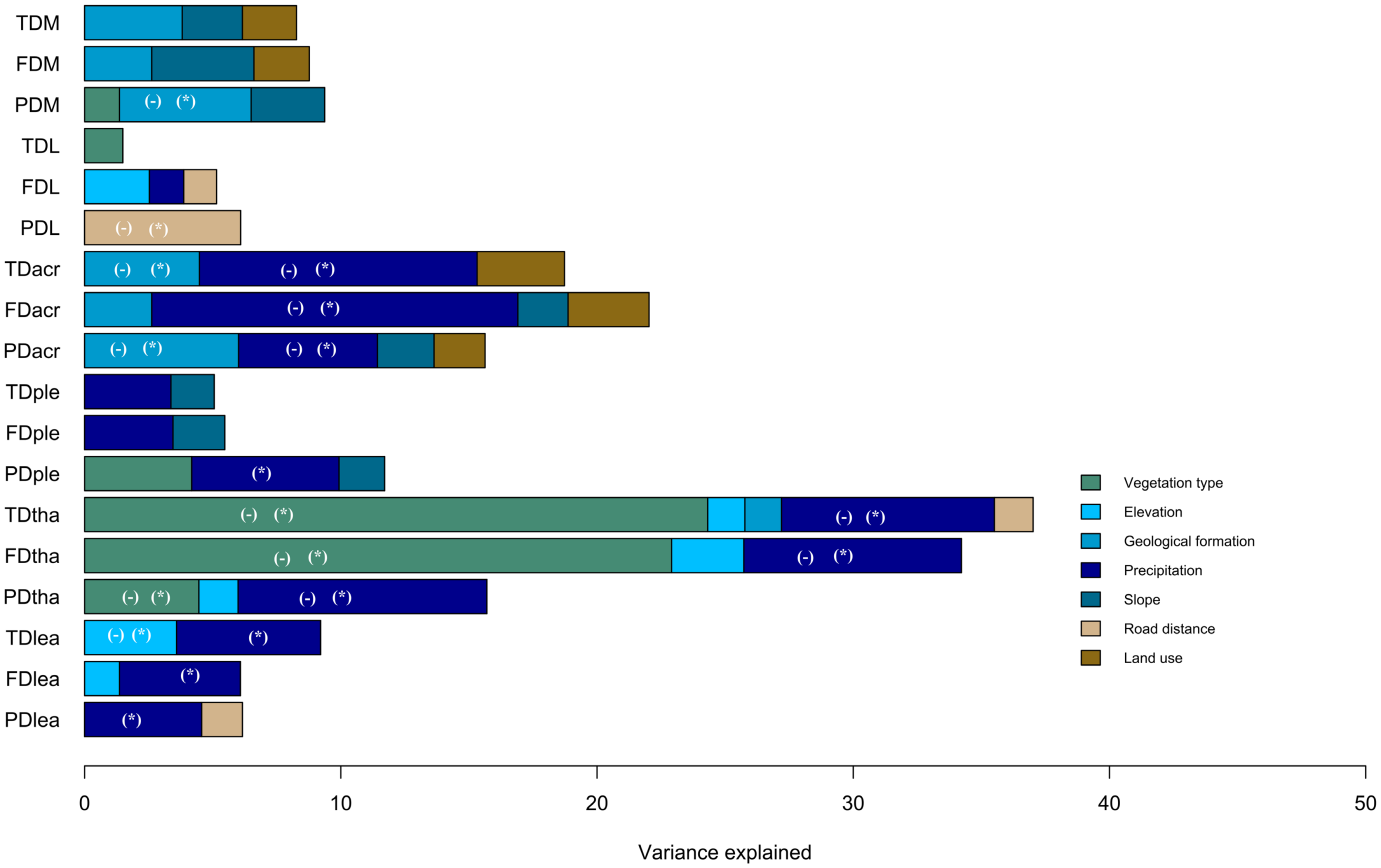
The variance explained, based on linear mixed‐effects models (LMMs), each variable on all the three facets of α‐diversity (FD, functional diversity; PD, phylogenetic diversity; TD, taxonomic diversity) for the six groups (acr, acrocarpous mosses; lea, leafy liverworts; L, liverworts; M, mosses; ple, pleurocarpous mosses; tha, thalloid liverworts). Only the variables with variance explained <1% were presented. The categorical variables were categorized as follows: Geological formation from the oldest (upper volcanic complex) to the most recent (sedimentary deposits). Vegetation types from coastal and lowland to high mountains, and land use from the most artificial (discontinuous urban fabric) to the most natural (mixed forest). (−) Indicates the negative sign of standardized coefficients, *p < .05.

In relation to the α‐diversity component, ecological drivers accounted for varying percentages of the observed variance, ranging from 7% to 50%, depending on the diversity metric (Table S10 and Figure S4). Among the considered variables, precipitation emerged as the most influential, exhibiting a negative effect on all three facets of acrocarpous mosses and thalloid liverworts, while positively affecting all three facets of leafy liverworts and α‐PD of pleurocarpous mosses. The type of vegetation, ranging from coastal lowland to high mountain, also had a negative influence on the three facets of thalloid liverworts (Figure 4, Table S10 and Figure S4). The type of geological formation exhibited a negative effect on the models for α‐PD of mosses, α‐TD, and α‐PD of acrocarpous mosses, with lower values observed on recent sedimentary deposits (see Figure 4, Table S10 and Figure S4). Moreover, proximity to roads and elevation also had a negative effect on the models for α‐PD of liverworts and α‐TD of leafy liverworts, respectively (Figure 4, Table S10 and Figure S4).

**FIGURE 5:**
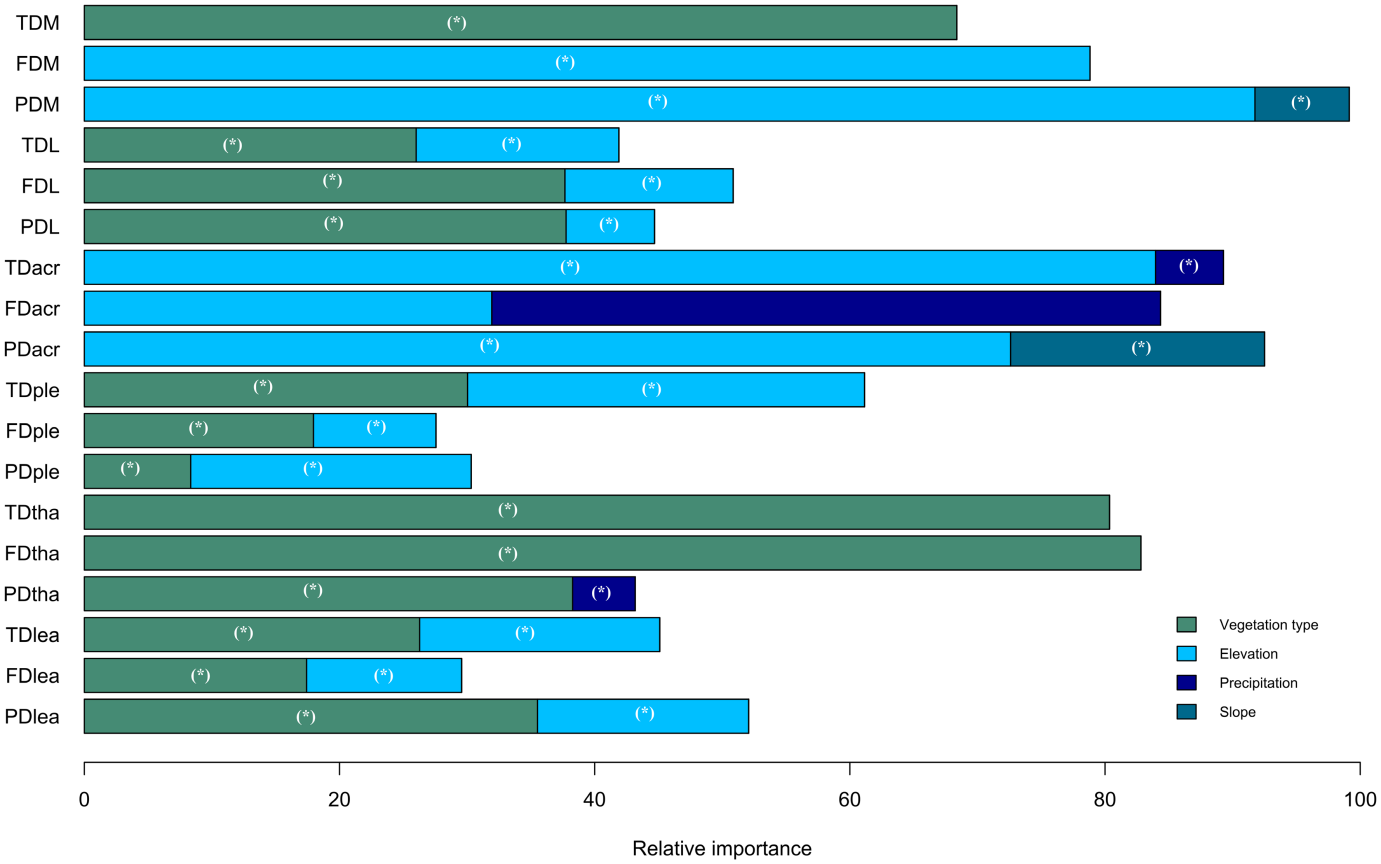
The relative importance, based on generalized dissimilarity modeling (GDM), of each variable (vegetation type, elevation, precipitation, and slope) on all the three facets of β‐diversity (FD, functional diversity; PD, phylogenetic diversity; and TD, taxonomic diversity) for the six groups (acr, acrocarpous mosses; lea, leafy liverworts; L, liverworts; M, mosses; ple, pleurocarpous mosses; tha, thalloid liverworts, *p < .05). Vegetation type was categorized from coastal and lowland to high mountain.

On the β‐diversity component, ecological drivers accounted for 56% of the deviance in β‐PD of leafy liverworts, while explaining less than 50% for the remaining diversity facets (Table S11 and Figure S5). Vegetation type made a crucial contribution to the models for the three β‐diversity facets of liverworts, pleurocarpous mosses, thalloid liverworts, leafy liverworts, and β‐TD of mosses (Figure 5, Table S11 and Figure S5). Elevation was the primary explanatory variable for β‐FD and β‐PD of mosses, as well as β‐TD and β‐PD of acrocarpous mosses and pleurocarpous mosses (Figure 5, Table S11 and Figure S5). It also made a significant contribution to the models for the three β‐diversity facets of liverworts and leafy liverworts, as well as β‐FD of pleurocarpous mosses. Precipitation emerged as the second influential variable in the models for β‐TD of acrocarpous mosses, and β‐PD of thalloid liverworts. Additionally, slope made a greater contribution to the models for β‐PD of mosses, and β‐PD of acrocarpous mosses (Figure 5, Table S11 and Figure S5).

## 4 DISCUSSION

Our study provides an integrative local‐scale assessment of patterns within both α‐ and β‐components of multiple bryophyte diversity facets and their underlying drivers. We showed that an increase in bryophyte TD (i.e., species richness) tends to both coincide with and predict an increase in PD and FD. Indeed, the interplay between PD and FD within a community has been demonstrated to hold a pivotal role in various ecological processes. These processes include influencing responses to environmental gradients (Arnan et al., 2015; Wang et al., 2019), mediating species interactions and community assembly (HilleRisLambers et al., 2012; Pavoine & Bonsall, 2011), and including other alternative filters that define the species pool and the subsequent level of niche partitioning (Perronne et al., 2017; Swenson, 2013).

### 4.1 Relationship among taxonomic, functional, and phylogenetic diversity patterns

In line with our first hypothesis (H1a), the trends observed in the α‐ and β‐components of the TD, FD, and PD facets within both mosses and liverworts confirm a consistently positive correlation across Madeira Island. We have identified a robust and positive relationship among TD, FD, and PD, not only within each main bryophyte lineage but also across each growth‐form grouping. Notably, previous research on α‐ and β‐diversity patterns has consistently documented positive associations between TD and both PD and FD across various organisms, spatial scales, and geographic regions. Examples include pteridophytes (Kluge & Kessler, 2010), mammals (Kohli et al., 2021; Safi et al., 2011), ants (Arnan et al., 2016), birds (Devictor et al., 2010), and fish (Teichert et al., 2018). Furthermore, the positive correlation between FD and PD represents a trend that, while sometimes unexpected or limited (Mazel et al., 2018), has been increasingly documented (Cadotte et al., 2019). This supports the notion that closely related species tend to exhibit greater similarity in functional traits (Blomberg et al., 2003).

We observed no significant or negative correlations between α‐ and β‐diversity facets of acrocarpous mosses and thalloid liverworts when compared with pleurocarpous mosses and leafy liverworts, respectively. This outcome supports our expectation H1b, suggesting that under more challenging conditions—likely encountered at the extremes of the elevation gradient or in the presence of more intense anthropogenic disturbance regimes—patterns of uncorrelated or divergent diversity metrics become more apparent (Arnan et al., 2018). Indeed, acrocarpous mosses and complex thalloid liverworts typically exhibit broader ecological requirements, thriving in sunnier, drier, and more xeric habitats (Kürschner et al., 1999; Proctor et al., 2007). In contrast, pleurocarpous mosses and leafy liverworts are globally better adapted to shaded and humid environments, such as those found in laurel and ericaceous forests, where mist precipitation enhances water availability throughout all seasons (Lloret & González‐Mancebo, 2011; Patiño et al., 2009).

The negative or absence of correlation between these two types of growth forms suggests that in areas with high levels of TD, FD, and PD among acrocarpous mosses and thalloid liverworts, where conditions are more challenging due to natural or anthropogenic disturbances, these growth forms tend to outcompete pleurocarpous mosses and leafy liverworts. This carries several implications. Firstly, it could intensify competition among species, given their functional and phylogenetic dissimilarities. Secondly, this competitive disadvantage and lower fitness under harsher conditions might result in reduced population sizes for pleurocarpous mosses and leafy liverworts, detrimentally impacting their long‐term persistence (Escobar‐Luján et al., 2022; Hedenäs, 2001). While metrics such as PD and FD have significantly contributed to our understanding of community assembly (Cadotte et al., 2017; Kraft & Ackerly, 2010), each approach is accompanied by inherent assumptions and limitations that impact their practicality and interpretability (Cadotte et al., 2013; Gerhold et al., 2015). To address this challenge, the proposal of implementing long‐term experimental studies has been suggested (Cadotte et al., 2019), an approach particularly essential in oceanic archipelagos like Madeira (Borges et al., 2018).

### 4.2 Elevation patterns of diversity facets

Our analysis of the spatial patterns in the α‐component of TD, FD, and PD of bryophytes in Madeira unveiled a prevalent hump‐shaped relationship with elevation, aligning with our hypothesis H2a. This hump‐shaped pattern was also evident for both α‐TD and α‐PD in mosses overall, as well as for all three facets of liverworts, leafy liverworts, and pleurocarpous mosses. Comparable hump‐shaped patterns with similar peaks emerged when assessing α‐TD of both bryophytes in general (e.g., Boch et al., 2019; Grau et al., 2007; Iskandar et al., 2020) and other plant groups (e.g., Dani et al., 2023; Grytnes & Vetaas, 2002; Nogués‐Bravo et al., 2008). In line with our findings, Rahbek (2005) estimated that about 50% of the documented elevational patterns in species richness displayed a unimodal shape. Although less explored, α‐FD and α‐PD have also shown a similar quadratic relationship with elevation and factors related to it (Ding et al., 2021; Kohli et al., 2021).

The observed hump‐shaped elevational pattern in this study is likely attributable to the mid‐domain effect (Colwell & Hurtt, 1994), indicating more favorable environmental conditions at intermediate elevations (Boch et al., 2019; Currie & Kerr, 2008; Grytnes & Vetaas, 2002; McCain & Grytnes, 2010). These conditions are characterized by consistently high humidity levels year‐round, minimal temperature fluctuations, and a rise in microhabitat complexity—all pivotal attributes defining the Madeiran laurel forest (Boch et al., 2019; Sim‐Sim et al., 2014). The significance of altitudinal cloud belts in controlling the distribution of bryophyte taxa, particularly those highly dependent on humidity conditions within the Macaronesian laurel forest, has frequently been emphasized (Henriques et al., 2017; Hernández‐Hernández et al., 2017; Patiño et al., 2010).

Previous evidence has shown that the elevational gradient of diversity facets can take several alternative shapes, such as increasing, declining, multimodal, or showing no association (Montaño‐Centellas et al., 2020; Rahbek, 1995, 1997; Shooner et al., 2018). In line with our expectation H2b, growth‐form groupings that thrive in harsh environments displayed different patterns across elevations for multiple diversity facets. For instance, a decreasing non‐linear pattern was observed for α‐TD and α‐FD in acrocarpous mosses and all three facets of thalloid liverworts. Indeed, acrocarpous mosses and complex thalloid liverworts, globally recognized as desiccation‐tolerant growth forms (Kürschner et al., 1999), tend to be overrepresented in the lowlands of Madeira. The relatively adverse climate conditions such as higher temperature regimes, intense solar radiation, and low atmospheric humidity at lower altitudes would limit the presence of certain growth forms. Simultaneously, the interplay of interspecific competition and abiotic filtering could lead to the reduction and homogenization of biological communities at lower altitudes (Körner, 2007).

In contrast to α‐diversity metrics, our findings identified that the prevailing pattern observed in the β‐components of bryophyte diversity across the elevational gradient was an inverse hump‐shaped or a U‐shaped relationship. Specifically, β‐TD and β‐PD of mosses, all the three facets of liverworts and leafy liverworts, as well as β‐TD of acrocarpous and pleurocarpous mosses, all exhibited a U‐shaped pattern with elevation. This result indicates lower turnover at mid‐elevation, supporting the observation that biotic communities in more extreme elevations or arid areas exhibit greater heterogeneity in community composition compared to mid‐elevations or humid regions, where co‐occurring species are more ubiquitous, phylogenetically related, and functionally similar (Ding et al., 2021). In warmer and drier regions, there tends to be a lower redundancy in species, with a higher presence of functionally and phylogenetically distinct species, indicating potential adaptations to the prevailing conditions (do Nascimento et al., 2018). Such a pattern might be ultimately associated with niche conservatism (Wiens & Graham, 2005), in which lower phylogenetic and functional distance can be interpreted as a surrogate of lower interspecific dissimilarity at multiple niche dimensions (Crisp et al., 2009; Swenson, 2013). Therefore, our results suggest a higher degree of homogenization for bryophyte communities and growth‐form groupings at mid‐elevation within the Madeiran laurel forest, where a significant proportion of bryophyte species exclusive to this ecosystem are also widely distributed (Patiño et al., 2018; Sim‐Sim et al., 2014).

### 4.3 Ecological drivers of multiple biodiversity facets

The constrained variability (approximately <50%) revealed by LMMs and GDMs suggests that factors beyond those considered in our study may influence biodiversity gradients at the island level, or that the variables employed in our study may not fully capture biodiversity patterns in bryophytes. Despite this potential limitation, several general conclusions can be inferred from our findings. In partial agreement with our initial hypothesis H3, which posited that both climatic and anthropogenic factors would significantly impact α‐ and β‐TD, PD, and FD of bryophytes, our results indicated that precipitation and the dominant vegetation type were the most influential factors in shaping multiple facets of bryophyte biodiversity on Madeira. This result aligns with previous evidence showing that vegetation structure and climate play a key role in explaining patterns of biodiversity facets at both global and regional scales (Feng et al., 2020; Prescott et al., 2016; Rurangwa et al., 2021), including bryophytes (Chen et al., 2017; Collart et al., 2024; Ilić et al., 2023; Mežaka et al., 2012; Shen et al., 2023). At the local scale, environmental filtering associated with elevation, climate, and habitat structure played a more important role in shaping α‐ and β‐components of TD, FD, and PD in bryophytes (Araújo et al., 2022; Chen et al., 2017) and spermatophytes (Ben Saadi et al., 2022; Culmsee & Leuschner, 2013). On Madeira Island, both precipitation and vegetation are strongly determined by elevation and slope at the local scale (Capelo et al., 2005; Sim‐Sim et al., 2014), a situation that reinforces the role of these ecological drivers on the three facets of α‐ and β‐diversities and community assembly.

Remarkably, precipitation had a negative effect on all three alpha‐facets of acrocarpous mosses as well as thalloid liverworts. Furthermore, the negative influence of vegetation on these growth‐form groupings can be interpreted as stemming from the inhibitory effects of shade, acting as a coarse proxy for fine‐scale habitat heterogeneity. This interpretation is supported by the highly contrasting structures, compositions, and microecological gradients of the main vegetation types considered. As previously noted, this finding aligns with the observation that the majority of acrocarpous moss and thalloid liverwort species tend to thrive in harsher habitats characterized by limited precipitation levels. The negative impacts observed in more humid and shaded habitats for certain functional groupings stand in contrast to the favorability hypothesis (Fischer, 1960), which suggests lower diversity in harsh environments. Additionally, these findings deviate from the positive effects of precipitation observed in the present study on other growth‐form groupings and plant groups, as previously documented (e.g., Manish et al., 2017; Mokany et al., 2022; Sabatini et al., 2022).

Proximity to roads is the only anthropogenic factor with a significant effect on some facets of α‐ and β‐diversities in bryophytes on Madeira. Specifically, proximity to roads had a negative impact on the α‐PD of liverworts. Consistently, anthropogenic factors might play a secondary role in α‐ and β‐diversities and composition of bryophytes along an altitudinal gradient as previously shown in the Atlantic Forest in Brazil (Araújo et al., 2022). Our results hence support that α‐ and β‐TD, PD, and FD of bryophytes on Madeira Island are more strongly influenced by climatic factors and the predominant type of vegetation than by the considered anthropogenic factors, such as distance to roads and land use. However, caution should be exercised when interpreting these results, as they might also point to limitations in the variables considered. Future research stands to gain from the increasing accessibility of trait and phylogenetic data (Vasconcelos, 2023) and higher‐resolution environmental factors from additional Macaronesian archipelagos (Borges et al., 2018; Collart et al., 2024; Patiño et al., 2023). Such data would facilitate the evaluation of the universality of plant community diversity patterns across environmental gradients and provide insights into how community assembly and biodiversity might respond to long‐term human pressures on oceanic islands (Whittaker et al., 2008).

## AUTHOR CONTRIBUTIONS

**Anabela Martins:** Conceptualization (equal); formal analysis (equal); methodology (equal); writing – original draft (lead); writing – review and editing (lead). **Flavien Collart:** Formal analysis (equal); writing – original draft (supporting); writing – review and editing (supporting). **Manuela Sim‐Sim:** Conceptualization (equal); data curation (lead); funding acquisition (lead); supervision (equal); writing – original draft (equal); writing – review and editing (equal). **Jairo Patiño:** Conceptualization (equal); formal analysis (equal); supervision (equal); writing – original draft (equal); writing – review and editing (equal).

## FUNDING INFORMATION

Fundação para a Ciência e a Tecnologia, grant/award number: 2020.06119.BD and PTDC/AGR‐FOR/3427/2014; Spanish Ministry of Science and Innovation, grant/award number: RYC‐2016‐20506 and ASTERALIEN—PID2019‐110538GA‐I00.

## CONFLICT OF INTEREST STATEMENT

The authors declare no conflict of interest.

## Supporting information


Appendix S1:

## Data Availability

The data matrix of species that support the findings of this work is available at https://figshare.com. https://doi.org/10.6084/m9.figshare.24298051.v1.
