# Supplementary material for: Ecological drivers of taxonomic, functional, and phylogenetic diversity of bryophytes in an oceanic island
Source: Ecol Evol. 2024 Jul 24;14(7):e70023. doi: 10.1002/ece3.70023 (PMC11269207; doi:10.1002/ece3.70023)
Supplement: Supplementary file 1 — Appendix S1: [file ECE3-14-e70023-s001.docx]

**Ecological drivers of taxonomic, functional and phylogenetic diversity of bryophytes in an oceanic island**

**AppendixS1-**Supplementary Tables and Figures

Table S1. List of plots with information on location (northing, easting), elevation, slopes (northern versus southern) and vegetation type.

| **Plot** | **Northing** | **Easting** | **Altitude** | **Slope** | **Vegetation type** |
| --- | --- | --- | --- | --- | --- |
| 1 | 3624152 | 340604 | 100 | Southern | Coastal and lowland |
| 2 | 3623203 | 336251 | 100 | Southern | Coastal and lowland |
| 3 | 3620897 | 334573 | 104 | Southern | Coastal and lowland |
| 4 | 3612579 | 326467 | 130 | Southern | Coastal and lowland |
| 5 | 3613625 | 324800 | 250 | Southern | Coastal and lowland |
| 6 | 3614468 | 316290 | 150 | Southern | Coastal and lowland |
| 7 | 3615874 | 308578 | 160 | Southern | Coastal and lowland |
| 8 | 3616608 | 306639 | 130 | Southern | Coastal and lowland |
| 9 | 3627687 | 290588 | 120 | Southern | Coastal and lowland |
| 10 | 3633687 | 315668 | 100 | Northern | Coastal and lowland |
| 11 | 3631717 | 302339 | 440 | Northern | Laurel forest |
| 12 | 3631869 | 302597 | 450 | Northern | Laurel forest |
| 13 | 3636361 | 297530 | 430 | Northern | Laurel forest |
| 14 | 3635331 | 296944 | 410 | Northern | Laurel forest |
| 15 | 3636329 | 297566 | 400 | Northern | Barbusano forest |
| 16 | 3616589 | 322578 | 300 | Southern | Barbusano forest |
| 17 | 3616421 | 322621 | 300 | Southern | Barbusano forest |
| 18 | 3631060 | 302226 | 450 | Northern | Laurel forest |
| 19 | 3631086 | 302513 | 450 | Northern | Laurel forest |
| 20 | 3631337 | 302598 | 450 | Northern | Laurel forest |
| 21 | 3629558 | 316978 | 620 | Northern | Laurel forest |
| 22 | 3629567 | 316295 | 620 | Northern | Laurel forest |
| 23 | 3621117 | 307848 | 690 | Southern | Barbusano forest |
| 24 | 3621019 | 307834 | 690 | Southern | Barbusano forest |
| 25 | 3621194 | 307862 | 690 | Southern | Barbusano forest |
| 26 | 3628192 | 319096 | 650 | Northern | Laurel forest |
| 27 | 3628682 | 319554 | 600 | Northern | Laurel forest |
| 28 | 3630388 | 318927 | 670 | Northern | Laurel forest |
| 29 | 3630784 | 319118 | 650 | Northern | Laurel forest |
| 30 | 3630644 | 310559 | 500 | Northern | Laurel forest |
| 31 | 3628650 | 321806 | 883 | Northern | Laurel forest |
| 32 | 3623528 | 324470 | 750 | Northern | Laurel forest |
| 33 | 3622223 | 301508 | 700 | Southern | Barbusano forest |
| 34 | 3622230 | 301521 | 700 | Southern | Barbusano forest |
| 35 | 3622242 | 301532 | 700 | Southern | Barbusano forest |
| 36 | 3622440 | 301818 | 800 | Southern | Barbusano forest |
| 37 | 3622351 | 301966 | 700 | Southern | Barbusano forest |
| 38 | 3624407 | 321847 | 850 | Northern | Laurel forest |
| 39 | 3629776 | 318556 | 750 | Northern | Laurel forest |
| 40 | 3630089 | 318779 | 750 | Northern | Laurel forest |
| 41 | 3625713 | 320677 | 1070 | Northern | Laurel forest |
| 42 | 3628458 | 320376 | 900 | Northern | Laurel forest |
| 43 | 3631349 | 305475 | 900 | Northern | Laurel forest |
| 44 | 3632358 | 299102 | 1075 | Northern | Laurel forest |
| 45 | 3624424 | 320624 | 965 | Northern | Laurel forest |
| 46 | 3624114 | 320403 | 950 | Northern | Laurel forest |
| 47 | 3624132 | 319740 | 965 | Northern | Laurel forest |
| 48 | 3623928 | 320259 | 965 | Northern | Laurel forest |
| 49 | 3630936 | 311426 | 950 | Northern | Laurel forest |
| 50 | 3630965 | 312113 | 970 | Northern | Laurel forest |
| 51 | 3622974 | 322642 | 1190 | Northern | Laurel forest |
| 52 | 3626554 | 300810 | 1150 | Northern | Laurel forest |
| 53 | 3628912 | 305690 | 1100 | Northern | Laurel forest |
| 54 | 3628757 | 305904 | 1100 | Northern | Laurel forest |
| 55 | 3626592 | 300581 | 1100 | Northern | Laurel forest |
| 56 | 3626363 | 301032 | 1100 | Northern | Laurel forest |
| 57 | 3626214 | 301517 | 1180 | Northern | Laurel forest |
| 58 | 3628266 | 306493 | 1150 | Northern | Laurel forest |
| 59 | 3628493 | 305923 | 1200 | Northern | Laurel forest |
| 60 | 3628779 | 305587 | 1200 | Northern | Laurel forest |
| 61 | 3629859 | 304128 | 1400 | Northern | Laurel forest |
| 62 | 3629927 | 304431 | 1390 | Northern | Laurel forest |
| 63 | 3629929 | 304370 | 1390 | Northern | Laurel forest |
| 64 | 3629921 | 304038 | 1390 | Northern | Laurel forest |
| 65 | 3629902 | 304064 | 1390 | Northern | Laurel forest |
| 66 | 3629837 | 304072 | 1400 | Northern | Laurel forest |
| 67 | 3629903 | 304244 | 1400 | Northern | Laurel forest |
| 68 | 3621934 | 318880 | 1480 | Southern | Ericaceous forest and thickets |
| 69 | 3621512 | 318619 | 1360 | Southern | Laurel forest |
| 70 | 3626186 | 302057 | 1345 | Northern | Laurel forest |
| 71 | 3626001 | 318052 | 1700 | Northern | High mountain |
| 72 | 3625889 | 318180 | 1750 | Northern | High mountain |
| 73 | 3625719 | 318274 | 1700 | Northern | High mountain |
| 74 | 3626103 | 318148 | 1750 | Northern | High mountain |
| 75 | 3626384 | 318321 | 1700 | Northern | High mountain |
| 76 | 3625326 | 306221 | 1500 | Northern | Ericaceous forest and thickets |
| 77 | 3627529 | 305509 | 1550 | Northern | Ericaceous forest and thickets |
| 78 | 3626756 | 319079 | 1700 | Northern | High mountain |
| 79 | 3626679 | 319382 | 1670 | Northern | High mountain |
| 80 | 3628506 | 303851 | 1500 | Northern | Ericaceous forest and thickets |

Table S2. Species list of bryophytes with functional-taxonomic group information, in grey our owns observations. (NA-not applicable or not observed; acr- acrocarpous; ple-pleurocarpous; tha-thalloid; lea-leafy; a-annual; c-colonist; s-short-lived shuttle; l-long-lived shuttle; p-perennial; cu-cushions; de-dendroids; fa-fans; ma-mats; pe-pendants; tf-turfs; we-wefts; M-monoicous; D-dioicous; MD-monoicous and dioicous; man-mamillose; pap-papillose; smo-smooth; exs-exserted; nex-not exserted; pex-partly exserted; abs-absence; pre-presence).

| **Species** | **Systematic group** | **Growth form** | **Life strategy** | **Generation length** | **Life forms** | **Spores maximum diameter (µm)** | **Sexual condition** | **Length of shoot or thallus (or diameter of rosette (mm))** | **leaf Length max (mm)** | **Laminal/median cells ornamentation** | **Capsule position (in relation to perichaetium)** | **Operculum** | **Peristome** | **References** |
| --- | --- | --- | --- | --- | --- | --- | --- | --- | --- | --- | --- | --- | --- | --- |
| *Acanthocoleus aberrans* | Liverwort | lea | s | 6.7 | ma | 36 | M | 10 | 0.3 | smo | pex | abs | abs | BET; BRYOTRAIT-AZO; Kruijt, 1988 |
| *Anthoceros agrestis* | Hornwort | lea | p | 16.7 | ma | 26 | M | 15 | NA | smo | exs | abs | abs | BET; Hill et al., 2007 |
| *Acrobolbus madeirensis* | Liverwort | tha | a | 3.3 | ma | 62 | D | 15 | 2.5 | smo | exs | abs | abs | BET; Burghardt & Gradstein, 2008; Dierssen, 2002 |
| *Apopellia endiviifolia* | Liverwort | tha | c | 6.7 | ma | 80 | D | 50 | NA | smo | exs | abs | abs | BET; Hill et al., 2007 |
| *Asterella africana* | Liverwort | tha | s | 6.7 | ma | 80 | M | 30 | NA | smo | nex | abs | abs | BET; BRYOTRAIT-AZO |
| *Calypogeia fissa* | Liverwort | lea | c | 6.7 | ma | 14 | M | 30 | 1.8 | smo | exs | abs | abs | BET; BRYOTRAIT-AZO; Hill et al., 2007 |
| *Cephalozia bicuspidata* | Liverwort | lea | c | 6.7 | ma | 15 | M | 30 | 0.8 | smo | exs | abs | abs | BET; BRYOTRAIT-AZO; Hill et al., 2007 |
| *Chiloscyphus pallescens* | Liverwort | lea | p | 16.7 | tf | 22 | M | 60 | 2.3 | smo | exs | abs | abs | BET; BRYOTRAIT-AZO; Hill et al., 2007 |
| *Chiloscyphus polyanthos* | Liverwort | lea | p | 16.7 | ma | 21 | D | 30 | 3.0 | smo | exs | abs | abs | BET; BRYOTRAIT-AZO; Hill et al., 2007 |
| *Cololejeunea microscopica* | Liverwort | lea | s | 6.7 | ma | 30 | M | 6 | 0.345 | smo | pex | abs | abs | BET; BRYOTRAIT-AZO Hill et al., 2007 |
| *Conocephalum conicum* | Liverwort | tha | l | 16.7 | ma | 97 | D | 110 | NA | smo | pex | abs | abs | BET; BRYOTRAIT-AZO; Hill et al., 2007 |
| *Corsinia coriandrina* | Liverwort | tha | s | 6.7 | ma | 150 | D | 20 | NA | smo | pex | abs | abs | BET; Bischler, 2005; BRYOTRAIT-AZO; |
| *Diplophyllum albicans* | Liverwort | lea | c | 6.7 | ma | 15 | D | 60 | 1.8 | smo | exs | abs | abs | BET; BRYOTRAIT-AZO; Hill et al., 2007 |
| *Drepanolejeunea hamatifolia* | Liverwort | lea | l | 16.7 | ma | 36 | MD | 10 | 0.34 | pap | pex | abs | abs | BET; BRYOTRAIT-AZO; Hill et al., 2007 |
| *Exormotheca pustulosa* | Liverwort | tha | s | 6.7 | ma | 65 | MD | 10 | NA | smo | exs | abs | abs | BET; BRYOTRAIT-AZO |
| *Fossombronia angulosa* | Liverwort | tha | a | 3.3 | ma | 52 | D | 25 | 3.5 | smo | exs | abs | abs | BET; BRYOTRAIT-AZO; Hill et al., 2007 |
| *Fossombronia caespitiformis* | Liverwort | tha | a | 3.3 | ma | 65 | M | 10 | 3.0 | smo | exs | abs | abs | BET; BRYOTRAIT-AZO; Hill et al., 2007 |
| Fossombronia echinata | Liverwort | tha | a | 3.3 | ma | 42 | M | 10 | NA | smo | exs | abs | abs | BET; BRYOTRAIT-AZO; Sérgio et al., 2013 |
| *Frullania azorica* | Liverwort | lea | l | 16.7 | ma | 67 | D | 40 | 1.5 | smo | exs | abs | abs | BET; BRYOTRAIT-AZO |
| *Frullania microphylla* | Liverwort | lea | l | 16.7 | ma | 60 | MD | 30 | 0.7 | smo | exs | abs | abs | BET; BRYOTRAIT-AZO; Hill et al., 2007 |
| *Frullania polysticta* | Liverwort | lea | l | 16.7 | ma | 65 | D | 80 | 0.8 | smo | exs | abs | abs | BET; Sim-Sim, 1999 |
| *Frullania tamarisci* | Liverwort | lea | l | 16.7 | ma | 56 | MD | 80 | 1.5 | smo | exs | abs | abs | BET; BRYOTRAIT-AZO; Hill et al., 2007 |
| *Frullania teneriffae* | Liverwort | lea | l | 16.7 | ma | 60 | MD | 60 | 1.6 | smo | exs | abs | abs | BET; BRYOTRAIT-AZO; Hill et al., 2007 |
| *Fuscocephaloziopsis lunulifolia* | Liverwort | lea | c | 6.7 | ma | 12 | D | 30 | 0.32 | smo | exs | abs | abs | BET; BRYOTRAIT-AZO; Hill et al., 2007 |
| *Gongylanthus ericetorum* | Liverwort | lea | l | 16.7 | ma | 12 | D | 10 | 1.0 | pap | exs | abs | abs | BET; BRYOTRAIT-AZO; Hill et al., 2007 |
| *Harpalejeunea molleri* | Liverwort | lea | l | 16.7 | ma | 36 | D | 12 | 0.4 | smo | pex | abs | abs | BET; BRYOTRAIT-AZO; Hill et al., 2007 |
| *Heteroscyphus denticulatus* | Liverwort | lea | l | 16.7 | ma | NA | D | 30 | 1.2 | smo | NA | abs | abs | BET; BRYOTRAIT-AZO |
| *Jubula hutchinsiae* | Liverwort | lea | p | 16.7 | fa | 28 | MD | 40 | 1.3 | smo | exs | abs | abs | BET; BRYOTRAIT-AZO; Hill et al., 2007 |
| *Lejeunea cavifolia* | Liverwort | lea | l | 16.7 | ma | 30 | M | 20 | 0.7 | smo;pap | pex | abs | abs | BET; BRYOATT; Casas et al., 2009 |
| *Lejeunea eckloniana* | Liverwort | lea | p | 16.7 | ma | 36 | M | 20 | 0.9 | smo;pap | pex | abs | abs | BET; BRYOTRAIT-AZO; Hill et al., 2007 |
| *Lejeunea flava* subsp. *moorei* | Liverwort | lea | l | 16.7 | ma | 36 | M | 15 | 0.7 | smo;pap | pex | abs | abs | BET; BRYOTRAIT-AZO |
| *Lejeunea hibernica* | Liverwort | lea | s | 6.7 | ma | 36 | MD | 15 | 0.3 | smo;pap | pex | abs | abs | BRYOTRAIT-AZO; Hill et al., 2007 |
| *Lejeunea lamacerina* | Liverwort | lea | l | 16.7 | ma | 37 | M | 20 | 0.8 | smo | pex | abs | abs | BET; BRYOTRAIT-AZO; Hill et al., 2007 |
| *Lejeunea patens* | Liverwort | lea | s | 6.7 | ma | 28 | M | 15 | 0.55 | smo | pex | abs | abs | BET; BRYOTRAIT-AZO; Hill et al., 2007 |
| *Liochlaena lanceolata* | Liverwort | lea | c | 6.7 | ma | 15 | M | 30 | 1.5 | smo | exs | abs | abs | BET; Hill et al., 2007; Paton, 1999 |
| *Lophocolea bidentata* | Liverwort | lea | p | 16.7 | we | 20 | M | 60 | 1.1 | smo | exs | abs | abs | BET; Casas et al., 2009; Hill et al., 2007 |
| *Lophocolea fragrans* | Liverwort | lea | p | 16.7 | ma | 16 | M | 15 | 0.8 | smo | exs | abs | abs | BET; BRYOTRAIT-AZO; Hill et al., 2007 |
| *Lophocolea heterophylla* | Liverwort | lea | c | 6.7 | ma | 16 | M | 20 | 1.4 | smo | exs | abs | abs | BET; BRYOTRAIT-AZO; Hill et al., 2007 |
| *Lunularia cruciata* | Liverwort | tha | p | 16.7 | ma | 21 | D | 40 | NA | smo | exs | abs | abs | BET; BRYOTRAIT-AZO; Hill et al., 2007 |
| *Mannia androgyna* | Liverwort | tha | l | 16.7 | ma | 80 | MD | 30 | NA | smo | nex | abs | abs | BET; BRYOTRAIT-AZO; Fontinha et al., 2011 |
| *Marchesinia mackaii* | Liverwort | lea | p | 16.7 | ma | 40 | M | 50 | 1.8 | smo | pex | abs | abs | BET; BRYOTRAIT-AZO; Hill et al., 2007 |
| *Marsupella emarginata* | Liverwort | lea | c | 6.7 | tf | 20 | D | 50 | 1.5 | smo | exs | abs | abs | BET; BRYOTRAIT-AZO; Hill et al., 2007 |
| *Metzgeria conjugata* | Liverwort | tha | l | 16.7 | we | 26 | M | 20 | NA | smo | exs | abs | abs | BET; Hill et al., 2007 |
| *Metzgeria furcata* | Liverwort | tha | p | 16.7 | ma | 32 | D | 25 | NA | smo | exs | abs | abs | BRYOTRAIT-AZO; Hill et al., 2007 |
| *Metzgeria leptoneura* | Liverwort | tha | l | 16.7 | we | 24 | D | 100 | NA | smo | exs | abs | abs | BET; BRYOTRAIT-AZO; Hill et al., 2007 |
| *Microlejeunea ulicina* | Liverwort | lea | l | 16.7 | ma | 36 | D | 6 | 0.3 | smo | pex | abs | abs | BET; BRYOTRAIT-AZO; Hill et al., 2007 |
| *Myriocoleopsis minutissima* | Liverwort | lea | l | 16.7 | ma | 17 | M | 4 | 0.25 | smo;man | pex | abs | abs | BET; BRYOTRAIT-AZO; Hill et al., 2007 |
| *Nardia scalaris* | Liverwort | lea | c | 6.7 | ma | 18 | D | 20 | 0.7 | smo | exs | abs | abs | BET; BRYOTRAIT-AZO; BRYOATT |
| *Pellia epiphylla* | Liverwort | tha | c | 6.7 | ma | 116 | M | 50 | NA | smo | exs | abs | abs | BET; BRYOTRAIT-AZO; Hill et al., 2007 |
| *Plagiochasma rupestre* | Liverwort | tha | l | 16.7 | ma | 90 | MD | 20 | NA | smo | nex | abs | abs | BET; Hill et al., 2007 |
| *Plagiochila bifaria* | Liverwort | lea | p | 16.7 | tf | 20 | D | 30 | 1.5 | smo | exs | abs | abs | BET; BRYOTRAIT-AZO; Hill et al., 2007 |
| *Plagiochila exigua* | Liverwort | lea | p | 16.7 | we | 20 | D | 20 | 1.6 | smo | exs | abs | abs | BET; BRYOTRAIT-AZO; Hill et al., 2007 |
| *Plagiochila maderensis* | Liverwort | lea | p | 16.7 | ma | NA | D | 60 | 2.4 | smo | NA | abs | abs | BET; Fontinha et al., 2006 |
| *Plagiochila porelloides* | Liverwort | lea | p | 16.7 | tf | 20 | D | 60 | 3.0 | smo | exs | abs | abs | BET; Casas et al., 2009; Hill et al., 2007; Paton, 1999 |
| *Plagiochila punctata* | Liverwort | lea | p | 16.7 | we | 27 | D | 30 | 1.6 | smo | exs | abs | abs | BET; BRYOTRAIT-AZO; Hill et al., 2007 |
| *Plagiochila retrorsa* | Liverwort | lea | p | 16.7 | ma | 25 | D | 70 | 2.3 | smo | exs | abs | abs | BET; Hill et al., 2007 |
| *Plagiochila spinulosa* | Liverwort | lea | p | 16.7 | we | NA | D | 100 | 2.0 | smo | NA | abs | abs | BET; Hill et al., 2007; Paton, 1999 |
| *Plagiochila stricta* | Liverwort | lea | p | 16.7 | ma | NA | D | 40 | 1.5 | pap | NA | abs | abs | BET; Fontinha et al., 2006; Sim-Sim et al., 2004; |
| *Porella canariensis* | Liverwort | lea | l | 16.7 | ma | 80 | D | 80 | 0.66 | smo | exs | abs | abs | BET; BRYOTRAIT-AZO |
| *Porella inaequalis* | Liverwort | lea | p | 16.7 | ma | NA | D | 80 | 2.5 | smo | NA | abs | abs | BET; Dierssen 2002; Fontinha, 2004 |
| *Porella obtusata* | Liverwort | lea | p | 16.7 | ma | 50 | D | 80 | 2.0 | smo | exs | abs | abs | BET; BRYOTRAIT-AZO; Hill et al., 2007 |
| *Radula aquilegia* | Liverwort | lea | l | 16.7 | ma | 56 | D | 50 | 0.4 | smo | exs | abs | abs | BET; BRYOTRAIT-AZO; Hill et al., 2007 |
| *Radula carringtonii* | Liverwort | lea | l | 16.7 | ma | 40 | D | 40 | 1.5 | smo | exs | abs | abs | BET; BRYOTRAIT-AZO; Hill et al., 2007 |
| *Radula holtii* | Liverwort | lea | c | 6.7 | ma | 35 | M | 20 | 0.9 | smo | exs | abs | abs | BET; BRYOTRAIT-AZO; Hill et al., 2007 |
| *Radula lindenbergiana* | Liverwort | lea | l | 16.7 | ma | 30 | D | 30 | 1.5 | smo | exs | abs | abs | BET; BRYOTRAIT-AZO; Hill et al., 2007 |
| *Radula nudicaulis* | Liverwort | lea | c | 6.7 | ma | 55 | D | 40 | 1.4 | smo | exs | abs | abs | BET; BRYOTRAIT-AZO; Frey et al., 2006 |
| *Radula wichurae* | Liverwort | lea | l | 16.7 | ma | 55 | D | 25 | 1.9 | smo | exs | abs | abs | BET; Fontinha et al., 2011; Hill et al., 2007; |
| *Reboulia hemisphaerica* | Liverwort | tha | l | 16.7 | ma | 86 | M | 40 | NA | smo | nex | abs | abs | BET; BRYOTRAIT-AZO; Hill et al., 2007 |
| *Riccardia chamedryfolia* | Liverwort | tha | c | 6.7 | ma | 22 | M | 40 | NA | smo | exs | abs | abs | BET; BRYOTRAIT-AZO; Hill et al., 2007 |
| *Riccardia multifida* | Liverwort | tha | c | 6.7 | ma | 19 | M | 25 | NA | smo | exs | abs | abs | BET; BRYOTRAIT-AZO; Hill et al., 2007 |
| *Riccia ciliata* | Liverwort | tha | a | 3.3 | ma | 90 | M | 15 | NA | smo | nex | abs | abs | BET; Dierssen ,2002; Casas et al., 2009 |
| *Riccia nigrella* | Liverwort | tha | a | 3.3 | ma | 80 | M | 15 | NA | smo | nex | abs | abs | BET; BRYOTRAIT-AZO; Hill et al., 2007 |
| *Riccia sorocarpa* | Liverwort | tha | a | 3.3 | ma | 95 | M | 20 | NA | smo | nex | abs | abs | BET; BRYOTRAIT-AZO; Hill et al., 2007 |
| *Riccia trabutiana* | Liverwort | tha | a | 3.3 | ma | 85 | M | 5 | NA | smo | nex | abs | abs | BET; BRYOTRAIT-AZO; |
| *Saccogyna viticulosa* | Liverwort | lea | p | 16.7 | ma | 16 | D | 50 | 2.0 | smo | exs | abs | abs | BET; BRYOTRAIT-AZO; Hill et al., 2007 |
| *Scapania compacta* | Liverwort | lea | l | 16.7 | ma | 21 | MD | 25 | 1.5 | pap | exs | abs | abs | BET; BRYOTRAIT-AZO; Hill et al., 2007 |
| *Scapania curta* | Liverwort | lea | c | 6.7 | ma | 15 | D | 15 | 0.84 | som | exs | abs | abs | BET; BRYOTRAIT-AZO; Hill et al., 2007; Paton, 1999 |
| *Scapania gracilis* | Liverwort | lea | p | 16.7 | we | 16 | D | 50 | 2.0 | som | exs | abs | abs | BET; BRYOTRAIT-AZO; Hill et al., 2007 |
| *Scapania nemorea* | Liverwort | lea | p | 16.7 | we | 14 | D | 60 | 2.0 | som | exs | abs | abs | BET; BRYOTRAIT-AZO; Hill et al., 2007; Paton, 1999 |
| *Scapania undulata* | Liverwort | lea | p | 16.7 | ma | 21 | D | 100 | 3.0 | som | exs | abs | abs | BET; BRYOTRAIT-AZO; Hill et al., 2007; Paton, 1999 |
| *Targionia hypophylla* | Liverwort | tha | l | 16.7 | ma | 76 | MD | 25 | NA | som | nex | abs | abs | BET; BRYOTRAIT-AZO; Hill et al., 2007 |
| *Telaranea europaea* | Liverwort | lea | c | 6.7 | ma | 18 | M | 15 | 0.5 | som | exs | abs | abs | BET; BRYOTRAIT-AZO; Hill et al., 2007 |
| *Alleniella complanata* | Moss | ple | p | 16.7 | pe | 26 | D | 50 | 2.0 | smo | exs;nex | pre | pre | BET; BRYOTRAIT-AZO; Hedenäs 1992; Hill et al., 2007 |
| *Amphidium curvipes* | Moss | acr | c | 6.7 | cu | 12.5 | D | 20 | 3.0 | pap | exs | pre | abs | BET; Casas et al.,2006; Dierssen, 2002; Fontinha et al., 2006; Sim-Sim et al., 2017 |
| *Amphidium mougeotii* | Moss | acr | c | 6.7 | cu | 12 | D | 80 | 2.0 | pap | exs | pre | abs | BET; BRYOTRAIT-AZO; Hill et al., 2007 |
| *Andoa berthelotiana* | Moss | ple | p | 16.7 | ma | 18 | D | 100 | 1.8 | pap | exs | pre | pre | BET; BRYOTRAIT-AZO; Hedenäs, 1992 |
| *Anoectangium aestivum* | Moss | acr | c | 6.7 | cu | 16 | D | 73 | 1.8 | pap | exs | pre | abs | BET; BRYOTRAIT-AZO; Hill et al., 2007 |
| *Anoectangium angustifolium* | Moss | acr | c | 6.7 | cu | 13 | D | 10 | 1.2 | pap | exs | pre | abs | Casas et al., 2009; Frey, 2006; |
| *Antitrichia californica* | Moss | ple | l | 16.7 | ma | 22 | D | 50 | 1.5 | smo | exs | pre | pre | BET; Casas et al., 2009; Dierssen, 2002; |
| *Atrichum angustatum* | Moss | acr | s | 6.7 | tf | 14 | D | 30 | 8.0 | pap;smo | exs | pre | pre | BET; BRYOTRAIT-AZO; Hill et al., 2007 |
| *Atrichum undulatum* | Moss | acr | s | 6.7 | tf | 19 | MD | 70 | 9.0 | smo | exs | pre | pre | BET; BRYOTRAIT-AZO; Hill et al., 2007 |
| *Brachytheciastrum velutinum* | Moss | ple | p | 16.7 | ma | 16 | M | 50 | 1.5 | smo | exs | pre | pre | BET; BRYOTRAIT-AZO; Hill et al., 2007 |
| *Brachythecium rutabulum* | Moss | ple | c | 6.7 | ma | 24 | M | 120 | 3.0 | smo | exs | pre | pre | BET; BRYOTRAIT-AZO; Hill et al., 2007 |
| *Bryum canariense* | Moss | acr | c | 6.7 | tf | 16 | M | 50 | 6.0 | smo | exs | pre | pre | BET; BRYOTRAIT-AZO; Hill et al., 2007 |
| *Campylopus fragilis* | Moss | acr | c | 6.7 | cu | 20 | D | 45 | 5.0 | smo | exs | pre | pre | BET; BRYOTRAIT-AZO; Hill et al., 2007 |
| *Campylopus introflexus* | Moss | acr | c | 6.7 | cu | 14 | D | 50 | 6.0 | smo | exs | pre | pre | BET; BRYOTRAIT-AZO; Hill et al., 2007 |
| *Campylopus pilifer* | Moss | acr | l | 16.7 | cu | 17 | D | 50 | 7.0 | smo | exs | pre | pre | BET; BRYOTRAIT-AZO; Hill et al., 2007 |
| *Campylopus pyriformis* | Moss | acr | c | 6.7 | tf | 17 | D | 25 | 3.0 | smo | exs | pre | pre | BET; BRYOTRAIT-AZO; Hill et al., 2007 |
| *Cirriphyllum crassinervium* | Moss | ple | p | 16.7 | ma | 22 | D | 50 | 1.5 | smo | exs | pre | pre | BET; Casas et al., 2009; Hill et al., 2007 |
| *Daltonia splachnoides* | Moss | ple | c | 6.7 | ma | 16 | M | 10 | 2.0 | smo | exs | pre | pre | BET; Hedenäs, 1992; Hill et al., 2007 |
| *Dicranum fuscescens* | Moss | acr | p | 16.7 | tf | 24 | D | 75 | 6.0 | smo | exs | pre | pre | BET; Casas et al., 2006; Hill et al., 2007; Smith, 2004 |
| *Dicranum montanum* | Moss | acr | p | 16.7 | cu | 18 | D | 30 | 2.75 | smo | exs | pre | pre | BET; Casas et al., 2006; Hill et al., 2007; Smith, 2004 |
| *Didymodon insulanus* | Moss | acr | c | 6.7 | tf | 13 | D | 50 | 5.0 | pap | exs | pre | pre | BET; BRYOTRAIT-AZO; Hill et al., 2007 |
| *Diphyscium foliosum* | Moss | acr | f | 3.3 | tf | 8 | D | 5 | 4.0 | man;pap | nex | pre | pre | BET; Hill et al., 2007 |
| *Echinodium spinosum* | Moss | ple | p | 16.7 | we | 14.5 | D | 100 | 3.2 | smo | exs | pre | pre | BET; Hedenäs, 1992; Fontinha et al., 2006 |
| *Epipterygium atlanticum* | Moss | acr | c | 6.7 | tf | 12.5 | D | 4 | 2.1 | smo | exs | pre | pre | BET; Hanusch et al., 2020 |
| *Epipterygium tozeri* | Moss | acr | c | 6.7 | tf | 20 | D | 5 | 3.0 | smo | exs | pre | pre | BET; BRYOTRAIT-AZO; Hill et al., 2007 |
| *Exsertotheca intermedia* | Moss | ple | p | 16.7 | pe | 24 | D | 150 | 2.5 | smo | exs;nex | pre | pre | BET; BRYOTRAIT-AZO; Hedenäs, 1992 |
| *Fissidens asplenioides* | Moss | acr | c | 6.7 | ma | 12 | D | 35 | 4.0 | smo | exs | pre | pre | BET; BRYOTRAIT-AZO; Fontinha et al., 2011 |
| *Fissidens bryoides* | Moss | acr | c | 6.7 | tf | 14 | M | 20 | 2.7 | smo | exs | pre | pre | BET; BRYOTRAIT-AZO; Hill et al., 2007 |
| *Fissidens crassipes* | Moss | acr | l | 16.7 | tf | 28 | MD | 20 | 3.25 | smo | exs | pre | pre | BET; BRYOTRAIT-AZO; Hill et al., 2007; Smith, 2004 |
| *Fissidens crispus* | Moss | acr | c | 3.3 | tf | 18 | MD | 7 | 0.9 | smo | exs | pre | pre | BET; BRYOTRAIT-AZO; Casas et al. 2006; Hill et al., 2007 |
| *Fissidens curvatus* | Moss | acr | c | 6.7 | tf | 19 | D | 8 | 1.9 | smo | exs | pre | pre | BET; BRYOTRAIT-AZO; Fontinha et al., 2011 |
| *Fissidens polyphyllus* | Moss | acr | p | 16.7 | tf | 15 | MD | 150 | 3.25 | smo | exs | pre | pre | BET; Casas et al., 2006; Hill et al., 2007 |
| *Fissidens serrulatus* | Moss | acr | c | 6.7 | tf | 16 | D | 75 | 8.0 | smo | exs | pre | pre | BET; BRYOTRAIT-AZO; Hill et al., 2007 |
| *Fissidens taxifolius* | Moss | acr | c | 6.7 | tf | 18 | MD | 20 | 2.4 | smo | exs | pre | pre | BET; BRYOTRAIT-AZO; Hill et al., 2007; Smith, 2004 |
| *Fissidens viridulus* | Moss | acr | c | 3.3 | tf | 15 | MD | 6 | 0.9 | smo | exs | pre | pre | BET; BRYOTRAIT-AZO; Casas et al., 2006; Hill et al., 2007 |
| *Grimmia pulvinata* | Moss | acr | c | 6.7 | cu | 12 | M | 30 | 1.7 | smo | exs;nex | pre | pre | BET; BRYOTRAIT-AZO; Casas et al., 2006; Hill et al., 2007 |
| *Heterocladium heteropterum* | Moss | ple | p | 16.7 | ma | 16 | D | 30 | 0.7 | pap | exs | pre | pre | BET; BRYOTRAIT-AZO; Hill et al., 2007 |
| *Heterocladium wulfsbergii* | Moss | ple | p | 16.7 | ma | 17 | D | 30 | 0.8 | pap | exs | pre | pre | BET; BRYOTRAIT-AZO; Hill et al., 2007 |
| *Homalia lusitanica* | Moss | ple | p | 16.7 | ma | 14.5 | D | 25 | 2.0 | pap | exs | pre | pre | BET; BRYOTRAIT-AZO |
| *Hookeria lucens* | Moss | ple | p | 16.7 | ma | 16 | M | 60 | 5.0 | som | exs | pre | pre | BET; BRYOTRAIT-AZO; Hill et al., 2007 |
| *Hypnum cupressiforme* | Moss | ple | p | 16.7 | ma | 19 | D | 60 | 2.0 | som | exs | pre | pre | BET; BRYOTRAIT-AZO; Hill et al., 2007 |
| *Hypnum resupinatum* | Moss | ple | p | 16.7 | ma | 24 | D | 50 | 1.2 | som | exs | pre | pre | Casas et al., 2006; Hill et al., 2007 |
| *Hypnum uncinulatum* | Moss | ple | p | 16.7 | ma | 18 | D | 30 | 1.2 | smo | exs | pre | pre | BET; BRYOTRAIT-AZO; Hill et al., 2007 |
| *Imbribryum mildeanum* | Moss | acr | p | 16.7 | tf | 15 | D | 25 | 1.5 | smo | exs | pre | pre | BET; BRYOTRAIT-AZO; Hill et al., 2007 |
| *Isothecium montanum* | Moss | ple | p | 16.7 | we | NA | D | 35 | 2.5 | NA | NA | NA | NA | Draper et al., 2015 |
| *Isothecium prolixum* | Moss | ple | p | 16.7 | we | 21 | D | 90 | 2.3 | smo | exs | pre | pre | BET; BRYOTRAIT-AZO; Hedenäs, 1992 |
| *Kindbergia praelonga* | Moss | ple | p | 16.7 | we | 17 | D | 120 | 1.5 | smo | exs | pre | pre | BET; BRYOTRAIT-AZO; Casas et al., 2006; Hill et al., 2007 |
| *Leptodictyum riparium* | Moss | ple | p | 16.7 | ma | 16 | M | 133 | 6.0 | smo | exs | pre | pre | BET; BRYOTRAIT-AZO; Hill et al., 2007 |
| *Leucobryum albidum* | Moss | acr | p | 16.7 | cu | 16 | D | 25 | 4.0 | smo | exs | pre | pre | BET; BRYOTRAIT-AZO |
| *Leucobryum glaucum* | Moss | acr | p | 16.7 | cu | 18 | D | 200 | 12.0 | smo | exs | pre | pre | BET; BRYOTRAIT-AZO; Hill et al., 2007 |
| *Leucodon sciuroides* | Moss | ple | l | 16.7 | ma | 26 | D | 200 | 3.0 | smo | exs | pre | pre | BET; BRYOTRAIT-AZO; Hill et al., 2007 |
| *Microeurhynchium pumilum* | Moss | ple | p | 16.7 | we | 13 | D | 40 | 0.5 | smo | exs | pre | pre | BET; BRYOTRAIT-AZO; Casas et al. 2006; Hill et al., 2007 |
| *Myurium hochstetteri* | Moss | ple | p | 16.7 | ma | 24 | D | 60 | 4.0 | smo | exs | pre | pre | BET; BRYOTRAIT-AZO; Hill et al., 2007 |
| *Oedipodiella australis* | Moss | acr | s | 6.7 | tf | NA | D | 5 | 1.4 | smo | NA | NA | NA | BET; Casas et al. 2009; Dierssen, 2002 |
| *Oxyrrhynchium hians* | Moss | ple | c | 6.7 | we | 13 | D | 110 | 1.3 | smo | exs | pre | pre | BET; BRYOTRAIT-AZO; Casas et al. 2006; Hill et al., 2007 |
| *Oxyrrhynchium speciosum* | Moss | ple | p | 16.7 | we | 14 | M | 130 | 2.0 | smo | exs | pre | pre | BET; BRYOTRAIT-AZO; Casas et al. 2006; Hill et al., 2007 |
| *Philonotis rigida* | Moss | acr | l | 16.7 | tf | 32 | M | 10 | 0.7 | smo | exs | pre | pre | BET; Casas et al. 2009; Hill et al., 2007 |
| *Plagiomnium affine* | Moss | acr | p | 16.7 | ma | 24 | D | 100 | 3.75 | smo | exs | pre | pre | BET; Casas et al. 2009; Hill et al., 2007 |
| *Plagiomnium rostratum* | Moss | acr | p | 16.7 | ma | 30 | M | 50 | 7.0 | smo | exs | pre | pre | BET; BRYOTRAIT-AZO; Hill et al., 2007 |
| *Plagiomnium undulatum* | Moss | acr | p | 16.7 | tf | 32 | D | 150 | 5.0 | smo | exs | pre | pre | BET; BRYOTRAIT-AZO; Hill et al., 2007 |
| *Plagiomnium undulatum* var. *madeirense* | Moss | acr | p | 16.7 | tf | NA | D | 200 | 10.0 | smo | NA | NA | NA | Kop & Sérgio, 2001; Koponen, 2019 |
| *Plagiothecium nemorale* | Moss | ple | p | 16.7 | ma | 14 | D | 60 | 3.2 | smo | exs | pre | pre | BET; BRYOTRAIT-AZO; Hill et al., 2007 |
| *Plasteurhynchium meridionale* | Moss | ple | p | 16.7 | ma | 18 | D | 30 | 7.0 | smo | exs | pre | pre | BET; BRYOTRAIT-AZO; Hill et al., 2007 |
| *Pogonatum aloides* | Moss | acr | c | 6.7 | tf | 13 | D | 20 | 4.0 | smo | exs | pre | pre | BET; BRYOTRAIT-AZO; Casas et al. 2006; Hill et al., 2007 |
| *Pohlia elongata* | Moss | acr | c | 6.7 | tf | 24 | M | 35 | 1.25 | smo | exs | pre | pre | Casas et al. 2009; Hill et al., 2007 |
| *Polytrichum commune* | Moss | acr | p | 16.7 | tf | 12 | D | 250 | 8.0 | smo | exs | pre | pre | BET; BRYOTRAIT-AZO; Hill et al., 2007 |
| *Polytrichum formosum* | Moss | acr | p | 16.7 | tf | 16 | D | 100 | 13.0 | smo | exs | pre | pre | BET; BRYOTRAIT-AZO; Hill et al., 2007 |
| *Polytrichum piliferum* | Moss | acr | p | 16.7 | tf | 15 | D | 45 | 4.0 | smo | exs | pre | pre | BET; BRYOTRAIT-AZO; Hill et al., 2007 |
| *Pseudoscleropodium purum* | Moss | ple | p | 16.7 | we | 13 | D | 150 | 2.0 | smo | exs | pre | pre | BET; BRYOTRAIT-AZO; Hill et al., 2007 |
| *Pseudotaxiphyllum laetevirens* | Moss | ple | c | 6.7 | ma | NA | MD | 20 | 1.25 | smo | NA | NA | NA | BET; BRYOTRAIT-AZO |
| *Ptychomitrium nigrescens* | Moss | acr | c | 6.7 | tf | 18 | M | 25 | 2.0 | smo | exs | pre | pre | BET; BRYOTRAIT-AZO; Casas et al., 2009; Sim-Sim et al., 2019 |
| *Ptychomitrium polyphyllum* | Moss | acr | c | 6.7 | cu | 14 | M | 40 | 4.0 | smo | exs | pre | pre | BET; BRYOTRAIT-AZO; Hill et al., 2007 |
| *Ptychostomum capillare* | Moss | acr | c | 6.7 | tf | 15 | D | 50 | 3.0 | smo | exs | pre | pre | BET; BRYOTRAIT-AZO; Hill et al., 2007 |
| *Ptychostomum imbricatulum* | Moss | acr | c | 6.7 | tf | 15 | D | 25 | 6.0 | smo | exs | pre | pre | BET; BRYOTRAIT-AZO; Smith, 2004 |
| *Ptychostomum torquescens* | Moss | acr | l | 16.7 | tf | 16 | MD | 25 | 6.0 | smo | exs | pre | pre | BET; BRYOTRAIT-AZO; Hill et al., 2007 |
| *Racomitrium aciculare* | Moss | acr | c | 6.7 | tf | 20 | D | 60 | 3.5 | pap | exs | pre | pre | BET; BRYOTRAIT-AZO; Hill et al., 2007 |
| *Racomitrium affine* | Moss | acr | c | 6.7 | ma | 20 | D | 50 | 1.6 | pap | exs | pre | pre | BET; Casas et al. 2009; Hill et al., 2007 |
| *Racomitrium fasciculare* | Moss | acr | p | 16.7 | ma | 15 | D | 100 | 3.0 | pap | exs | pre | pre | BET; BRYOTRAIT-AZO; BRYOATT; Smith, 2004 |
| *Racomitrium heterostichum* | Moss | acr | c | 6.7 | ma | 18 | D | 50 | 4.0 | pap | exs | pre | pre | BET; BRYOTRAIT-AZO; Hill et al., 2007 |
| *Rhamphidium purpuratum* | Moss | acr | c | 6.7 | tf | 13 | D | 20 | 2.0 | smo | exs | pre | pre | BET; BRYOTRAIT-AZO; Casas et al., 2006; Sérgio et al., 2013 |
| *Rhizomnium punctatum.* | Moss | acr | l | 16.7 | tf | 50 | D | 70 | 6.0 | smo | exs | pre | pre | BET; BRYOTRAIT-AZO; Hill et al., 2007 |
| *Rhynchostegiella pseudolitorea* | Moss | ple | p | 16.7 | ma | 14 | M | 30 | 0.7 | smo | exs | pre | pre | BET; BRYOTRAIT-AZO; Hedenäs, 1992; Hill et al., 2007; Patiño et al., 2017 |
| *Rhynchostegium confertum* | Moss | ple | p | 16.7 | ma | 14 | M | 30 | 1.5 | smo | exs | pre | pre | BET; BRYOTRAIT-AZO; Casas et al., 2006 |
| *Rhynchostegium megapolitanum* | Moss | ple | p | 16.7 | ma | 16 | MD | 40 | 2.5 | smo | exs | pre | pre | BET; BRYOTRAIT-AZO |
| *Rhynchostegium riparioides* | Moss | ple | p | 16.7 | ma | 22 | M | 112 | 1.7 | smo | exs | pre | pre | BET; Casas et al., 2006; Hedenäs 1992; Hill et al., 2007 |
| *Sciuro-hypnum plumosum* | Moss | ple | p | 16.7 | ma | 21 | M | 60 | 2.0 | smo | exs | pre | pre | BET; BRYOTRAIT-AZO; Hill et al., 2007 |
| *Scleropodium touretii* | Moss | ple | p | 16.7 | ma | 15 | D | 80 | 1.5 | smo | exs | pre | pre | BET; BRYOTRAIT-AZO; Hill et al., 2007 |
| *Scorpiurium circinatum* | Moss | ple | p | 16.7 | ma | 18 | D | 60 | 1.0 | smo | exs | pre | pre | BET; BRYOTRAIT-AZO; Hill et al., 2007 |
| *Streblotrichum convolutum* | Moss | acr | c | 6.7 | tf | 10 | D | 10 | 1.7 | pap | exs | pre | pre | BET; BRYOTRAIT-AZO; Hill et al., 2007 |
| *Tetrastichium virens* | Moss | ple | p | 16.7 | ma | 17 | D | 20 | 2.5 | smo | exs | pre | pre | BET; BRYOTRAIT-AZO; Frey, 2006 |
| *Thamnobryum alopecurum* | Moss | ple | p | 16.7 | de | 12 | D | 80 | 2.0 | smo;pap | exs | pre | pre | BET; BRYOTRAIT-AZO; Hill et al., 2007 |
| *Thamnobryum maderense* | Moss | ple | p | 16.7 | de | 15 | D | 120 | 1.25 | smo | exs | pre | pre | BET; Casas et al., 2009; Dierssen, 2002; Hedenäs, 1992 |
| *Thuidium tamariscinum* | Moss | ple | p | 16.7 | we | 20 | D | 140 | 1.25 | smo | exs | pre | pre | BET; BRYOTRAIT-AZO; Hill et al., 2007 |
| *Tortella flavovirens* | Moss | acr | c | 6.7 | cu | 14 | D | 15 | 3.5 | pap | exs | pre | pre | BET; BRYOTRAIT-AZO; Hill et al., 2007 |
| *Tortella nitida* | Moss | acr | p | 16.7 | cu | 15 | D | 10 | 2.5 | pap | exs | pre | pre | BET; BRYOTRAIT-AZO; Hill et al., 2007 |
| *Tortella squarrosa* | Moss | acr | p | 16.7 | ma | 15 | D | 10 | 2.5 | pap | exs | pre | pre | BET; BRYOTRAIT-AZO; Casas et al. 2006; Hill et al., 2007 |
| *Tortella tortuosa* | Moss | acr | p | 16.7 | cu | 16 | D | 40 | 6.5 | pap | exs | pre | pre | BET; BRYOTRAIT-AZO;Hill et al., 2007 |
| *Tortula muralis* | Moss | acr | c | 6.7 | tf | 12 | M | 10 | 2.5 | pap | exs | pre;abs | pre | BET; BRYOTRAIT-AZO; Hill et al., 2007 |
| *Trichostomum brachydontium* | Moss | acr | p | 16.7 | tf | 20 | D | 40 | 2.6 | pap | exs | pre | pre | BET; BRYOTRAIT-AZO; Hill et al., 2007 |
| *Trichostomum crispulum* | Moss | acr | c | 6.7 | tf | 17 | D | 45 | 2.7 | pap | exs | pre | pre | BET; BRYOTRAIT-AZO; Hill et al., 2007 |
| *Ulota crispa* | Moss | acr | s | 6.7 | cu | 26 | M | 20 | 3.5 | pap | exs | pre | pre | BET; BRYOTRAIT-AZO; Hill et al., 2007 |
| *Weissia controversa* | Moss | acr | c | 6.7 | tf | 20 | M | 10 | 2.5 | pap | exs | pre | pre | BET; BRYOTRAIT-AZO; Hill et al., 2007 |

| **References** |
| --- |
| Bischler-Causse, H., Gradstein, S. R., Jovet-Ast, S., Long, D. G., & Allen, N. S. (2005). Marchantiidae. Flora Neotropica Monograph 97. New York, New York Botanical Garden. |
| Burghardt, M., & Gradstein, R. (2008). Chapter Eighteen: A Revision of *Tylimanthus* (Acrobolbaceae, Marchantiophyta) in Tropical America, Africa, and Macaronesia. *Fieldiana Botany*, *2008*(47), 199-210. |
| Casas, C. (2006). Handbook of mosses of the Iberian Peninsula and the Balearic Islands: illustrated keys to genera and species. Institut d'Estudis Catalans. |
| Casas, C. (2009). Handbook of liverworts and hornworts of the Iberian Peninsula and the Balearic Islands: illustrated keys to genera and species. Institut d'Estudis Catalans. |
| Dierssen, K. (2002). Distribution, ecological amplitude and phytosociological-characterization of European bryophytes. *Nova Hedwigia*, *74*(3-4). |
| Draper, I., Hedenäs, L., Stech, M., Patiño, J., Werner, O., González-Mancebo, J. M., ... & Ros, R. M. (2015). How many species of Isothecium (Lembophyllaceae, Bryophyta) are there in Macaronesia? A survey using integrative taxonomy. *Botanical journal of the Linnean society*, *177*(3), 418-438. |
| Fontinha, S. (2004). O género *Porella* L. (Hepaticae) no Arquipélago da Madeira (Doctoral dissertation, Repro 2000, Centro de cópias, Faculdade de ciências da Universidade de Lisboa). |
| Fontinha, S., Sim-Sim, M., & Lobo, C. (2006). The Bryophytes of the Laurisilva of Madeira. Guide to some species. Guia de algumas espécies. Secretaria Regional do Ambiente e dos Recursos Naturais-Serviço do Parque Natural da Madeira. |
| Fontinha, S., Sim-Sim, M., Lobo, C., & Luís, L. (2011). The Bryophytes of Coastal Areas of Madeira. Guide to some species. Secretaria Regional do Ambiente e dos Recursos Naturais-Serviço do Parque Natural da Madeira. |
| Frey, W., Frahm, J. P., Fischer, E., & Lobin, W. (2006). The liverworts, mosses and ferns of Europe. Apollo Books. |
| Hanusch, M., Ortiz, E. M., Patiño, J., & Schaefer, H. (2020). Biogeography and integrative taxonomy of *Epipterygium* (Mniaceae, Bryophyta). *Taxon*, *69*(6), 1150-1171. |
| Hedenäs, L. (1992). Flora of Madeiran pleurocarpous mosses. *Terminology*, *1*, 1. |
| Henriques, D. S., Ah-Peng, C., & Gabriel, R. (2017). Structure and applications of BRYOTRAIT-AZO, a trait database for Azorean bryophytes. *Cryptogamie, Bryologie*, *38*(2), 137-152. |
| Hill, M. O., Preston, C. D., Bosanquet, S. D. S., & Roy, D. B. (2007). BRYOATT: attributes of British and Irish mosses, liverworts and hornworts. Centre for Ecology and Hydrology. |
| Koponen, T. (2019). On the hypothesis of dioicous-monoicous species pairs in the Mniaceae (Bryophyta); morphology, sexual condition and distribution. *Acta Musei Silesiae. Scientiae Naturales*. |
| Koponen, T., & Sérgio, C. (2001). Solving the identity of the large *Plagiomnium* (Musci) from Madeira (Portugal): *P. undulatum* var. *madeirense* T. Kop. & C. Sérgio. *Cryptogamie Bryologie*, *22*(1), 13-18. |
| Kruijt, R. C. (1988). A Monograph of the genera Dicranolejeunea and-Acanthocoleus. *Bryophytorum Bibliotheca*, 36, 1-136, |
| Patiño, J., Hedenäs, L., Dirkse, G. M., Ignatov, M. S., Papp, B., Müller, F., ... Vanderpoorten, A. (2017). Species delimitation in the recalcitrant moss genus *Rhynchostegiella* (Brachytheciaceae). *Taxon*, *66*(2), 293-308. |
| Paton, J. A. (1999). The liverwort flora of the British Isles Harley Books. |
| Sérgio, C., Belsunce, C. A. G., Sim-Sim, M., Vieira, C., Hespanhol, H., & Stow, S. (2013). Atlas e livro vermelho dos briófitos ameaçados de Portugal. Documenta. |
| Sim-Sim, M. (1999). The genus *Frullania* Raddi (Hepaticae) in Portugal and Madeira. *Cryptogamie Bryologie*, *20*(2), 83-144. |
| Sim-Sim, M. E. (2004). Plagiochila stricta Lindenb. new to Madeira Morphological and molecular evidence. *Nova Hedwigia*, 497-505. |
| Sim-Sim, M., Afonina, O. M., Almeida, T., Désamoré, A., Laenen, B., Garcia, C. A., ... Stech, M. (2017). Integrative taxonomy reveals too extensive lumping and a new species in the moss genus *Amphidium* (Bryophyta). *Systematics and biodiversity*, *15*(5), 451-463. |
| Sim-Sim, M., Martins, A., Rodrigues, A. S., Garcia, C. A., Sergio, C., van Rooy, J., ... Gradstein, S. R. (2019). *Ptychomitrium subcrispatum* Thér. & P. de la Varde, an east southern African species excluded from the Cape Verde bryoflora. *Journal of Bryology*, *41*(3), 281-284. |
| Smith, A. J. E. (2004). The moss flora of Britain and Ireland. Cambridge university press.Bischler-Causse, H., Gradstein, S. R., Jovet-Ast, S., Long, D. G., & Allen, N. S. (2005). Marchantiidae. Flora Neotropica Monograph 97. New York, New York Botanical Garden. |
| Van Zuijlen, K., Nobis, M. P., Hedenäs, L., Hodgetts, N., Calleja Alarcón, J. A., Albertos, B., ... & Bergamini, A. (2023). Bryophytes of Europe Traits (BET) data set: A fundamental tool for ecological studies. *Journal of Vegetation Science*, 34(2), e13179. |

Table S3. List of modified species placed in the closest genus and integrated into the genus-level phylogeny.

| **Mosses** | |
| --- | --- |
| *Alleniella complanata*  *Exsertotheca intermedia* | belongs to: Neckera |
| *Imbribryum mildeanum*  *Ptychostomum capillare*  *Ptychostomum imbricatulum*  *Ptychostomum torquescens* | close to: Bryum |
| *Kindbergia praelonga*  *Plasteurhynchium meridionale* | close to: Eurhynchium |
| *Microeurhynchium pumilum* | belongs to: Oxyrrhynchium |
| *Brachytheciastrum velutinum*  *Sciuro-hypnum plumosum* | close to: Brachythecium |
| *Streblotrichum convolutum* | close to: Barbula |
| *Tortella flavovirens*  *Tortella nitida*  *Tortella squarrosa*  *Tortella tortuosa* | close to: Trichostomum |
| **Liverworts** | |
| *Lophocolea bidentata*  *Lophocolea fragrans*  *Lophocolea heterophylla* | close to Heteroscyphus: |

Table S4. List of excluded species from unexamined genera in the phylogeny analyses.

| **Mosses** | **Hornworts/Liverworts** |
| --- | --- |
| *Daltonia splachnoides*  *Hookeria lucens*  *Leucodon sciuroides*  *Oedipodiella australis*  *Pogonatum aloides*  *Rhamphidium purpuratum* | *Anthoceros agrestis*  *Apopellia endiviifolia*  *Fuscocephaloziopsis lunulifolia* |

Table S5. Pairwise correlations between the eight explanatory variables. Correlation values are Pearson coefficients.

|  | **Vegetation type** | **Elevation** | **Geological formation** | **Precipitation** | **Slope** | **Temperature** | **Road distance** |
| --- | --- | --- | --- | --- | --- | --- | --- |
| **Elevation** | 0.82 |  |  |  |  |  |  |
| **Geological formation** | -0.26 | -0.25 |  |  |  |  |  |
| **Precipitation** | 0.46 | 0.55 | -0.33 |  |  |  |  |
| **Slope** | 0.02 | -0.23 | -0.08 | -0.25 |  |  |  |
| **Temperature** | -0.81 | -0.91 | 0.33 | -0.46 | 0.15 |  |  |
| **Road distance** | 0.63 | 0.62 | -0.28 | 0.48 | -0.07 | -0.59 |  |
| **Land use** | 0.13 | 0.36 | -0.04 | 0.23 | -0.2 | -0.34 | 0.35 |

|  | **Vegetation type** | **Elevation** | **Geological formation** | **Precipitation** | **Slope** | **Temperature** | **Road distance** |
| --- | --- | --- | --- | --- | --- | --- | --- |
| **Elevation** | 0.82 |  |  |  |  |  |  |
| **Geological formation** | -0.26 | -0.25 |  |  |  |  |  |
| **Precipitation** | 0.46 | 0.55 | -0.33 |  |  |  |  |
| **Slope** | 0.02 | -0.23 | -0.08 | -0.25 |  |  |  |
| **Temperature** | -0.81 | -0.91 | 0.33 | -0.46 | 0.15 |  |  |
| **Road distance** | 0.63 | 0.62 | -0.28 | 0.48 | -0.07 | -0.59 |  |
| **Land use** | 0.13 | 0.36 | -0.04 | 0.23 | -0.2 | -0.34 | 0.35 |

Table S6. Completeness percentage for the six groups sampled along the elevation gradient on Madeira Island.

| **Completness (%)** | **Jackknife 1** | **Jackknife 2** | **Chao** | **Mean ± SD** |
| --- | --- | --- | --- | --- |
| **Bryophytes** | 80 | 80 | 80 | 80.00 ± 0.00 |
| **Liverworts** | 83.95 | 82.07 | 81.32 | 82.45 ± 1.36 |
| **Mosses** | 81.99 | 76.18 | 80.25 | 79.47 ± 2.98 |
| **Acrocarpous** | 85.84 | 81.24 | 79.28 | 82.12 ± 3.37 |
| **Pleurocarpous** | 74.73 | 64.81 | 66.68 | 68.74 ± 5.27 |
| **Thalloid** | 68.88 | 86.61 | 86.67 | 80.72 ± 10.25 |
| **Leafy** | 80.84 | 86.69 | 85.27 | 84.27 ± 3.05 |

Table S7. Pairwise correlations between TD (Taxonomic diversity), FD (Functional diversity) and PD (Phylogenetic diversity) and to their diversity components (i.e., α and β) for mosses and liverworts. Correlation values are Pearson coefficients. Grey cells indicate correlation values between diversity facets for each component.

|  | **αTDMosses** | **αFDMosses** | **αPDMosses** | **αTDLiverworts** | **αFDLiverworts** | **αPDLiverworts** | **βTDMosses** | **βFDMosses** | **βPDMosses** | **βTDLiverworts** | **βFDLiverworts** |
| --- | --- | --- | --- | --- | --- | --- | --- | --- | --- | --- | --- |
| **αFDMosses** | 0.90 |  |  |  |  |  |  |  |  |  |  |
| **αPDMosses** | 0.89 | 0.81 |  |  |  |  |  |  |  |  |  |
| **αTDLiverworts** | 0.15 | 0.08 | 0.25 |  |  |  |  |  |  |  |  |
| **αFDLiverworts** | 0.17 | 0.08 | 0.25 | 0.94 |  |  |  |  |  |  |  |
| **αPDLiverworts** | 0.22 | 0.11 | 0.31 | 0.90 | 0.91 |  |  |  |  |  |  |
| **βTDMosses** | -0.25 | -0.22 | -0.34 | -0.49 | -0.45 | -0.42 |  |  |  |  |  |
| **βFDMosses** | -0.1 | -0.03 | -0.1 | -0.22 | -0.17 | -0.2 | 0.60 |  |  |  |  |
| **βPDMosses** | -0.44 | -0.39 | -0.45 | -0.19 | -0.23 | -0.25 | 0.59 | 0.48 |  |  |  |
| **βTDLiverworts** | -0.07 | -0.04 | -0.25 | -0.73 | -0.71 | -0.69 | 0.41 | 0.12 | 0.25 |  |  |
| **βTFDLiverworts** | -0.12 | -0.04 | -0.24 | -0.68 | -0.72 | -0.72 | 0.38 | 0.04 | 0.3 | 0.85 |  |
| **βPDLiverworts** | -0.05 | 0.04 | -0.21 | -0.71 | -0.73 | -0.77 | 0.32 | 0.06 | 0.17 | 0.88 | 0.88 |

Table S8. Pairwise correlations between TD (Taxonomic diversity), FD (Functional diversity) and PD (Phylogenetic diversity) and to their diversity components (i.e., α and β) for the four groups (acrocarpous mosses (acr), pleurocarpous mosses (ple), thalloid liverworts (tha), leafy liverworts (lea)). Correlation values are Pearson coefficients. Grey cells indicate correlation values between diversity for each component.

|  | **αTDacr** | **αFDacr** | **αPDacr** | **αTDple** | **αFDple** | **αPDple** | **αTDtha** | **αFDtha** | **αPDtha** | **αTDlea** | **αFDlea** | **αPDlea** | **βTDacr** | **βFDacr** | **βPDacr** | **βTDple** | **βFDple** | **βPDple** | **βTDtha** | **βFDtha** | **βPDtha** | **βTDlea** | **βFDlea** |
| --- | --- | --- | --- | --- | --- | --- | --- | --- | --- | --- | --- | --- | --- | --- | --- | --- | --- | --- | --- | --- | --- | --- | --- |
| **αFDacr** | 0.9 |  |  |  |  |  |  |  |  |  |  |  |  |  |  |  |  |  |  |  |  |  |  |
| **αPDacr** | 0.9 | 0.84 |  |  |  |  |  |  |  |  |  |  |  |  |  |  |  |  |  |  |  |  |  |
| **αTDple** | -0.04 | -0.09 | 0.03 |  |  |  |  |  |  |  |  |  |  |  |  |  |  |  |  |  |  |  |  |
| **αFDple** | -0.02 | -0.1 | 0.04 | 0.94 |  |  |  |  |  |  |  |  |  |  |  |  |  |  |  |  |  |  |  |
| **αPDple** | -0.02 | -0.09 | 0.08 | 0.94 | 0.92 |  |  |  |  |  |  |  |  |  |  |  |  |  |  |  |  |  |  |
| **αTDtha** | 0.44 | 0.44 | 0.23 | -0.17 | -0.22 | -0.26 |  |  |  |  |  |  |  |  |  |  |  |  |  |  |  |  |  |
| **αFDtha** | 0.4 | 0.39 | 0.22 | -0.08 | -0.12 | -0.17 | 0.97 |  |  |  |  |  |  |  |  |  |  |  |  |  |  |  |  |
| **αPDtha** | 0.41 | 0.35 | 0.27 | 0.06 | 0.04 | 0 | 0.87 | 0.92 |  |  |  |  |  |  |  |  |  |  |  |  |  |  |  |
| **αTDlea** | -0.25 | -0.23 | -0.04 | 0.36 | 0.37 | 0.44 | -0.44 | -0.4 | -0.32 |  |  |  |  |  |  |  |  |  |  |  |  |  |  |
| **αFDlea** | -0.26 | -0.24 | -0.04 | 0.42 | 0.44 | 0.48 | -0.44 | -0.38 | -0.28 | 0.97 |  |  |  |  |  |  |  |  |  |  |  |  |  |
| **αPDlea** | -0.34 | -0.34 | -0.1 | 0.39 | 0.4 | 0.45 | -0.52 | -0.47 | -0.37 | 0.96 | 0.96 |  |  |  |  |  |  |  |  |  |  |  |  |
| **βTDacr** | -0.3 | -0.33 | -0.35 | -0.06 | -0.1 | -0.07 | -0.17 | -0.2 | -0.15 | -0.2 | -0.21 | -0.13 |  |  |  |  |  |  |  |  |  |  |  |
| **βFDacr** | -0.33 | -0.38 | -0.37 | -0.06 | -0.04 | -0.01 | -0.14 | -0.18 | -0.13 | -0.03 | 0 | 0.02 | 0.51 |  |  |  |  |  |  |  |  |  |  |
| **βPDacr** | -0.55 | -0.62 | -0.54 | 0.07 | 0.07 | 0.11 | -0.43 | -0.43 | -0.36 | 0.14 | 0.13 | 0.19 | 0.66 | 0.67 |  |  |  |  |  |  |  |  |  |
| **βTDple** | 0 | -0.13 | 0 | 0.45 | 0.55 | 0.45 | -0.26 | -0.16 | -0.07 | 0.03 | 0.12 | 0.09 | 0.01 | -0.01 | 0.07 |  |  |  |  |  |  |  |  |
| **βFDple** | 0.16 | 0.13 | 0.08 | -0.3 | -0.26 | -0.42 | 0.31 | 0.29 | 0.19 | -0.39 | -0.4 | -0.38 | -0.07 | -0.18 | -0.22 | 0.15 |  |  |  |  |  |  |  |
| **βPDple** | 0.21 | 0.25 | 0.15 | -0.29 | -0.33 | -0.38 | 0.36 | 0.32 | 0.25 | -0.39 | -0.37 | -0.44 | 0.02 | -0.06 | -0.16 | -0.1 | 0.55 |  |  |  |  |  |  |
| **βTDtha** | 0.29 | 0.27 | 0.17 | 0.07 | 0.05 | -0.01 | 0.78 | 0.87 | 0.87 | -0.27 | -0.21 | -0.32 | -0.29 | -0.23 | -0.39 | 0 | 0.24 | 0.28 |  |  |  |  |  |
| **βFDtha** | 0.28 | 0.26 | 0.17 | 0.11 | 0.1 | 0.03 | 0.76 | 0.85 | 0.86 | -0.24 | -0.17 | -0.28 | -0.29 | -0.22 | -0.38 | 0.02 | 0.22 | 0.26 | 1.00 |  |  |  |  |
| **βPDtha** | 0.26 | 0.26 | 0.18 | 0.15 | 0.13 | 0.09 | 0.72 | 0.8 | 0.89 | -0.19 | -0.13 | -0.23 | -0.21 | -0.16 | -0.35 | 0.04 | 0.15 | 0.18 | 0.92 | 0.93 |  |  |  |
| **βTDlea** | 0.16 | 0.04 | -0.06 | -0.21 | -0.19 | -0.28 | 0.18 | 0.18 | 0.16 | -0.65 | -0.64 | -0.65 | 0.14 | -0.01 | 0.13 | 0.12 | 0.35 | 0.22 | 0.18 | 0.16 | 0.09 |  |  |
| **βFDlea** | 0.3 | 0.27 | 0.05 | -0.46 | -0.47 | -0.51 | 0.54 | 0.47 | 0.35 | -0.78 | -0.85 | -0.85 | 0.11 | -0.05 | -0.08 | -0.21 | 0.38 | 0.42 | 0.31 | 0.27 | 0.2 | 0.69 |  |
| **βPDlea** | 0.34 | 0.3 | 0.08 | -0.42 | -0.43 | -0.49 | 0.47 | 0.39 | 0.26 | -0.8 | -0.86 | -0.88 | 0.11 | -0.1 | -0.11 | -0.19 | 0.37 | 0.38 | 0.23 | 0.19 | 0.12 | 0.77 | 0.93 |

Table S9. Results of the model selection performed to identify the form of the relationship between the three facets of alpha and beta diversity and the elevation gradient (acrocarpous mosses (acr), pleurocarpous mosses (ple), thalloid liverworts (tha), and leafy liverworts (lea)). Four distinct models were tested namely a null, linear, quadratic and logarithmic linear model (LM). The best models were determined based on the AICc value and are marked in grey. Residual deviance, R2 = the R2 (Null deviance – Residual deviance / Null deviance).

| **LM model** | **null** | **linear** | | | **quadratic** | | | **log** | | |
| --- | --- | --- | --- | --- | --- | --- | --- | --- | --- | --- |
|  | AIC | AIC | R^2^ | p-value | AIC | R^2^ | p-value | AIC | R^2^ | p-value |
| **αTDMosses** | 360.970 | 361.780 | 0.015 | 0.284 | 359.330 | 0.068 | 0.039 | 360.520 | 0.030 | 0.124 |
| **αFDMosses** | 118.750 | 120.730 | <0.001 | 0.891 | 122.280 | 0.005 | 0.512 | 120.740 | <0.001 | 0.971 |
| **αPDMosses** | 1185.900 | 1186.400 | 0.018 | 0.228 | 1178.500 | 0.133 | 0.002 | 1183.600 | 0.052 | 0.041 |
| **αTDLiverworts** | 438.860 | 440.660 | 0.003 | 0.660 | 401.480 | 0.404 | <0.001 | 435.900 | 0.060 | 0.028 |
| **αFDLiverworts** | 181.970 | 183.970 | <0.001 | 0.998 | 137.330 | 0.455 | <0.001 | 179.850 | 0.050 | 0.045 |
| **αPDLiverworts** | 1243.600 | 1244.500 | 0.013 | 0.310 | 1195.100 | 0.481 | <0.001 | 1236.400 | 0.108 | 0.002 |
| **βTDMosses** | -349.970 | -349.040 | 0.013 | 0.308 | -354.650 | 0.103 | 0.007 | -350.780 | 0.035 | 0.099 |
| **βTFDMosses** | -252.520 | -253.230 | 0.021 | 0.198 | -252.420 | 0.036 | 0.286 | -253.350 | 0.023 | 0.183 |
| **βPDMosses** | -261.500 | -259.500 | <0.001 | 0.975 | -263.930 | 0.077 | 0.013 | -260.070 | 0.007 | 0.456 |
| **βTDLiverworts** | -273.800 | -271.830 | <0.001 | 0.861 | -307.430 | 0.375 | <0.001 | -277.330 | 0.066 | 0.021 |
| **βFDLiverworts** | -211.310 | -211.730 | 0.030 | 0.126 | -244.720 | 0.373 | <0.001 | -223.530 | 0.163 | <0.001 |
| **βPDLiverworts** | -151.420 | -152.040 | 0.032 | 0.111 | -204.420 | 0.509 | <0.001 | -167.400 | 0.202 | <0.001 |
| **αTDacr** | 305.74 | 306.760 | 0.012 | 0.329 | 306.540 | 0.039 | 0.145 | 304.430 | 0.011 | 0.073 |
| **αFDacr** | 97.374 | 93.979 | 0.065 | 0.022 | 93.774 | 0.091 | 0.146 | 90.160 | 0.109 | 0.003 |
| **αPDacr** | 1148.300 | 1149.600 | 0.008 | 0.416 | 1151.500 | 0.733 | 0.009 | 1149.400 | 0.010 | 0.367 |
| **αTDple** | 311.520 | 307.380 | 0.074 | 0.015 | 287.380 | 0.297 | <0.001 | 297.110 | 0.185 | <0.001 |
| **αFDple** | 99.045 | 93.310 | 0.092 | 0.006 | 71.958 | 0.322 | <0.001 | 80.797 | 0.224 | <0.001 |
| **αPDple** | 1084.000 | 1076.6 | 0.115 | 0.002 | 1048.300 | 0.394 | <0.001 | 1062.100 | 0.262 | <0.001 |
| **αTDtha** | 236.340 | 217.670 | 0.228 | 0.001 | 210.980 | 0.307 | 0.004 | 201.970 | 0.365 | <0.001 |
| **αFDtha** | 55.206 | 41.511 | 0.178 | <0.001 | 40.388 | 0.209 | 0.084 | 33.667 | 0.255 | <0.001 |
| **αPDtha** | 1151.100 | 1143.300 | 0.115 | 0.002 | 1144.400 | 0.125 | 0.355 | 1139.200 | 0.159 | 0.016 |
| **αTDlea** | 452.890 | 452.570 | 0.028 | 0.134 | 408.320 | 0.455 | <0.001 | 442.360 | 0.145 | <0.001 |
| **αFDlea** | 223.830 | 223.100 | 0.033 | 0.104 | 164.580 | 0.546 | <0.001 | 209.750 | 0.182 | <0.001 |
| **αPDlea** | 1252.000 | 1248.800 | 0.063 | 0.024 | 1195.4 | 0.531 | <0.001 | 1233.200 | 0.228 | <0.001 |
| **βTDacr** | -339.330 | -339.940 | 0.032 | 0.111 | -343.820 | 0.100 | 0.018 | -338.000 | 0.008 | 0.420 |
| **βFDacr** | -168.030 | -166.160 | 0.002 | 0.730 | -164.390 | 0.004 | 0.636 | -166.110 | <0.001 | 0.782 |
| **βPDacr** | -198.520 | -206.600 | 0.118 | 0.002 | -204.610 | 0.118 | 0.909 | -206.880 | 0.121 | 0.001 |
| **βTDple** | -220.110 | -221.940 | 0.045 | 0.054 | -231.430 | 0.174 | <0.001 | -230.270 | 0.141 | <0.001 |
| **βFDple** | -190.680 | -198.080 | 0.110 | 0.002 | -199.230 | 0.145 | 0.083 | -200.880 | 0.141 | <0.001 |
| **βPDple** | -270.010 | -280.520 | 0.144 | <0.001 | -286.330 | 0.224 | 0.006 | -287.450 | 0.216 | <0.001 |
| **βTDtha** | -24.211 | -29.663 | 0.089 | 0.007 | -27.673 | 0.089 | 0.920 | -31.169 | 0.105 | 0.003 |
| **βFDtha** | -40.976 | -45.761 | 0.081 | 0.104 | -43.797 | 0.081 | 0.853 | -46.517 | 0.089 | 0.007 |
| **βPDtha** | -54.079 | -57.493 | 0.065 | 0.022 | -55.623 | 0.067 | 0.724 | -58.095 | 0.072 | 0.016 |
| **βTDlea** | -279.350 | -279.340 | 0.025 | 0.165 | -301.780 | 0.281 | <0.001 | -277.480 | 0.002 | 0.721 |
| **βTFDlea** | -178.390 | -180.440 | 0.049 | 0.048 | -227.570 | 0.485 | <0.001 | -196.880 | 0.225 | <0.001 |
| **βPDlea** | -115.870 | -115.250 | 0.017 | 0.247 | -177.550 | 0.560 | <0.001 | -128.550 | 0.167 | <0.001 |

Table S10. Summary of the best Linear Mixed-Effects Models (LMMs) adjusted using the three facets of α-diversity (TD-Taxonomic diversity, FD-Functional diversity, and PD-Phylogenetic diversity) for the six groups (mosses, liverworts, acrocarpous mosses (acr), pleurocarpous mosses (ple), thalloid liverworts (tha), and leafy liverworts (lea)) as response variables. Only the variables with variance explained <1% were presented. The best models were determined based on the AICc value. The direction of selected variables was assessed from the sign of standardized coefficients. The categorical variables were categorized as follows: Geological formation from the oldest (Upper Volcanic Complex) to the most recent (sedimentary deposits). Vegetation type from coastal and lowland to highe mountain, and Land use from the most artificial (discontinuous urban fabric) to the most natural (mixed forest). Highest variance explained in diversity metric are in grey shaded areas (*p < 0.05).

| **Diversity metric** | **Variable** | **Variance explained ((R2) *100)** | **Coefficient** | **Model varience explained ((R2) *100)** | **AIC** |
| --- | --- | --- | --- | --- | --- |
| **TDMosses** | Geological formation | 3.81 | -0.74 | 8.31 | 362.91 |
|  | Slope | 2.35 | 0.49 |  |  |
|  | Land use | 2.11 | 0.43 |  |  |
| **FDMosses** | Geological formation | 2.62 | -0.63 | 8.59 | 120.44 |
|  | Slope | 3.99 | 0.64 |  |  |
|  | Land use | 2.16 | 0.39 |  |  |
| **PDMosses** | Vegetation type | 1.36 | 0.54 | 12.13 | 1184.33 |
|  | Geological formation | 5.14* | -0.81 |  |  |
|  | Slope | 2.87 | 0.55 |  |  |
| **TDLiverworts** | Vegetation type | 1.49 | -0.77 | 7.93 | 426.73 |
| **FDLiverworts** | Elevation | 2.53 | -0.67 | 8.72 | 169.23 |
|  | Precipitation | 1.34 | 0.04 |  |  |
|  | Road distance | 1.28 | -0.82 |  |  |
| **PDLiverworts** | Road distance | 6.09* | -0.94 | 6.58 | 1230.86 |
| **TDacr** | Geological formation | 4.48* | -0.47 | 15.96 | 300.47 |
|  | Precipitation | 10.84* | -0.71 |  |  |
|  | Land use | 3.41 | 0.29 |  |  |
| **FDacr** | Geological formation | 2.62 | -0.32 | 20.51 | 89.71 |
|  | Precipitation | 14.29* | -0.79 |  |  |
|  | Slope | 1.96 | 0.36 |  |  |
|  | Land use | 3.16 | 0.16 |  |  |
| **PDacr** | Geological formation | 6.01* | -0.63 | 14.51 | 1146.66 |
|  | Precipitation | 5.42* | -0.53 |  |  |
|  | Slope | 2.21 | 0.44 |  |  |
|  | Land use | 1.99 | 0.15 |  |  |
| **TDple** | Precipitation | 3.37 | 0.89 | 12.81 | 308.75 |
|  | Slope | 1.69 | 0.24 |  |  |
| **FDple** | Precipitation | 3.45 | 0.84 | 16.63 | 93.01 |
|  | Slope | 2.02 | 0.31 |  |  |
| **PDple** | Vegetation type | 4.18 | 0.82 | 21.38 | 1073.67 |
|  | Precipitation | 5.75* | 0.86 |  |  |
|  | Slope | 1.78 | 0.23 |  |  |
| **TDtha** | Vegetation type | 24.32* | -0.84 | 49.42 | 193.11 |
|  | Elevation | 1.45 | -0.66 |  |  |
|  | Geological formation | 1.43 | 0.31 |  |  |
|  | Precipitation | 8.31* | -0.82 |  |  |
|  | Road distance | 1.51 | -0.29 |  |  |
| **FDtha** | Vegetation type | 22.91* | -0.88 | 38.88 | 23.75 |
|  | Elevation | 2.82 | -0.66 |  |  |
|  | Precipitation | 8.49* | -0.81 |  |  |
| **PDtha** | Vegetation type | 4.46* | -0.79 | 26.21 | 1135.11 |
|  | Elevation | 1.53 | -0.75 |  |  |
|  | Precipitation | 9.71* | -0.91 |  |  |
| **TDlea** | Elevation | 3.59* | -0.22 | 6.66 | 428.16 |
|  | Precipitation | 5.62 * | 0.39 |  |  |
| **FDlea** | Elevation | 1.36 | -0.24 | 8.46 | 195.63 |
|  | Precipitation | 4.72* | 0.39 |  |  |
| **PDlea** | Precipitation | 4.57* | 0.66 | 8.74 | 1222.22 |
|  | Road distance | 1.59 | -0.48 |  |  |

Table S11. Summary of the Generalized dissimilarity modeling (GDM) used to analyse the drivers of the three facets of β-diversity (TD-Taxonomic diversity, FD-Functional diversity, and PD-Phylogenetic diversity) for the six groups (mosses, liverworts, acrocarpous mosses (acr), pleurocarpous mosses (ple), thalloid liverworts (tha), and leafy liverworts (lea)) in Madeira Island. The direction of selected variables was assessed from the sign of standardized coefficients. Vegetation type was categorized from coastal and lowland to high mountain. Highest contribution in each diversity metric are in grey shaded areas (*p < 0.05).

| **Diversity metric** | **Variable** | **Relative importance** | **Sum of coefficients** | **Model Deviance** | **NULL Deviance** | **Percent Deviance Explained** | **p-value** |
| --- | --- | --- | --- | --- | --- | --- | --- |
| **TDMosses** | Vegetation type | 68.38* | 2.04 | 347.21 | 427.57 | 18.06 | <0.001 |
| **FDMosses** | Elevation | 78.82* | 0.21 | 156.72 | 167.54 | 6.46 | 0.02 |
| **PDMosses** | Elevation | 91.75* | 0.91 | 269.09 | 315.52 | 13.60 | <0.001 |
|  | Slope | 7.39* | 0.32 |  |  |  |  |
| **TDLiverworts** | Vegetation type | 26.01* | 0.68 | 315.92 | 529.03 | 40.28 | <0.001 |
|  | Elevation | 15.89* | 0.85 |  |  |  |  |
| **FDLiverworts** | Vegetation type | 17.28* | 0.67 | 249.1 | 397.12 | 37.27 | <0.001 |
|  | Elevation | 13.19* | 0.94 |  |  |  |  |
| **PDLiverworts** | Vegetation type | 23.97* | 1.17 | 369.14 | 700.49 | 47.31 | <0.001 |
|  | Elevation | 6.93* | 1.12 |  |  |  |  |
| **TDacr** | Elevation | 83.96* | 1.83 | 547.07 | 599.14 | 8.69 | <0.001 |
|  | Precipitation | 5.32* | 0.74 |  |  |  |  |
| **FDacr** | Elevation | 31.94 | 0.26 | 504.09 | 519.39 | 2.79 | 0.420 |
|  | Precipitation | 52.41 | 0.28 |  |  |  |  |
| **PDacr** | Elevation | 72.60* | 0.56 | 585.36 | 610.28 | 4.08 | <0.001 |
|  | Slope | 19.90* | 0.52 |  |  |  |  |
| **TDple** | Vegetation type | 30.04* | 1.37 | 1188.79 | 1295.99 | 8.28 | <0.001 |
|  | Elevation | 31.11* | 1.21 |  |  |  |  |
| **FDple** | Vegetation type | 17.96* | 0.94 | 1108.76 | 1327.97 | 16.51 | <0.001 |
|  | Elevation | 9.60* | 0.94 |  |  |  |  |
| **PDple** | Vegetation type | 8.34* | 0.61 | 1069.57 | 1262.91 | 15.03 | <0.001 |
|  | Elevation | 21.98* | 1.47 |  |  |  |  |
| **TDtha** | Vegetation type | 80.35* | 2.18 | 2978.64 | 3364.82 | 11.43 | <0.001 |
| **FDtha** | Vegetation type | 82.81* | 1.69 | 2936.39 | 3224.75 | 8.94 | <0.001 |
| **PDtha** | Vegetation type | 38.27* | 0.98 | 3229.01 | 3441.69 | 6.17 | <0.001 |
|  | Precipitation | 4.91* | 0.51 |  |  |  |  |
| **TDlea** | Vegetation type | 26.28* | 2.42 | 390.18 | 669.86 | 41.75 | <0.001 |
|  | Elevation | 18.82* | 2.87 |  |  |  |  |
| **FDlea** | Vegetation type | 17.42* | 1.13 | 441.59 | 753.96 | 41.43 | <0.001 |
|  | Elevation | 12.15* | 1.65 |  |  |  |  |
| **PDlea** | Vegetation type | 35.52* | 1.605 | 431.45 | 983.23 | 56.11 | <0.001 |
|  | Elevation | 16.55* | 1.57 |  |  |  |  |


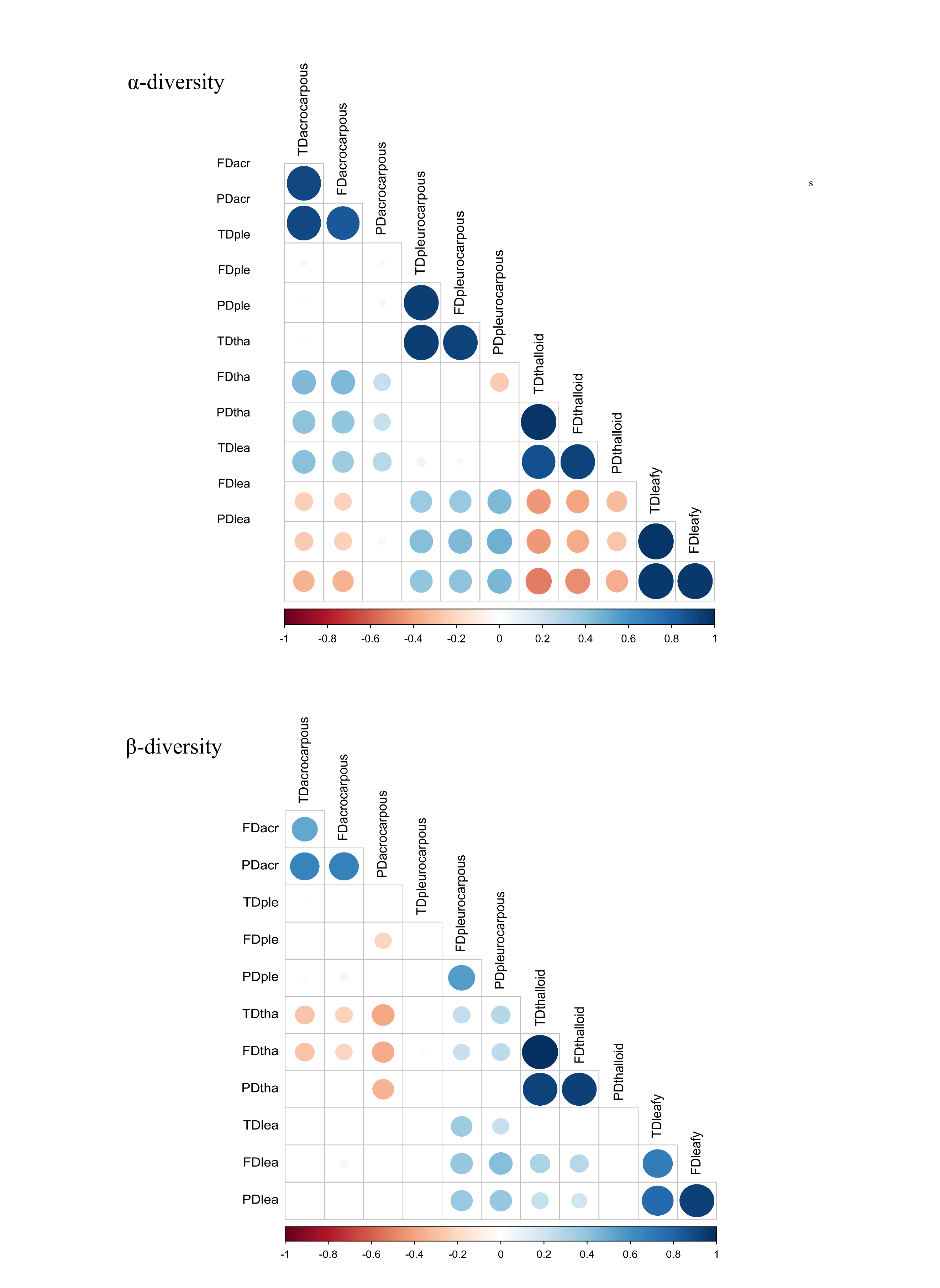


Figure S1. Correlogram of the three facets (TD-Taxonomic diversity, FD-Functional diversity, and PD-Phylogenetic diversity) of α- and β-diversities for acrocarpous mosses (acr), pleurocarpous mosses (ple), thalloid liverworts (tha), and leafy liverworts (lea), on Madeira Island. Only significant correlations (p < 0.05) are colored. The size of the dots represents the strength of the correlations, with larger circles indicating stronger correlations.


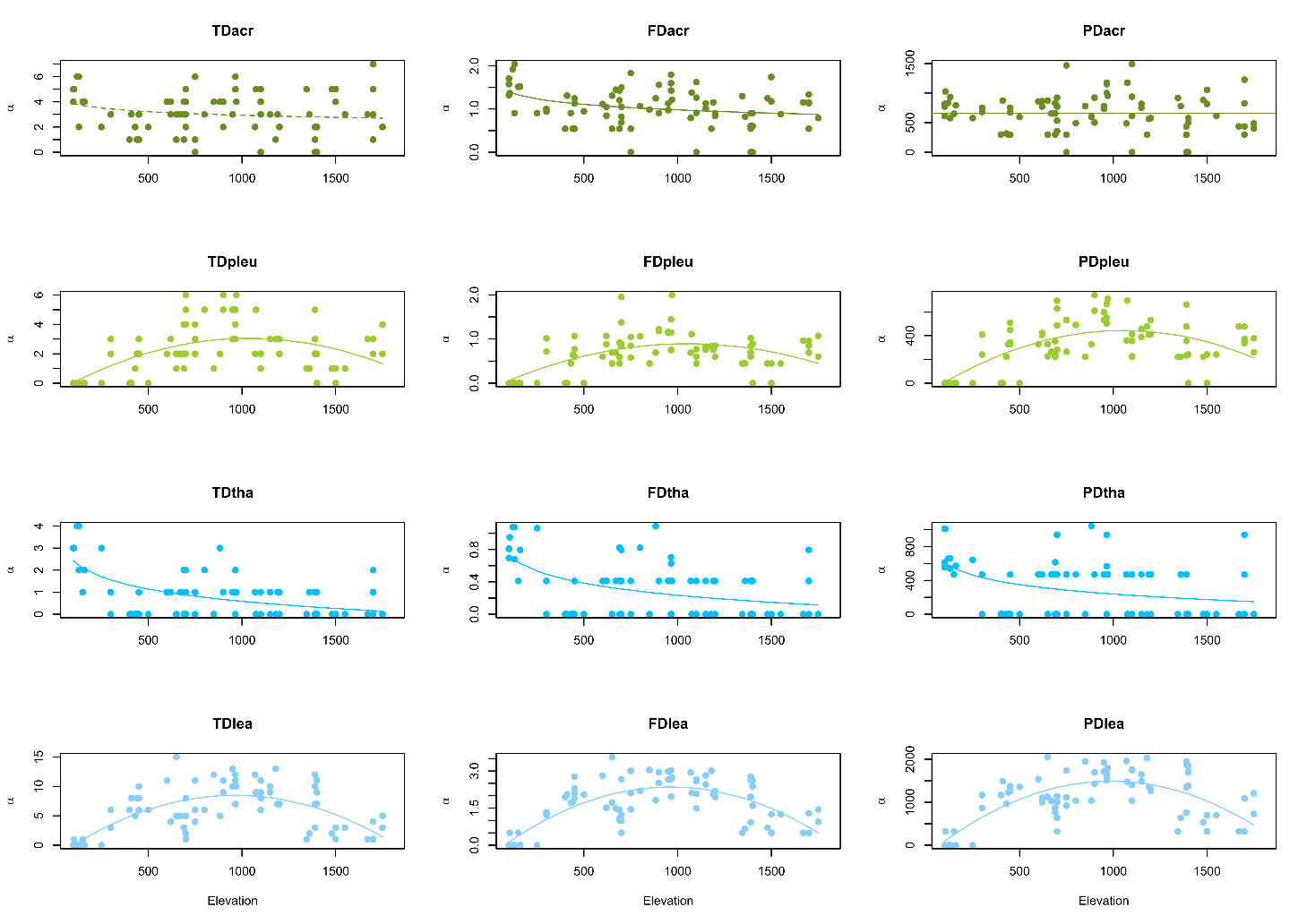


Figure S2. Response plots of the three facets (TD-Taxonomic diversity, FD-Functional diversity, and PD-Phylogenetic diversity) of α-diversity to the elevational gradient in Madeira Island for acrocarpous mosses (acr), pleurocarpous mosses (ple), thalloid liverworts (tha), and leafy liverworts (lea). Solid lines indicate significant relationship between the three facets diversity and elevation while dashed lines indicate non-significant relationships.


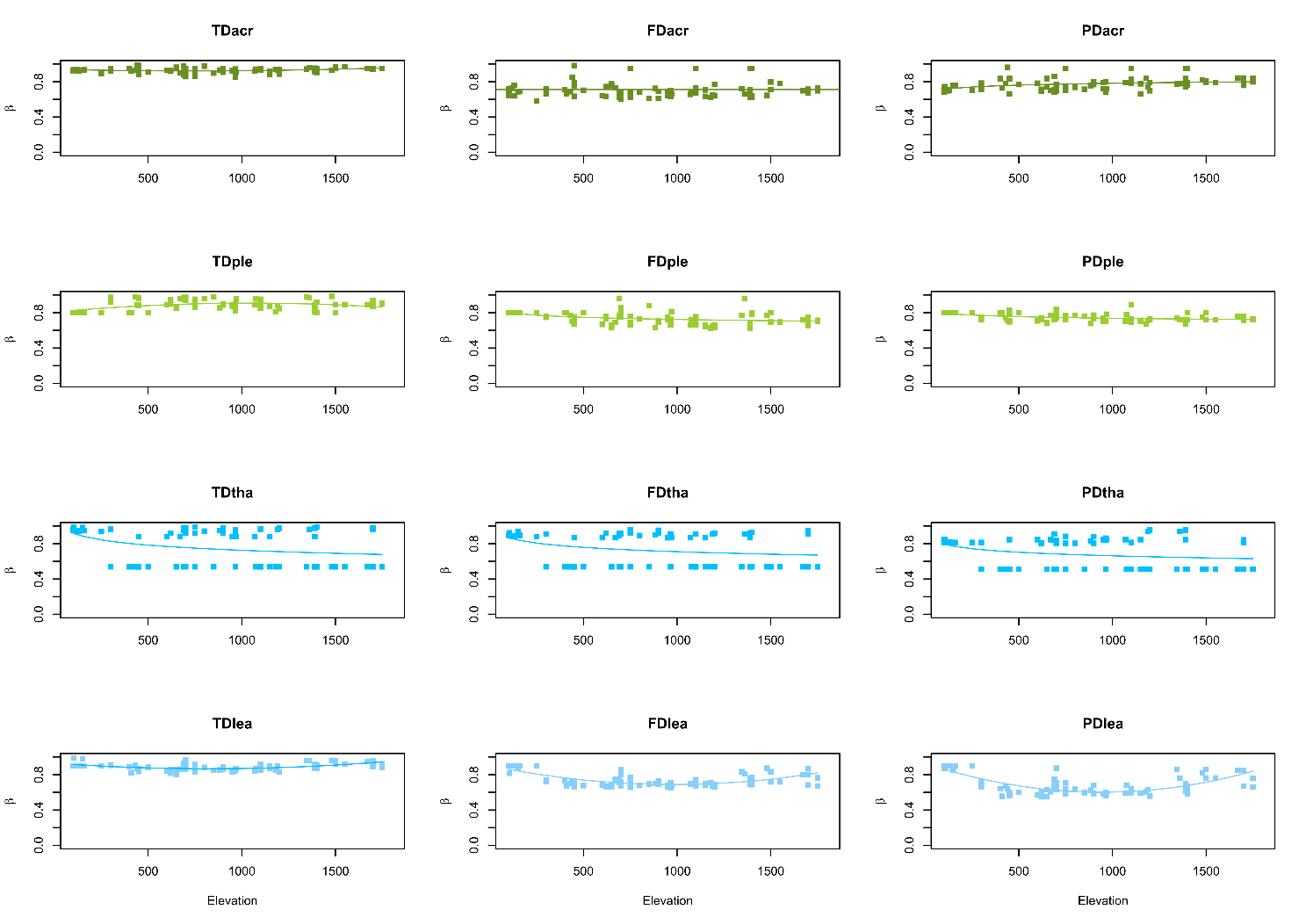
Figure S3. Response plots of the three facets (TD-Taxonomic diversity, FD-Functional diversity, and PD-Phylogenetic diversity) of β-diversity to the elevational gradient in Madeira Island for acrocarpous mosses (acr), pleurocarpous mosses (ple), thalloid liverworts (tha), and leafy liverworts (lea). Solid lines indicate significant relationship between the three facets diversity and elevation while dashed lines indicate non-significant relationships.


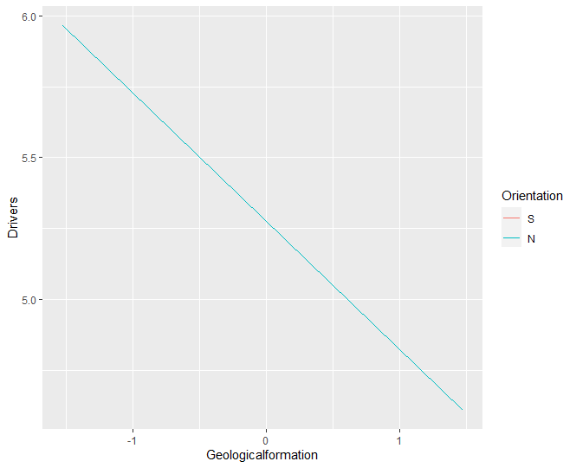


TDMosses


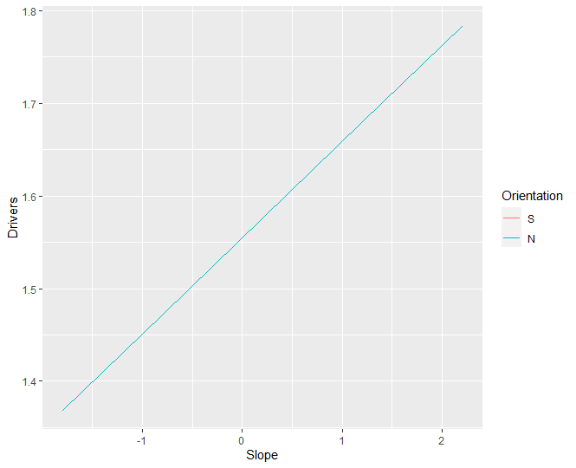


FDMosses


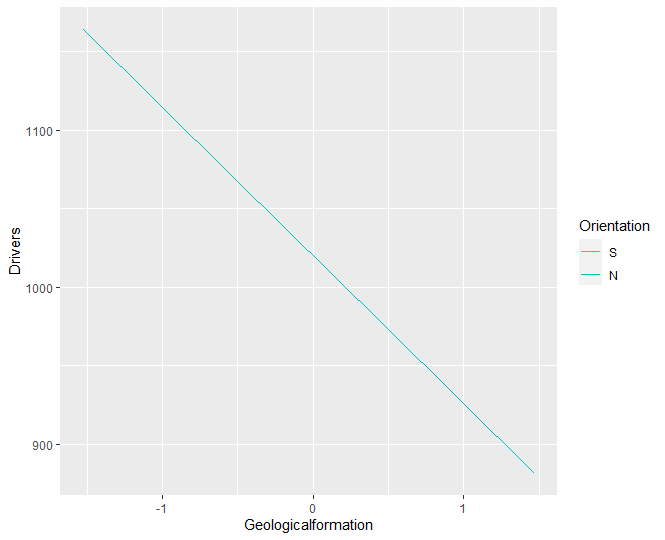


PDMosses


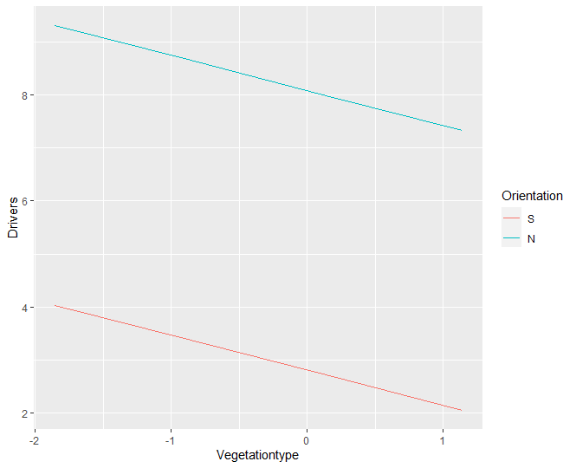


TDLiverworts


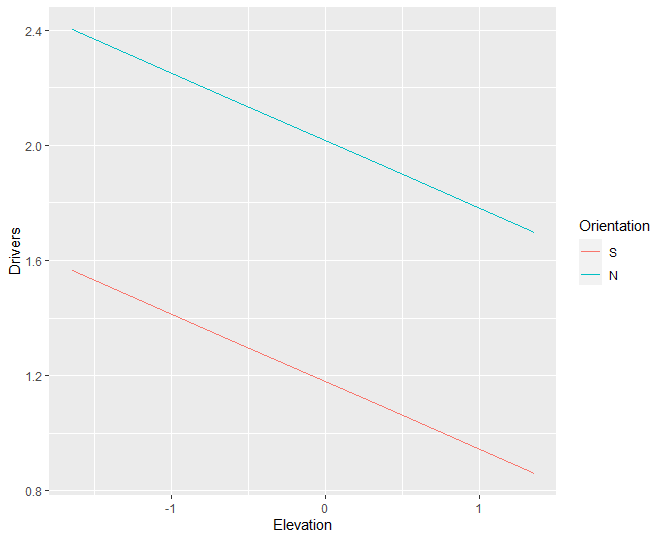


FDLiverworts


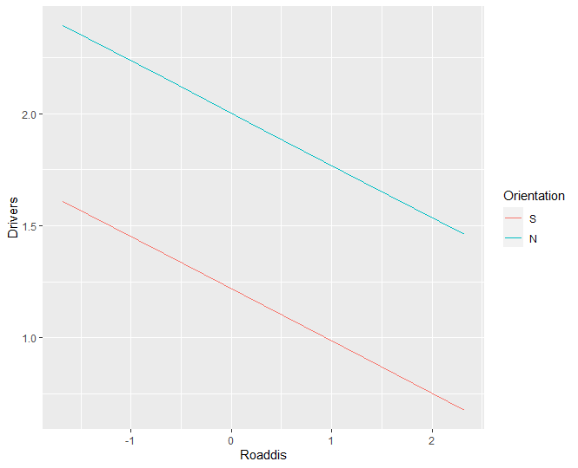


PDLiverworts

Figure S4. Response curves resulting from the Linear Mixed-Effects Models (LMMs) for the three facets (TD-Taxonomic diversity, FD-Functional diversity, and PD-Phylogenetic diversity) of α-diversity in the six groups (mosses, liverworts, acrocarpous mosses (acr), pleurocarpous mosses (ple), thalloid liverworts (tha), and leafy liverworts (lea)) for the predictor with the highestvariance explained in each model. Orientation corresponds to the random effect (N-north vs S-south). (continued on the next page)


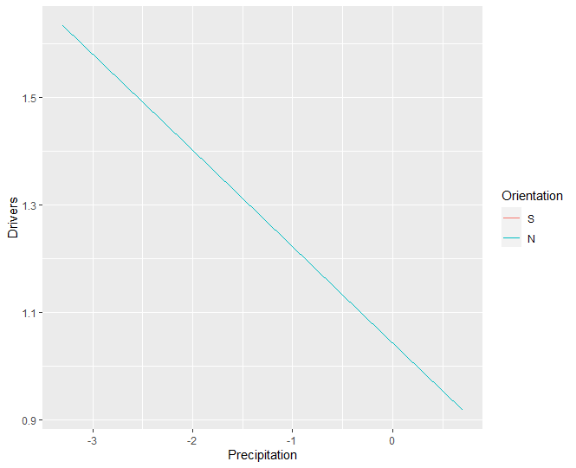


FDacr


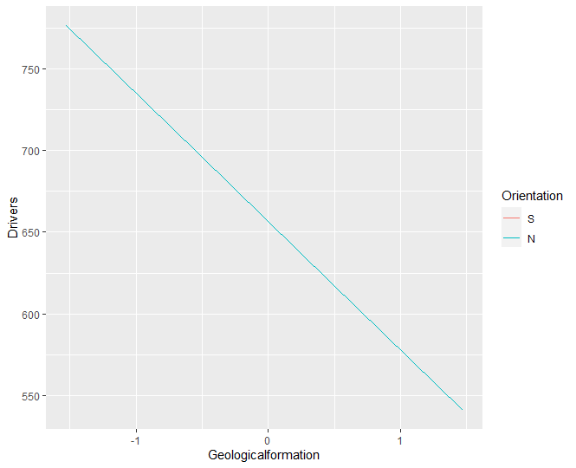


PDacr


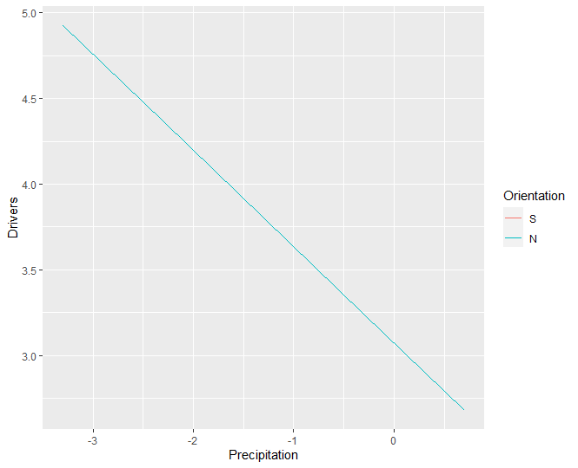


TDacr


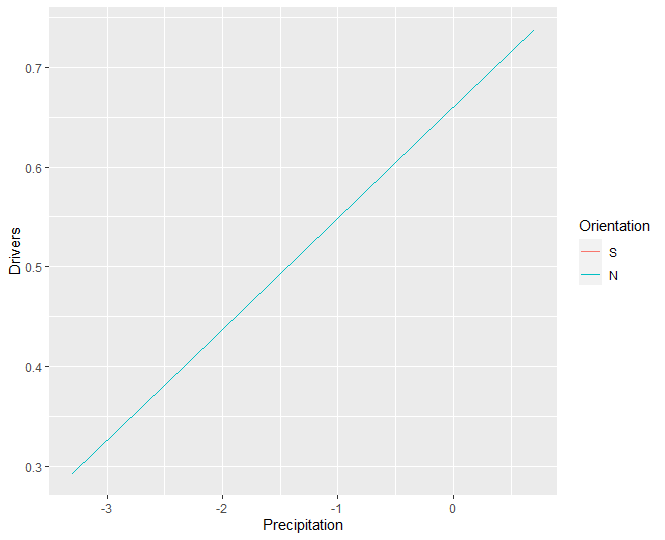

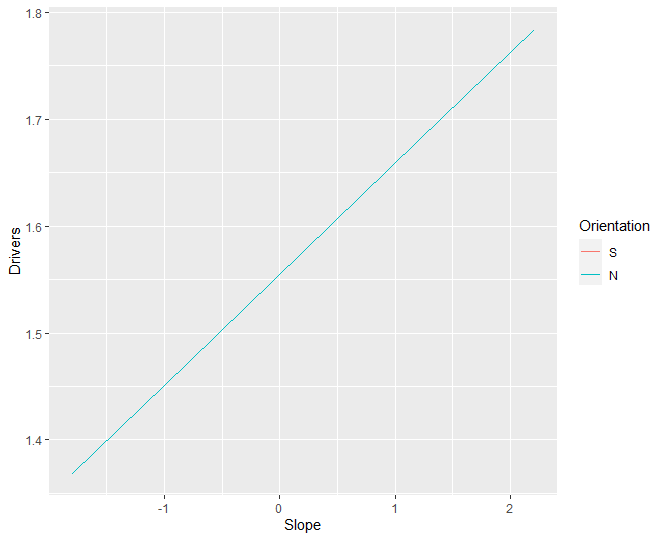

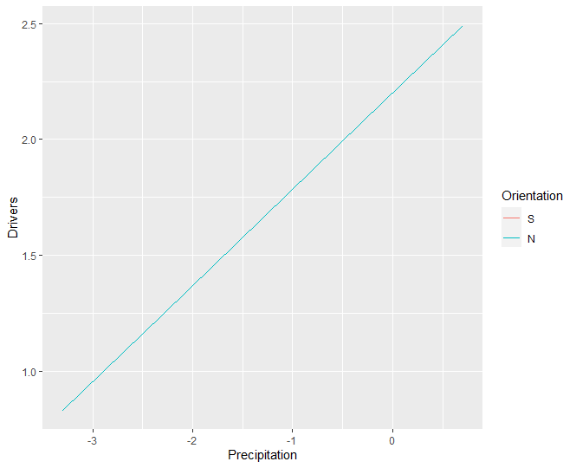


TDple

TDple

TDple

Figure S4. Response curves resulting from the Linear Mixed-Effects Models (LMMs) for the three facets (TD-Taxonomic diversity, FD-Functional diversity, and PD-Phylogenetic diversity) of α-diversity in the six groups (mosses, liverworts, acrocarpous mosses (acr), pleurocarpous mosses (ple), thalloid liverworts (tha), and leafy liverworts (lea)) for the predictor with the highestvariance explained in each model. Orientation corresponds to the random effect (N-north vs S-south). (continued on the next page)


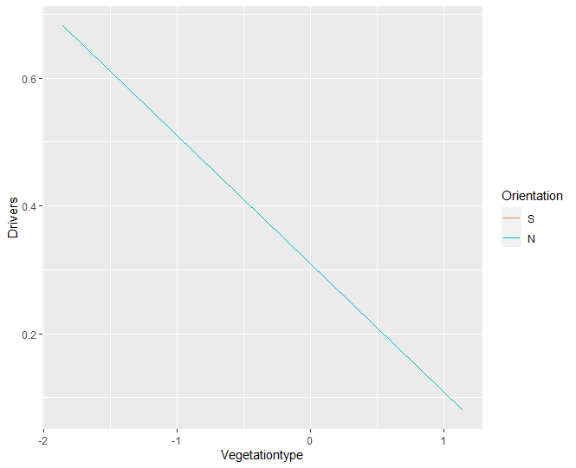

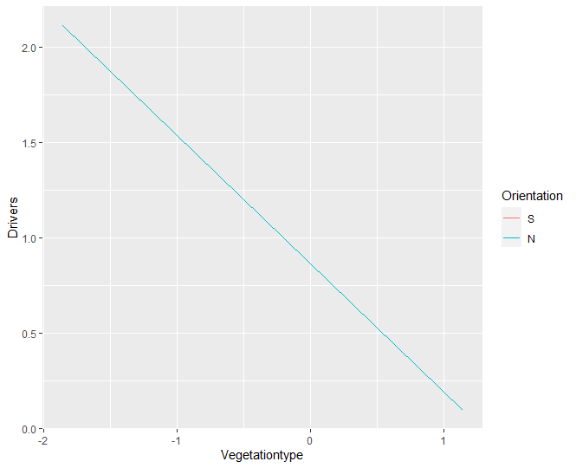


TDtha

FDtha


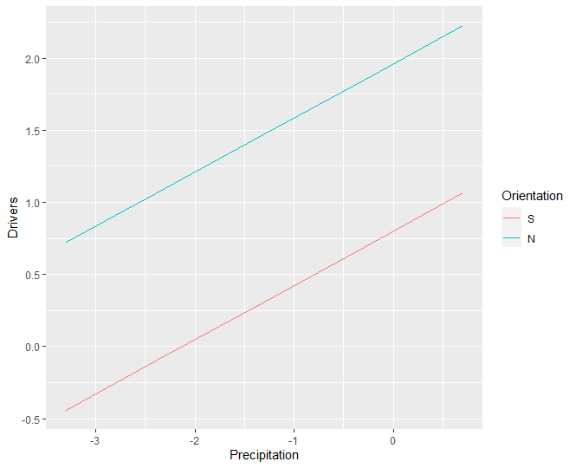

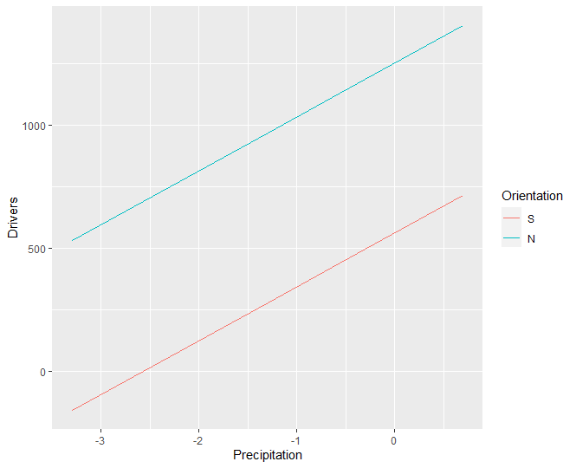

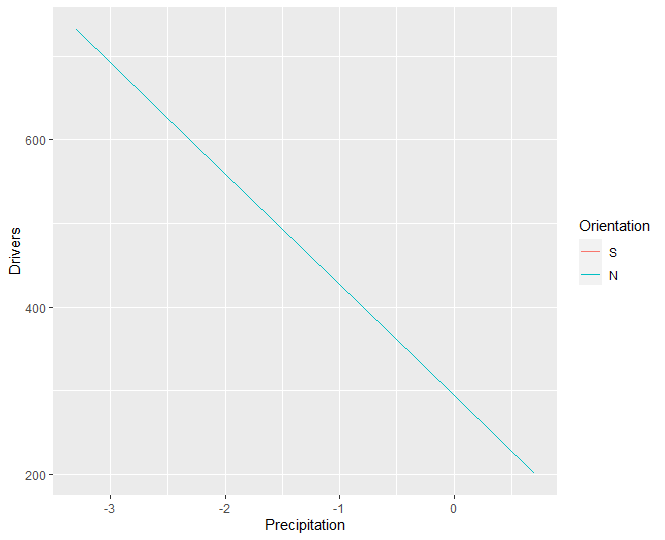


PDtha


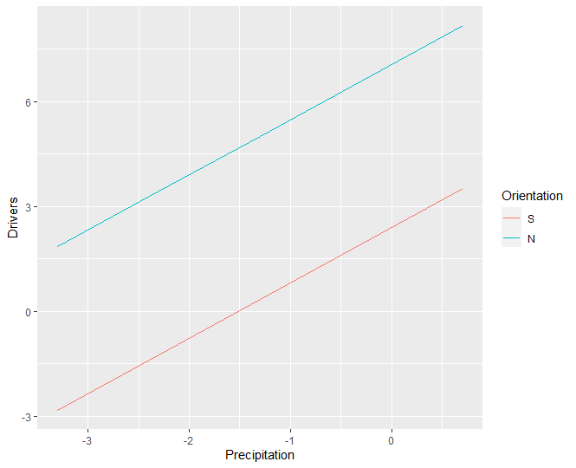


TDleaa

FDleaa

PDleaa

Figure S4. Response curves resulting from the Linear Mixed-Effects Models (LMMs) for the three facets (TD-Taxonomic diversity, FD-Functional diversity, and PD-Phylogenetic diversity) of α-diversity in the six groups (mosses, liverworts, acrocarpous mosses (acr), pleurocarpous mosses (ple), thalloid liverworts (tha), and leafy liverworts (lea)) for the predictor with the highestvariance explained in each model. Orientation corresponds to the random effect (N-north vs S-south).


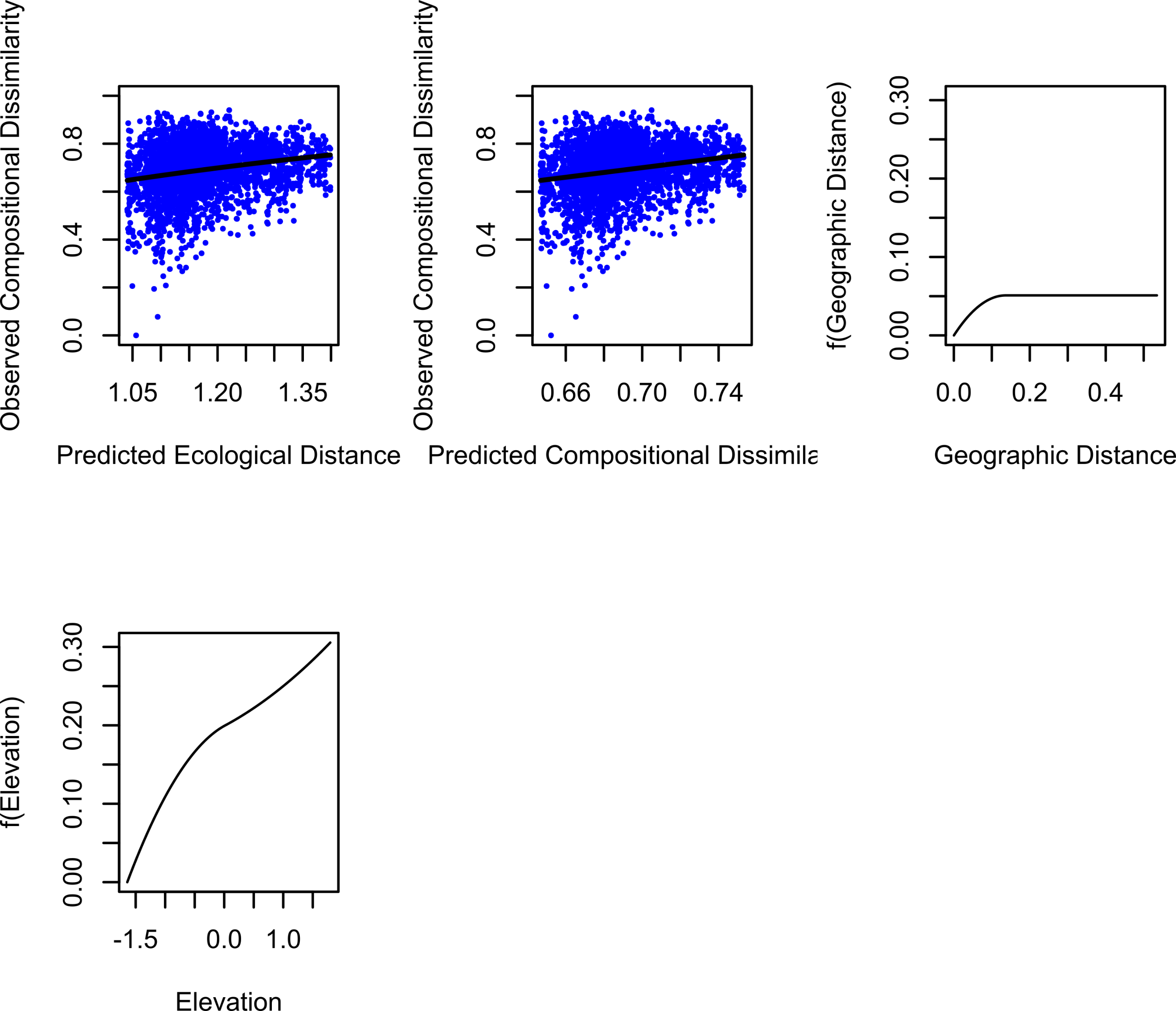

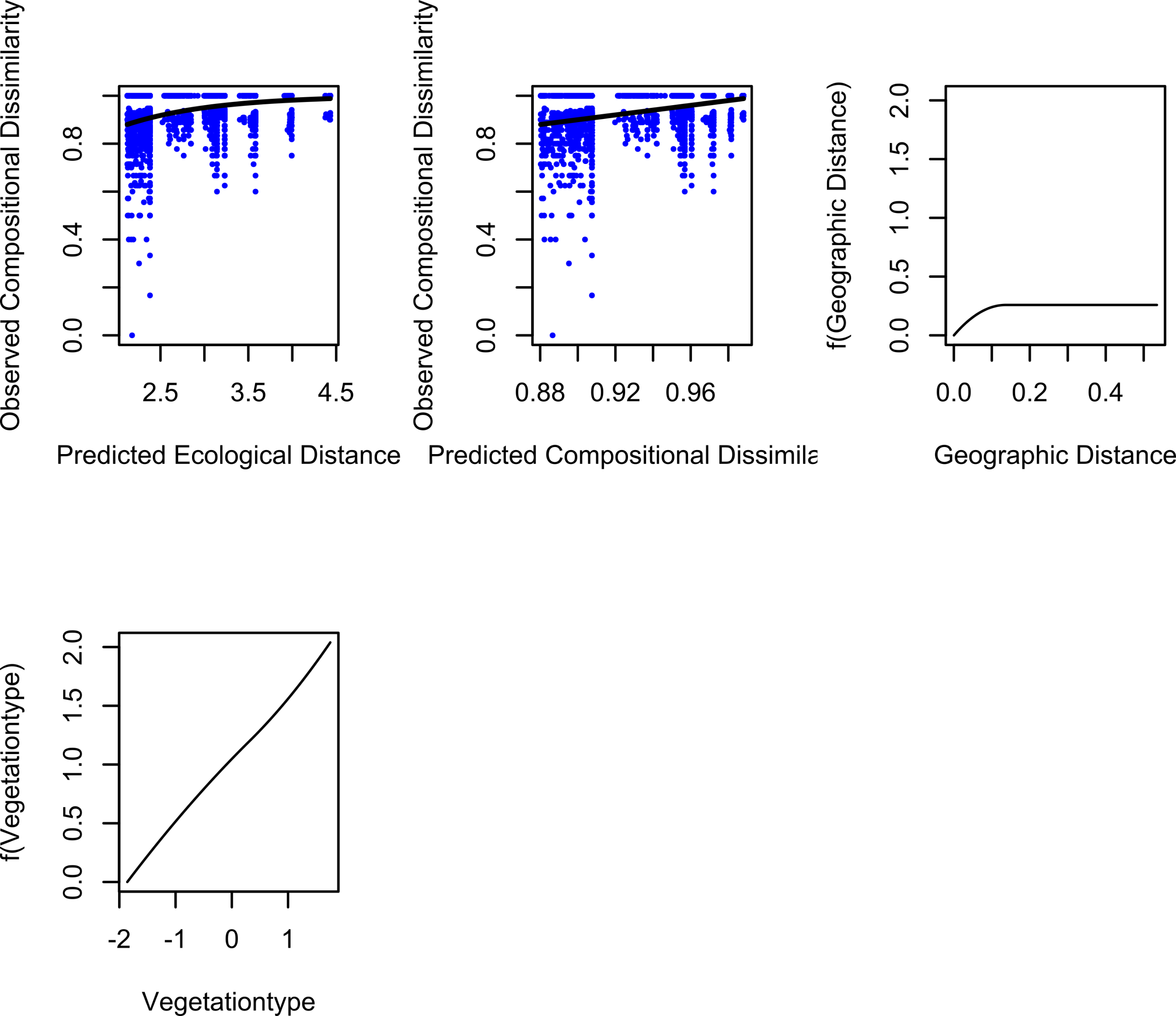


TDMosses

FDMosses


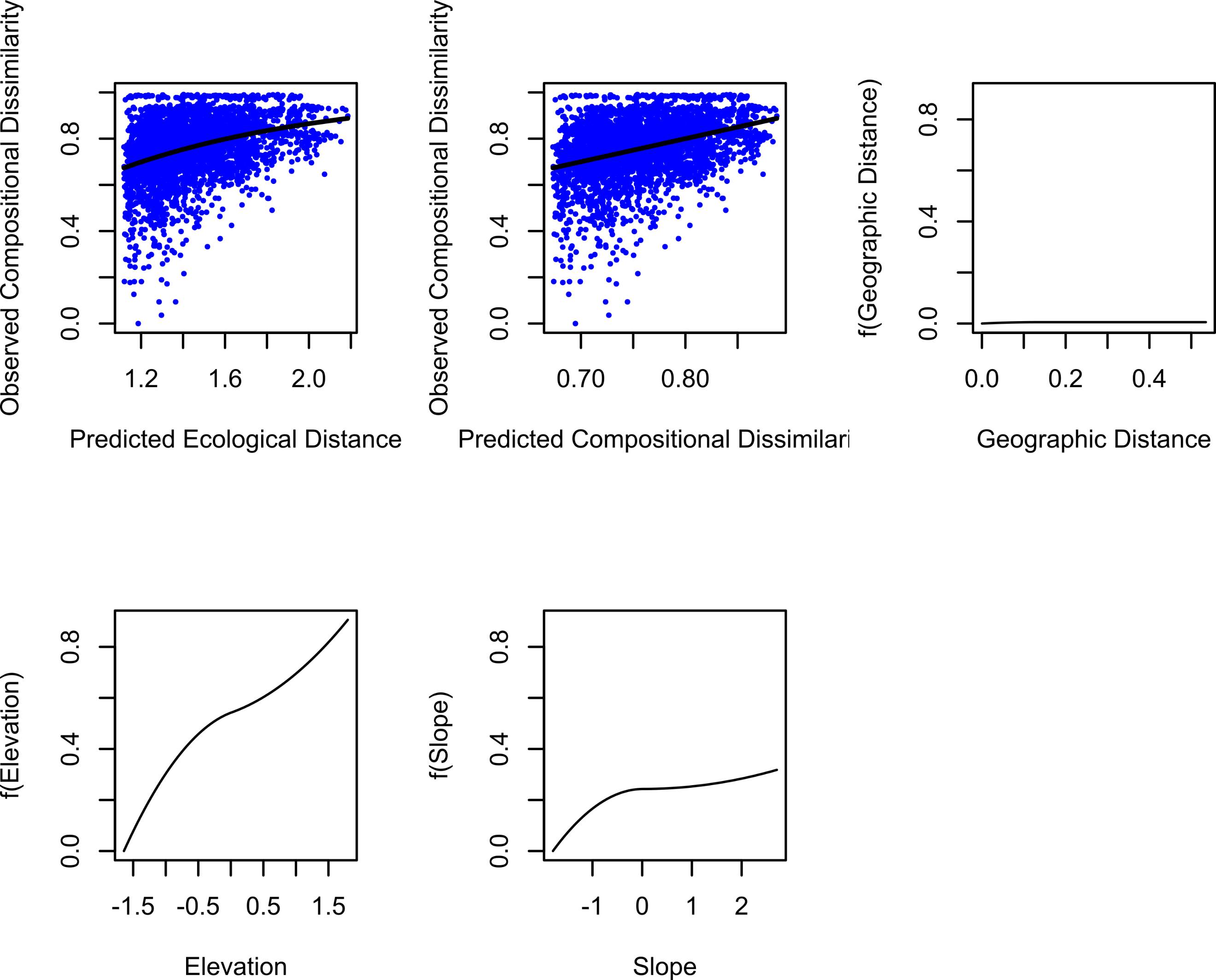

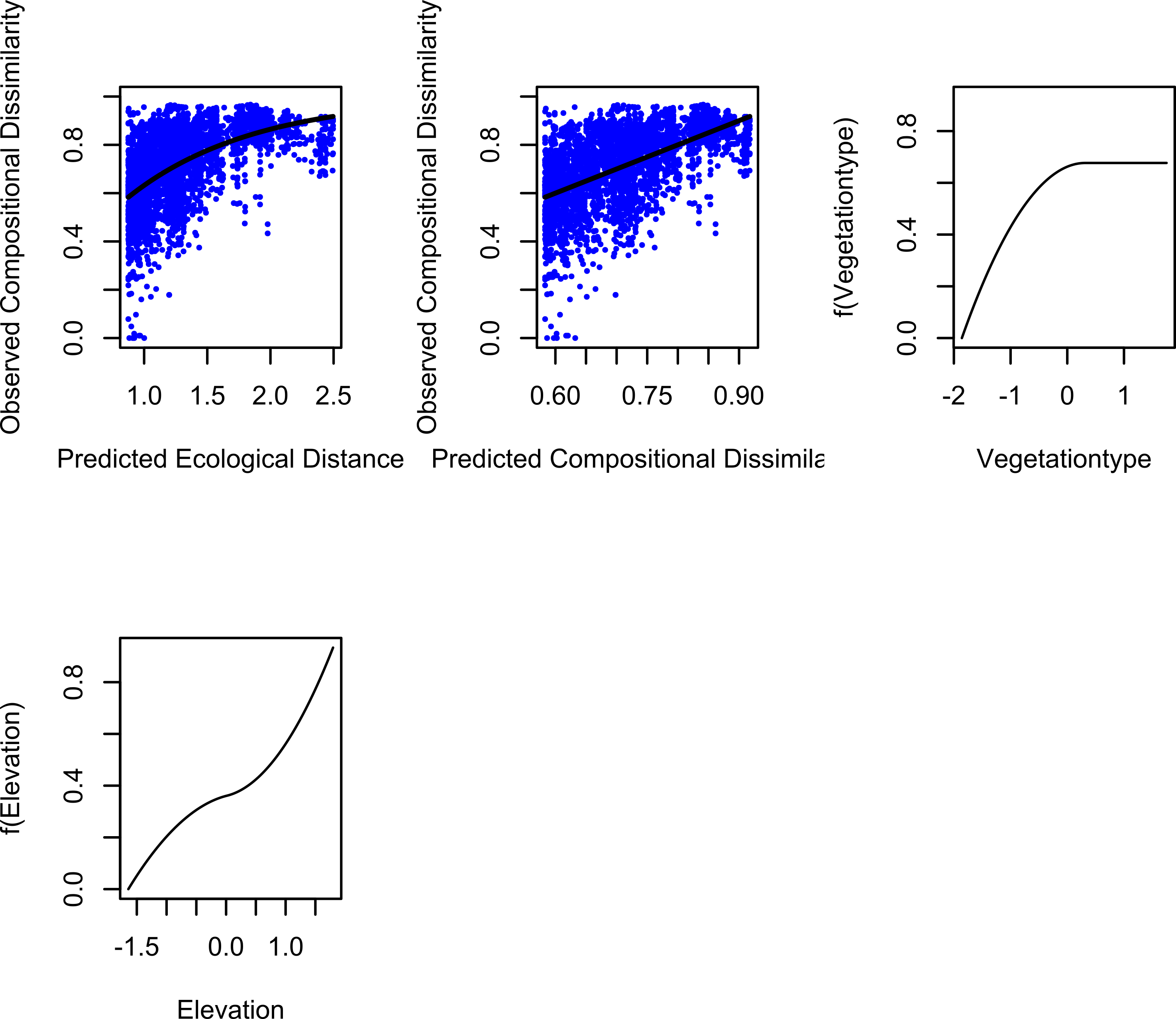


PDMosses

TDLiverworts

Figure S5. Response plots resulting from the Generalized dissimilarity modeling (GDM) models for the three facets (TD-Taxonomic diversity, FD-Functional diversity, and PD-Phylogenetic diversity) of β-diversity in the six groups (mosses, liverworts, acrocarpous mosses (acr), pleurocarpous mosses (ple), thalloid liverworts (tha), and leafy liverworts (lea)). Observed dissimilarity as a function of GDM-predicted ecological distance, with each site-pair represented as a point, and the line representing the GDM-predicted dissimilarity, the other plots represent GDM spline functions for each predictor variable. (continued on the next page)


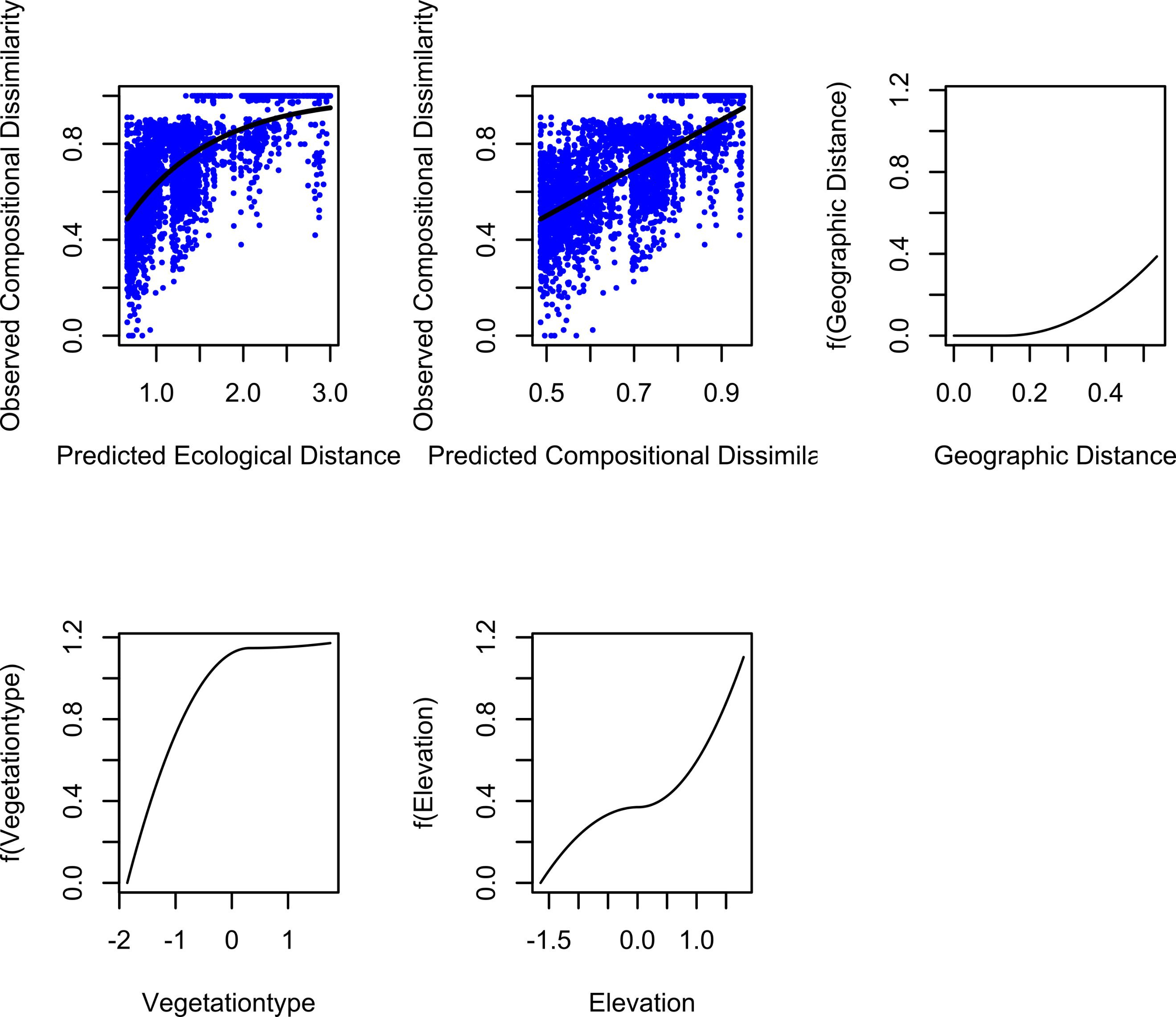

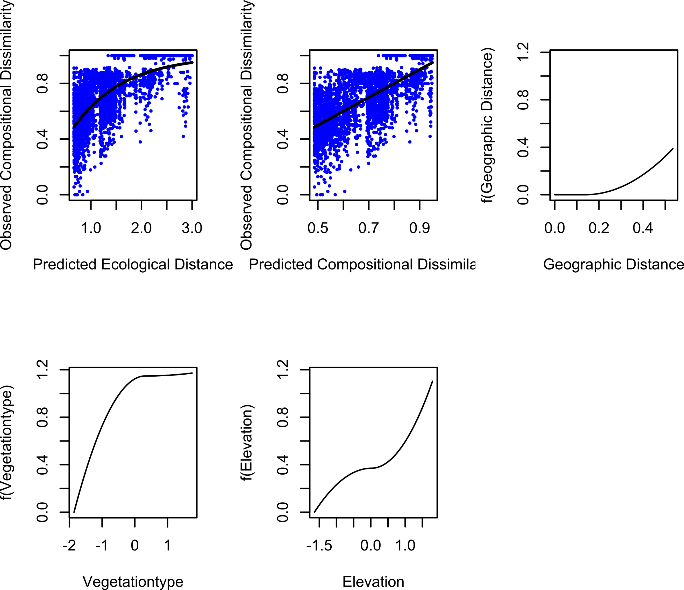


FDLiverworts

PDLiverworts


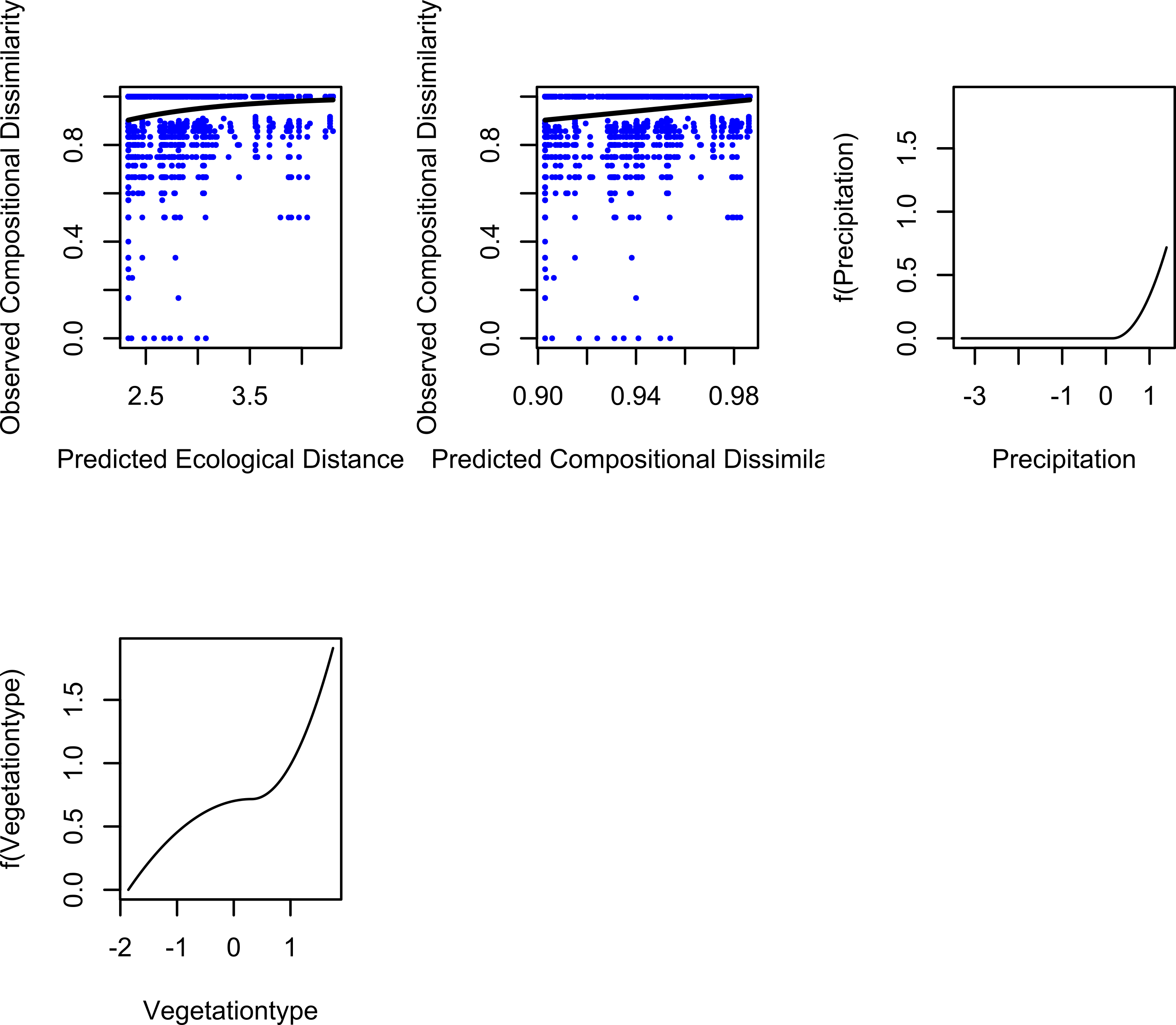

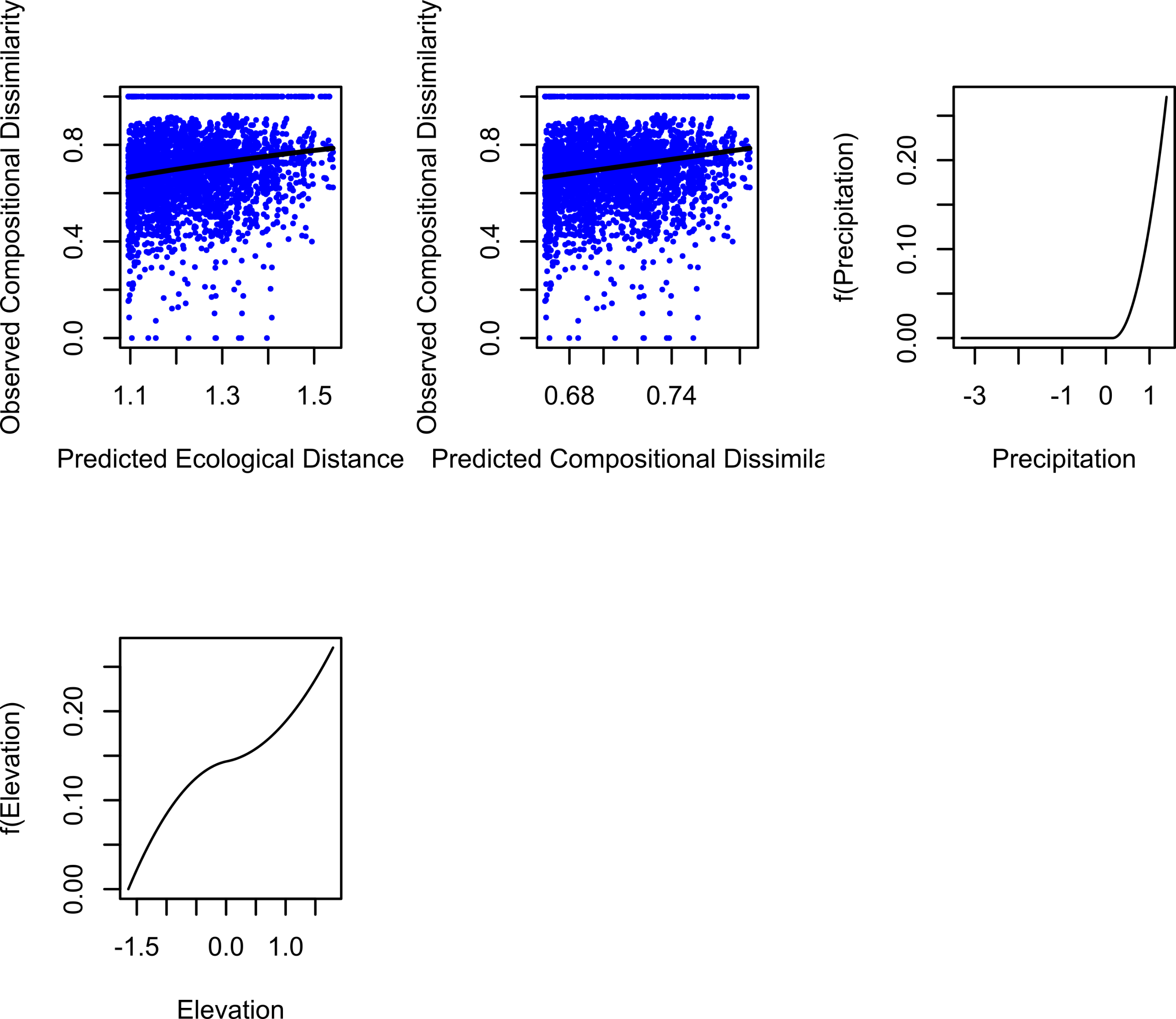


TDacr

FDacr

Figure S5. Response plots resulting from the Generalized dissimilarity modeling (GDM) models for the three facets (TD-Taxonomic diversity, FD-Functional diversity, and PD-Phylogenetic diversity) of β-diversity in the six groups (mosses, liverworts, acrocarpous mosses (acr), pleurocarpous mosses (ple), thalloid liverworts (tha), and leafy liverworts (lea)). Observed dissimilarity as a function of GDM-predicted ecological distance, with each site-pair represented as a point, and the line representing the GDM-predicted dissimilarity, the other plots represent GDM spline functions for each predictor variable. (continued on the next page)


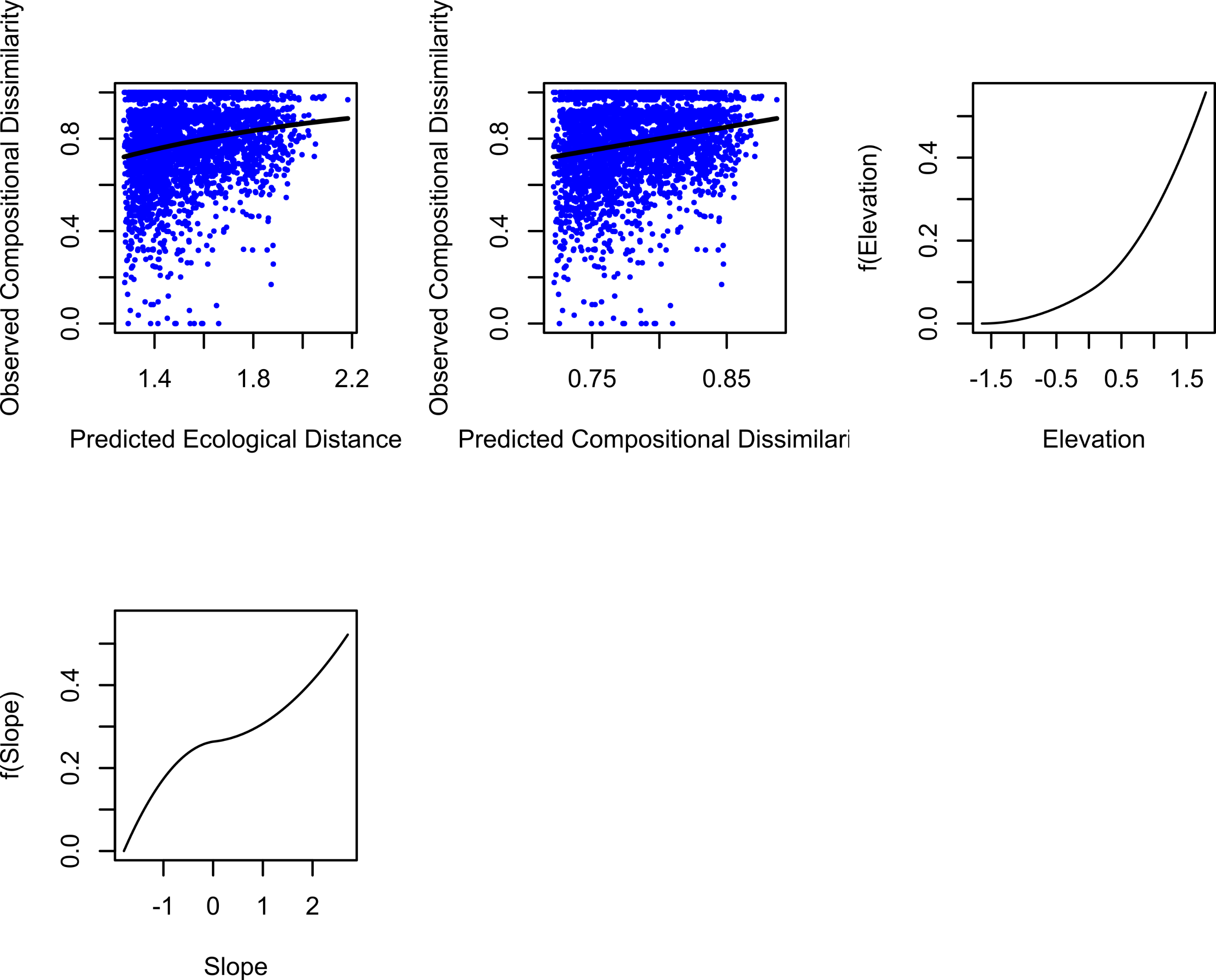


PDacr


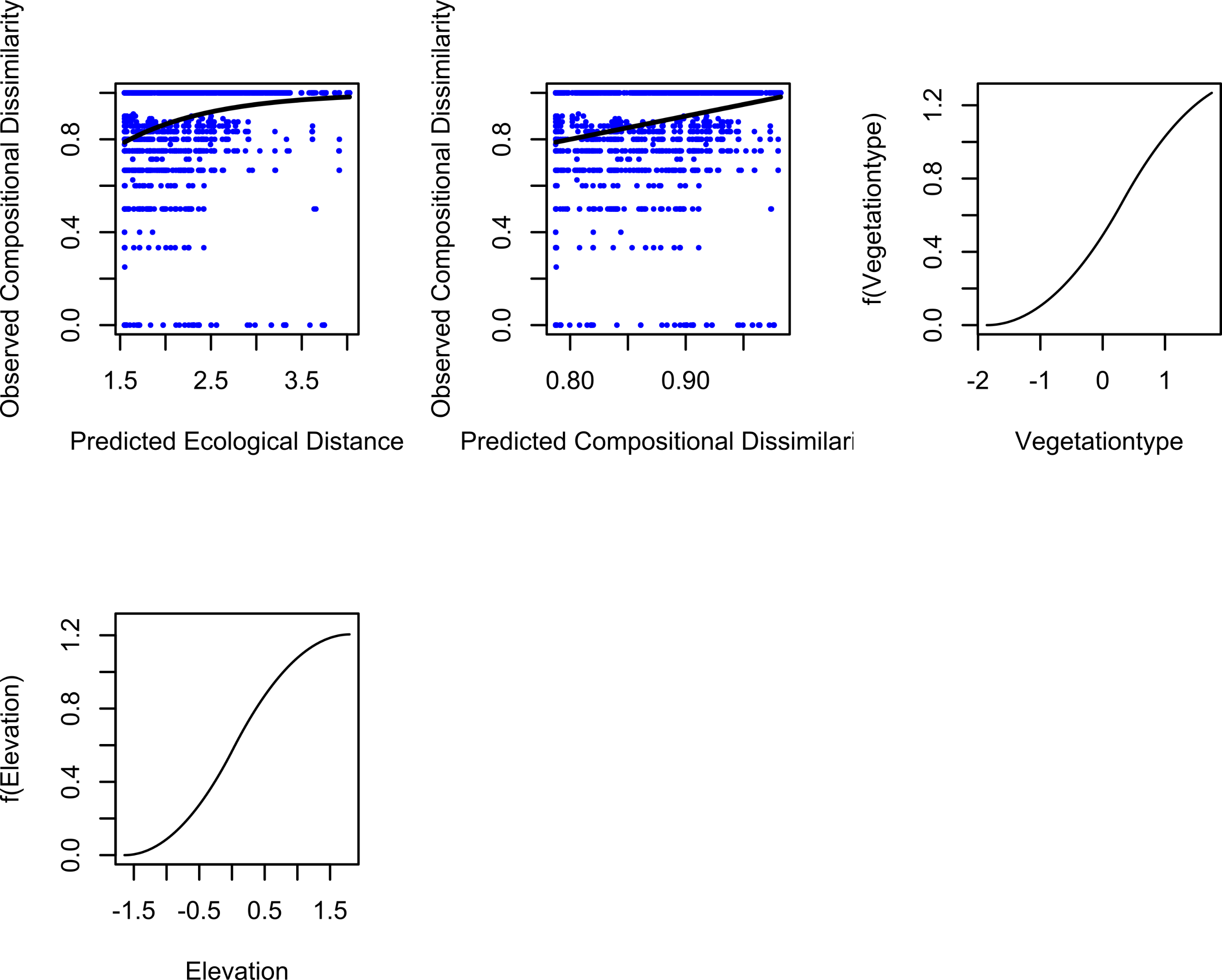


TDple


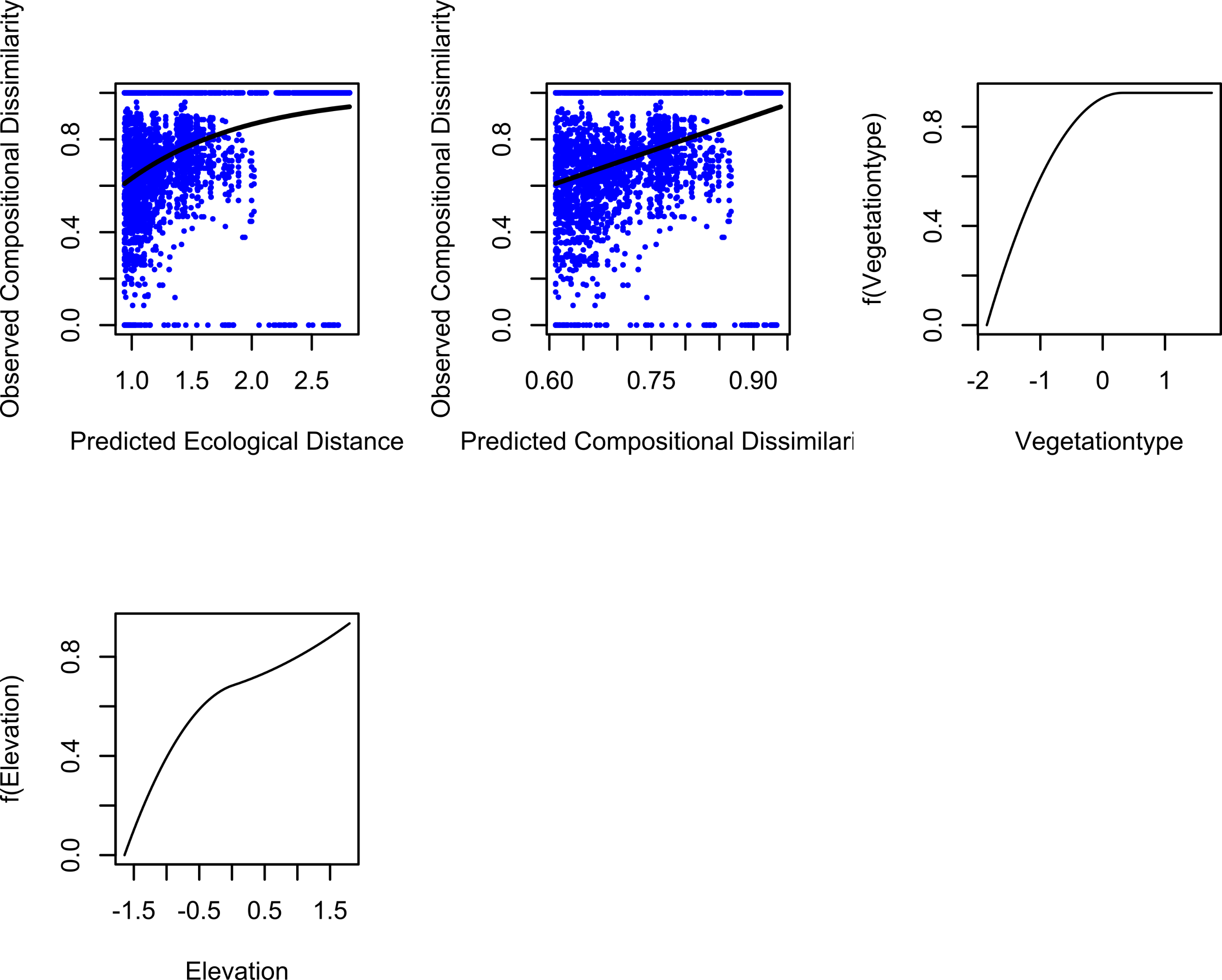

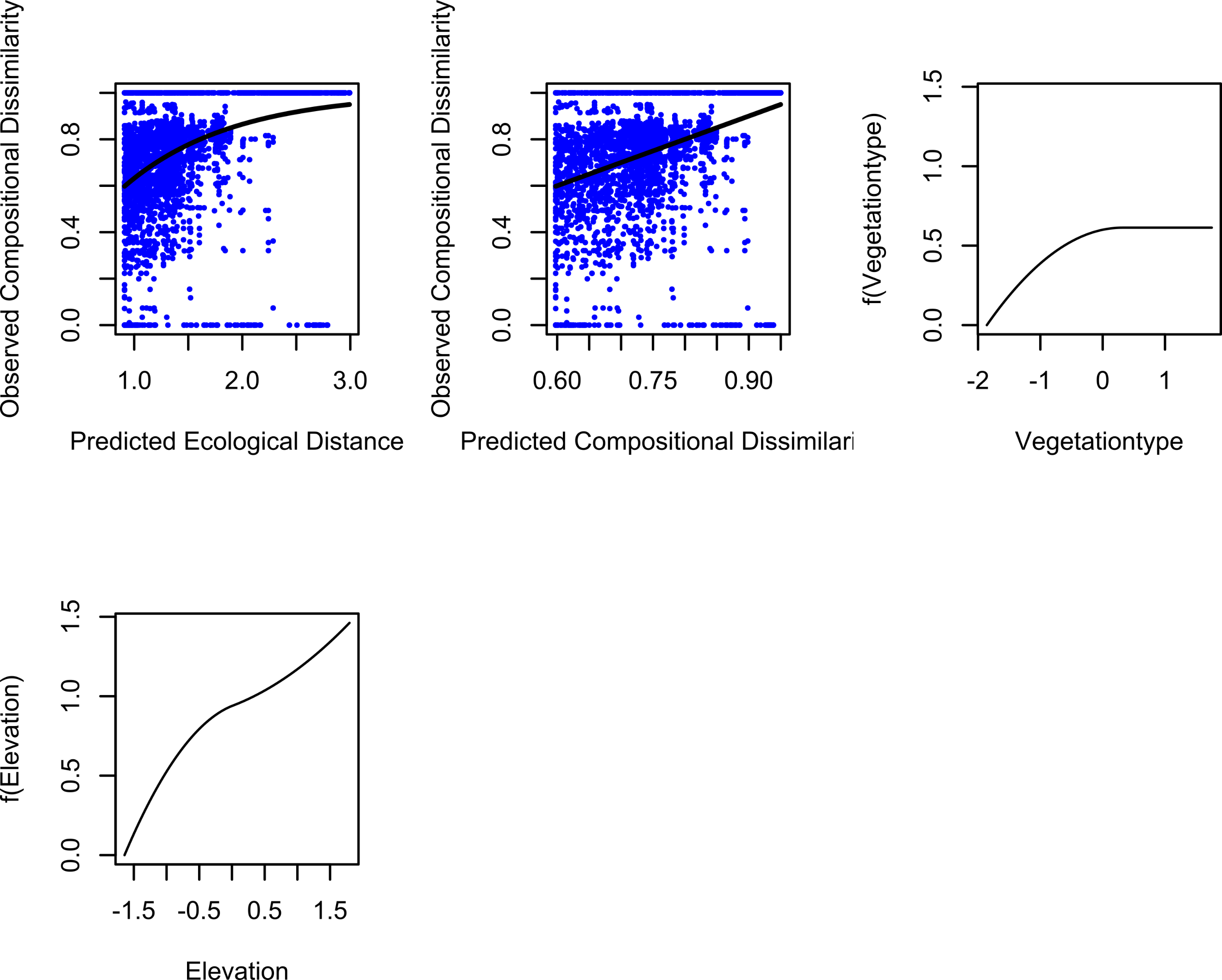


FDple

PDple

Figure S5. Response plots resulting from the Generalized dissimilarity modeling (GDM) models for the three facets (TD-Taxonomic diversity, FD-Functional diversity, and PD-Phylogenetic diversity) of β-diversity in the six groups (mosses, liverworts, acrocarpous mosses (acr), pleurocarpous mosses (ple), thalloid liverworts (tha), and leafy liverworts (lea)). Observed dissimilarity as a function of GDM-predicted ecological distance, with each site-pair represented as a point, and the line representing the GDM-predicted dissimilarity, the other plots represent GDM spline functions for each predictor variable. (continued on the next page)


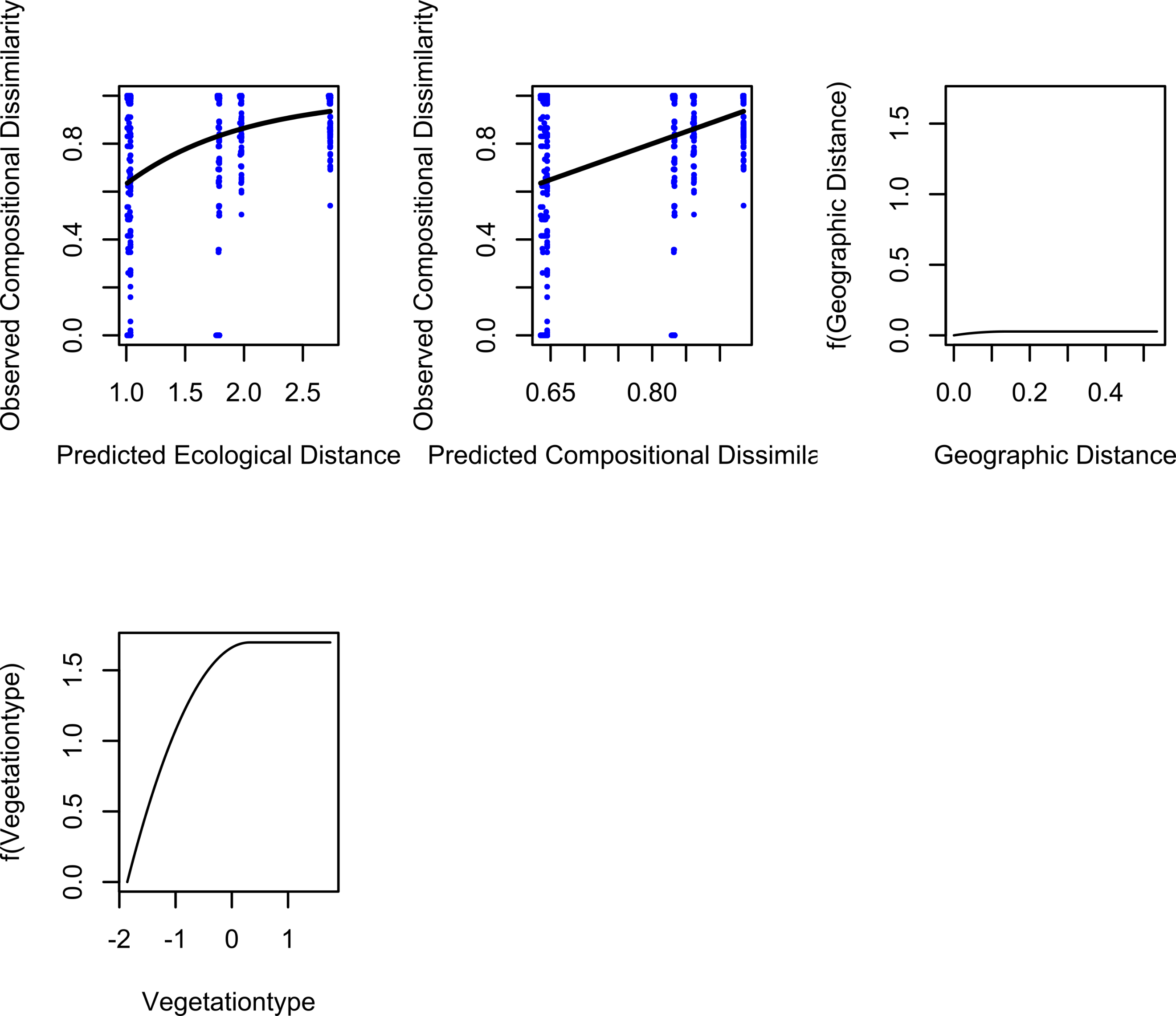

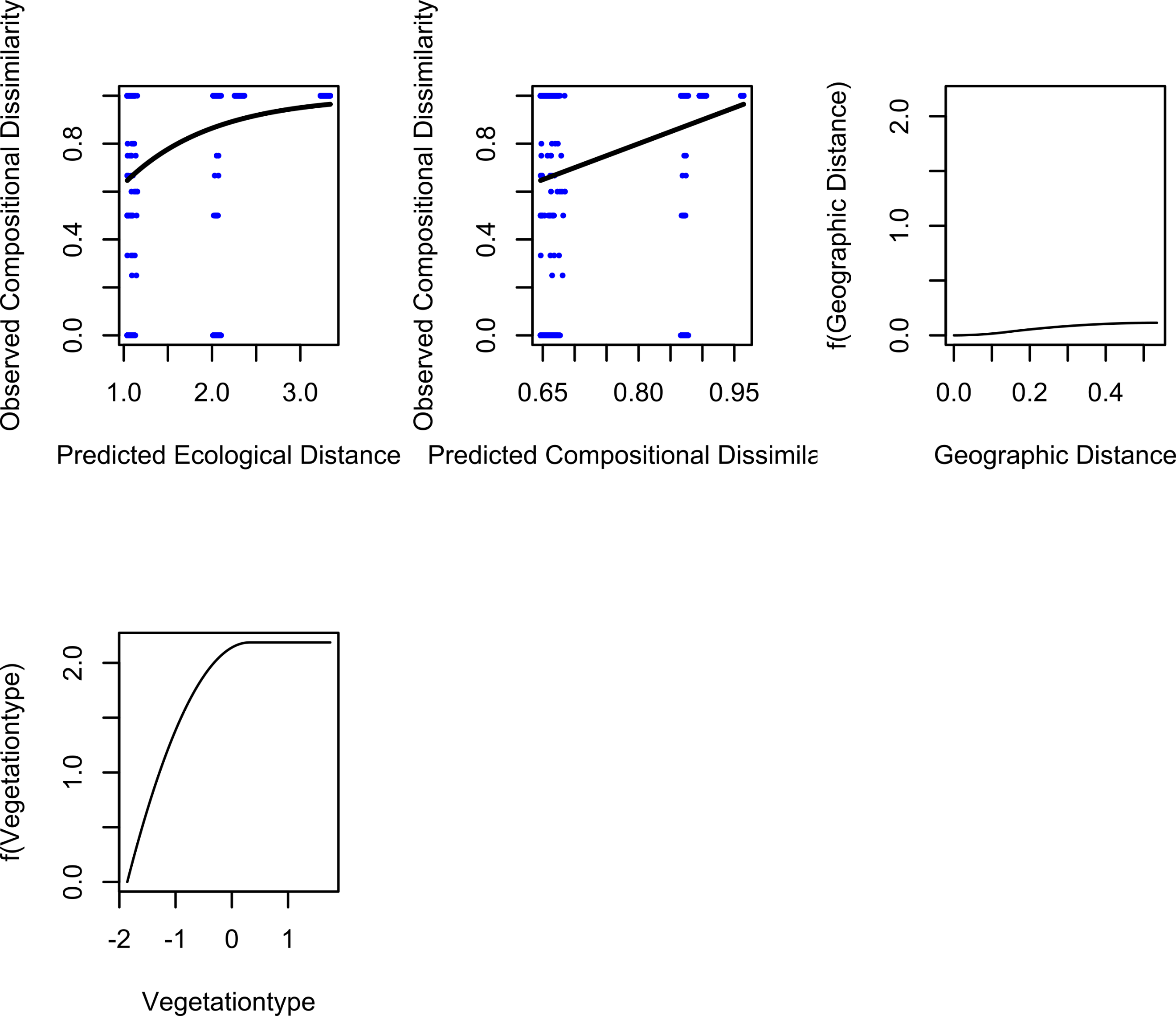

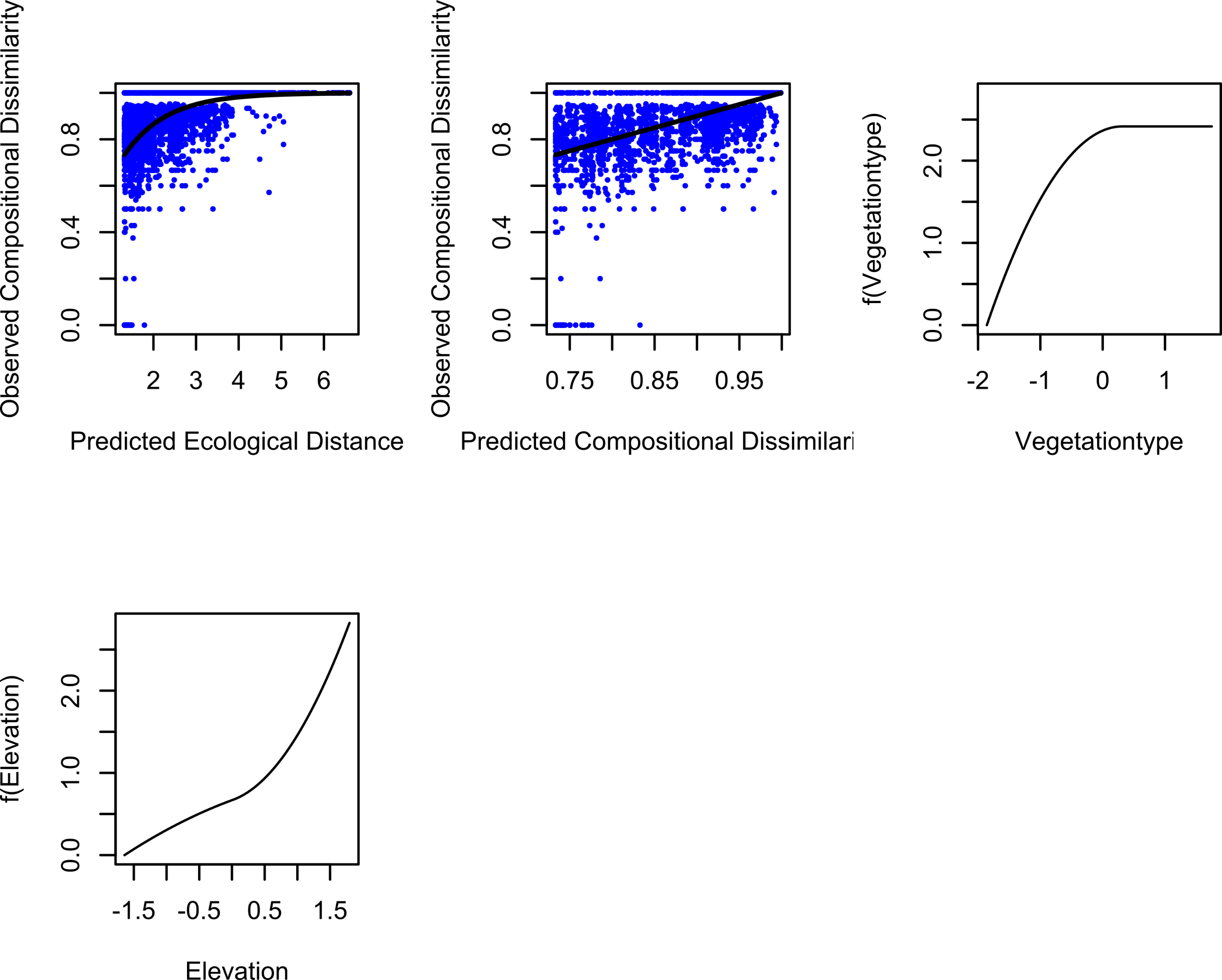

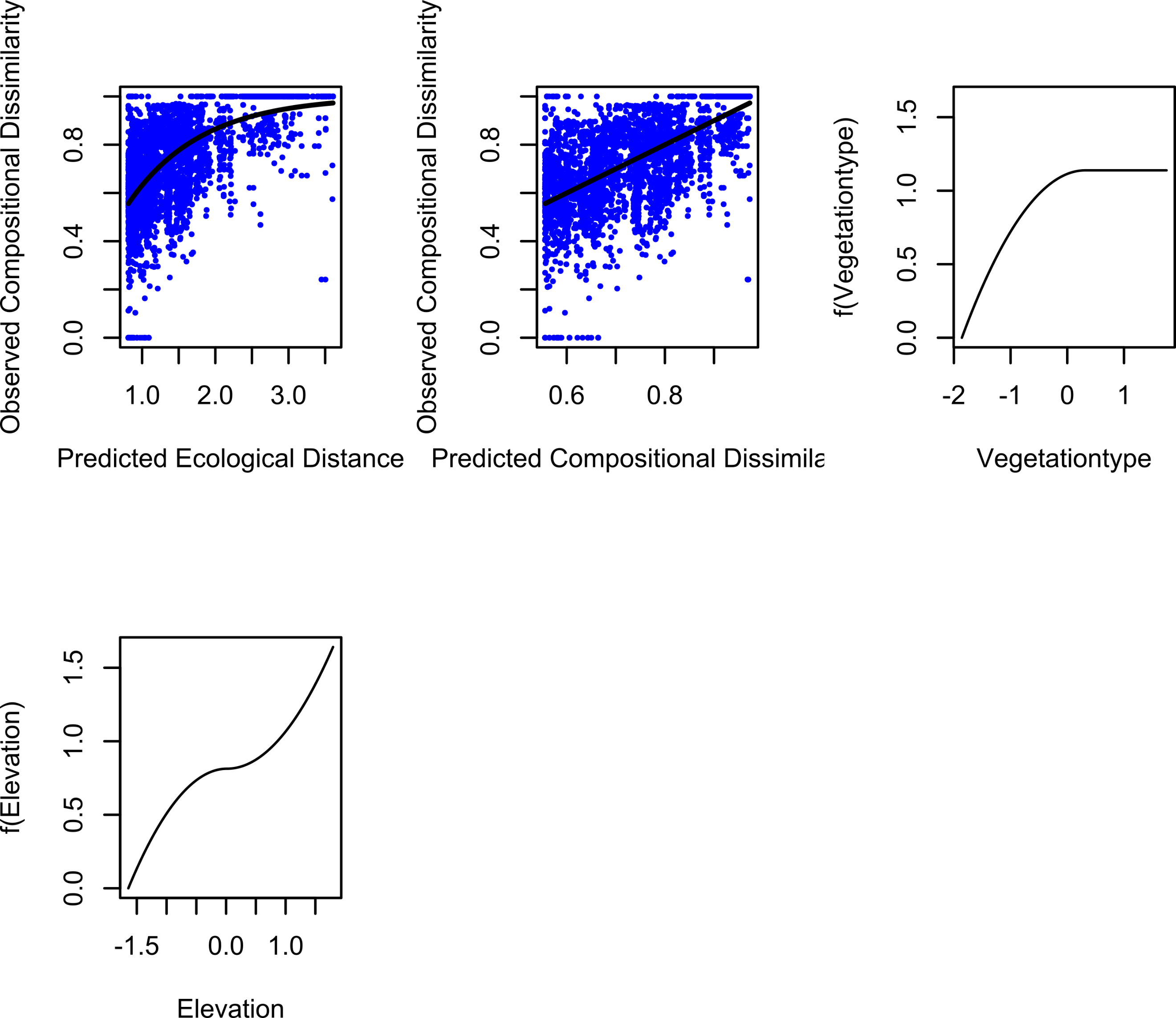


TDtha

FDtha

PDtha

TDlea

Figure S5. Response plots resulting from the Generalized dissimilarity modeling (GDM) models for the three facets (TD-Taxonomic diversity, FD-Functional diversity, and PD-Phylogenetic diversity) of β-diversity in the six groups (mosses, liverworts, acrocarpous mosses (acr), pleurocarpous mosses (ple), thalloid liverworts (tha), and leafy liverworts (lea)). Observed dissimilarity as a function of GDM-predicted ecological distance, with each site-pair represented as a point, and the line representing the GDM-predicted dissimilarity, the other plots represent GDM spline functions for each predictor variable. (continued on the next page)


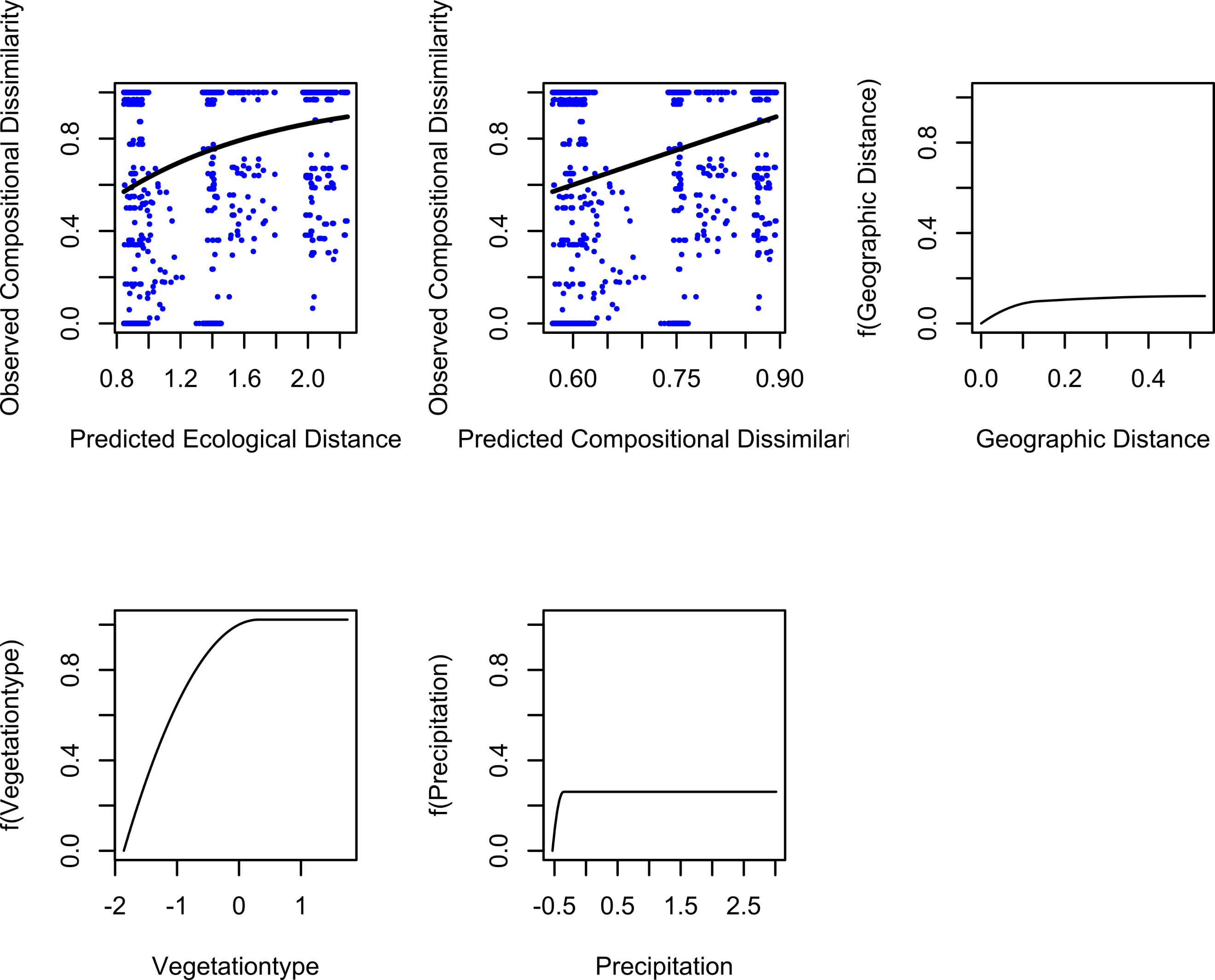

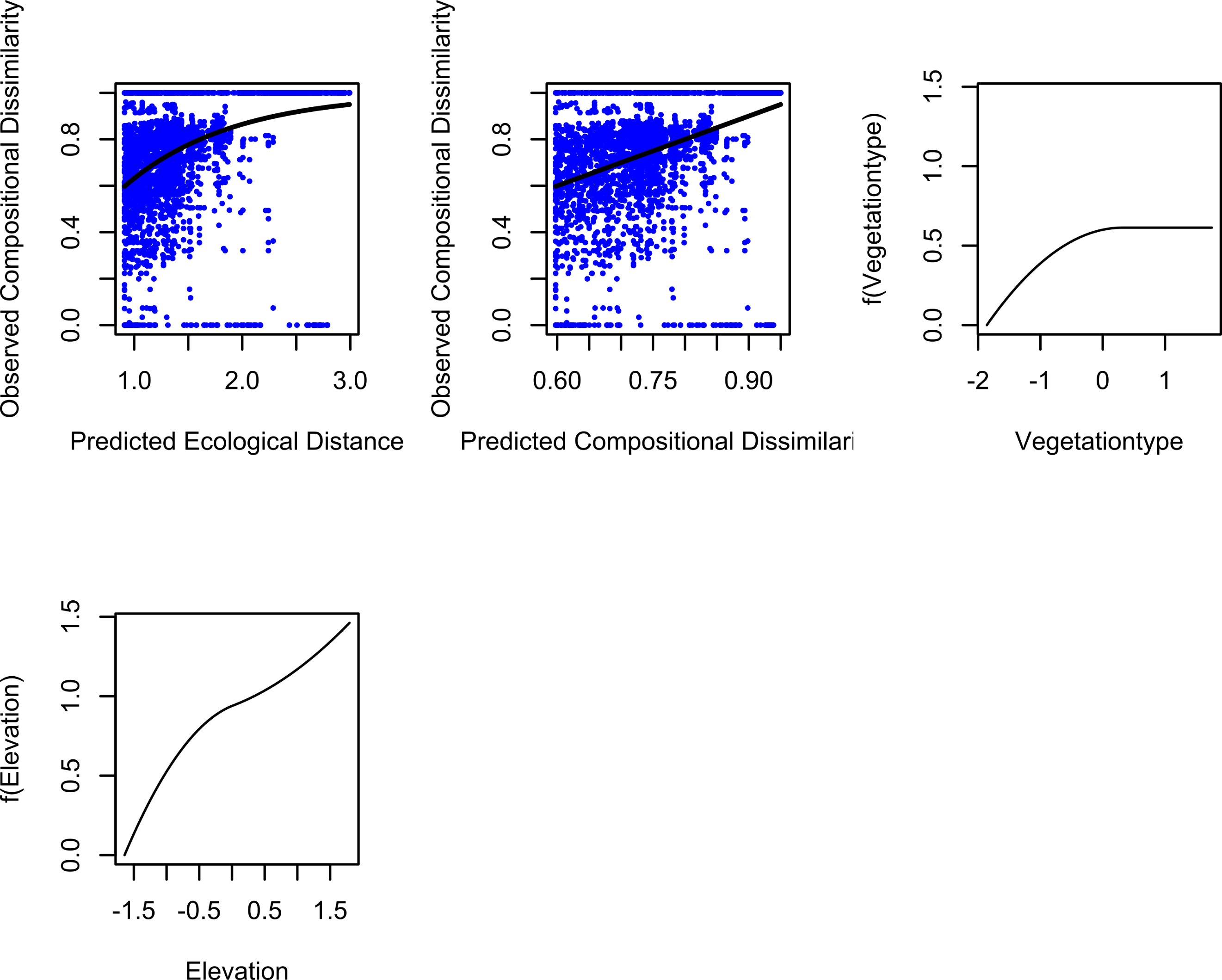


FDlea

PDlea

Figure S5. Response plots resulting from the Generalized dissimilarity modeling (GDM) models for the three facets (TD-Taxonomic diversity, FD-Functional diversity, and PD-Phylogenetic diversity) of β-diversity in the six groups (mosses, liverworts, acrocarpous mosses (acr), pleurocarpous mosses (ple), thalloid liverworts (tha), and leafy liverworts (lea)). Observed dissimilarity as a function of GDM-predicted ecological distance, with each site-pair represented as a point, and the line representing the GDM-predicted dissimilarity, the other plots represent GDM spline functions for each predictor variable. (continued on the next page)
